# Low-level predation by lytic phage phiIPLA-RODI promotes biofilm formation and triggers the stringent response in *Staphylococcus aureus*

**DOI:** 10.1038/srep40965

**Published:** 2017-01-19

**Authors:** Lucía Fernández, Silvia González, Ana Belén Campelo, Beatriz Martínez, Ana Rodríguez, Pilar García

**Affiliations:** 1 Instituto de Productos Lácteos de Asturias (IPLA-CSIC), Paseo Río Linares s/n 33300, Villaviciosa, Asturias, Spain

## Abstract

An important lesson from the war on pathogenic bacteria has been the need to understand the physiological responses and evolution of natural microbial communities. Bacterial populations in the environment are generally forming biofilms subject to some level of phage predation. These multicellular communities are notoriously resistant to antimicrobials and, consequently, very difficult to eradicate. This has sparked the search for new therapeutic alternatives, including phage therapy. This study demonstrates that *S. aureus* biofilms formed in the presence of a non-lethal dose of phage phiIPLA-RODI exhibit a unique physiological state that could potentially benefit both the host and the predator. Thus, biofilms formed under phage pressure are thicker and have a greater DNA content. Also, the virus-infected biofilm displayed major transcriptional differences compared to an untreated control. Significantly, RNA-seq data revealed activation of the stringent response, which could slow down the advance of the bacteriophage within the biofilm. The end result would be an equilibrium that would help bacterial cells to withstand environmental challenges, while maintaining a reservoir of sensitive bacterial cells available to the phage upon reactivation of the dormant carrier population.

The arms race between bacteriophages and their bacterial hosts is a major evolutionary driving force in the microbial world^1^,^2^. It is now widely recognized that phage predation contributes significantly to shaping bacterial communities^3^,^4^. For instance, bacteriophages can favor growth of certain species or strains in a given ecosystem, as well as facilitate DNA exchange between bacterial cells. Also, exposure to phages may lead to transcriptional changes in the prey population^5^,^6^,^7^,^8^,^9^. As a result, attaining a better understanding of the dynamics between bacteria and phages is of the utmost importance from an ecological perspective. Furthermore, this information would be valuable to design improved strategies for the control of undesired microorganisms. However, the currently available information regarding bacteria-phage interactions is still somewhat limited^2^. In addition to their environmental significance, bacteriophages are also promising therapeutics. Indeed, phage therapy has recently regained attention given the relentless rise in the resistance of pathogenic bacteria to more conventional antimicrobials^10^. Bacteriophages have been used in some Eastern European countries like Russia and Georgia for decades^11^, but the costs necessary to comply with regulatory burdens have probably hindered their use in Western medicine. Interestingly, numerous studies indicate that phages can help to eliminate biofilms^12^,^13^,^14^, which are an important challenge for antibacterial strategies due to their high resistance. Moreover, some authors indicate that phages are able to lyse biofilm-embedded bacteria even when they have become resistant to antibiotics^15^.

Biofilms represent the most ubiquitous mode of bacterial growth in natural and artificial environments^16^,^17^. In the clinic, biofilms can develop on surfaces and implant devices as well as on living tissues, being a major problem in chronic recalcitrant infections. The opportunistic Gram-positive pathogen *Staphylococcus aureus* is a good example of an adept biofilm former^18^. Indeed, this bacterium does not only form biofilms in hospital settings, but also in the food industry. As a result, elimination of this pathogen by routine disinfection procedures can be quite difficult. In food environments, this further allows for the accumulation of enterotoxins produced by *S. aureus* and subsequent contamination of foodstuffs. In *S. aureus* biofilms, the cells are embedded in an extracellular matrix consisting of polysaccharides, proteins and extracellular DNA (eDNA), although the proportion of these components depends on the specific strain and growth conditions^18^. Besides their biofilm-forming capacity, some *S. aureus* strains are resistant to most antibiotics available today. Of particular relevance are the so-called methicillin-resistant *S. aureus* (MRSA) and vancomycin-resistant *S. aureus* (VRSA) strains^19^. There are several studies demonstrating the successful utilization of bacteriophages against *S. aureus*^20^,^21^,^22^. For example, the lytic phage vB_SauM_phiIPLA-RODI (phiIPLA-RODI), belonging to the *Myoviridae* family, has been recently isolated from sewage samples and has shown promising results for the disruption of staphylococcal biofilms^21^.

The aim of this study was to characterize the physiological properties and transcriptional profile of *S. aureus* biofilm cells exposed to phage phiIPLA-RODI. The results presented here show that low-level exposure to phages can create a biofilm-enhancing environment that protects *S. aureus* cells from complete eradication. Furthermore, cells within phage-containing biofilms show a distinct expression pattern that indicates activation of the stringent response. To our knowledge, this is the first transcriptomic analysis of phage-infected biofilm cells. Bacteriophage pressure in sessile communities is common place not only in nature, but also in man-made environments, including surfaces in hospitals and the food industry. Also very importantly, the information provided here can help to predict possible scenarios that may occur following the application of phage therapy if the viral dose does not completely eradicate the biofilm.

## Results

### Biofilm formation is enhanced by infection with sub-inhibitory doses of phiIPLA-RODI

In order to study the effect of phage exposure on biofilm formation, we first followed bacterial growth in both the planktonic and the adhered phase in the presence of increasing multiplicities of infection (MOIs, 10^−6^ to 10^−2^) of the lytic bacteriophage phiIPLA-RODI. *S. aureus* IPLA 1, a strain of dairy origin, was chosen for these experiments due to its sensitivity to this phage and because it exhibits a medium-level biofilm forming capacity. This facilitated the observation of changes in adhered biomass by crystal violet staining.

**Figure 1:**
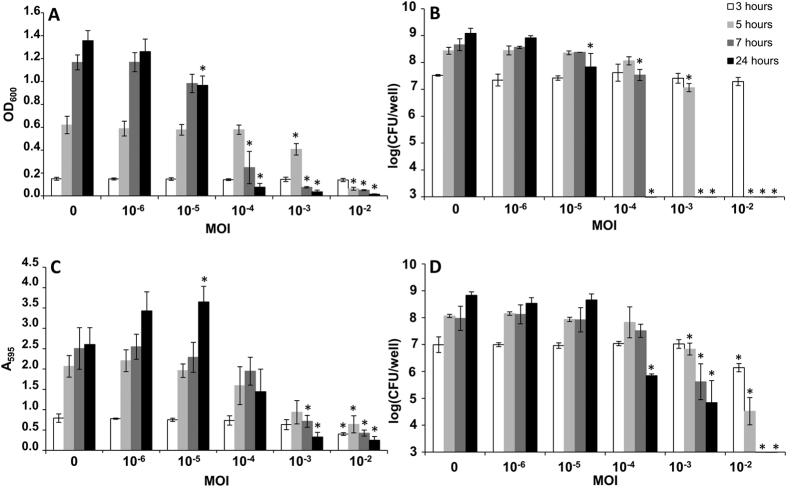
Biofilm formation and planktonic growth of *S. aureus* IPLA 1 in the presence of different MOIs of bacteriophage phiIPLA-RODI. (**A**) Planktonic growth measured as OD_600_. (**B**) Viable cell numbers per well present in the planktonic phase. (**C**) Adhered biomass determined as absorbance at 595 nm (A_595_) following crystal violet staining. (**D**) Viable cell numbers per well in the adhered phase (biofilm). All values represent the average and standard deviation of three independent biological repeats. Data obtained in the presence of different phage concentrations were compared to the control samples taken at the same time point. *P*-values < 0.05 were considered significant (*). Bars missing correspond to bacterial counts below the detection limit (10 CFU/well).

At all the MOIs tested, bacterial growth in the planktonic phase was equal to the non-infected control until three hours post-infection, reaching OD_600_ values of approximately 0.16 (Fig. 1A). Two hours later, the samples infected with an MOI of 10^−2^ showed a considerable reduction in OD_600_ (Fig. 1A) and there were no remaining viable cells (Fig. 1B). This indicates the occurrence of cell lysis due to the phage. The same occurred at MOIs 10^−3^ and 10^−4^ at 7 and 24 hours post-infection, respectively. In contrast, viable cells could be detected at all time points for the lower MOIs tested, namely 10^−5^ and 10^−6^. However, a one-log reduction was observed in the planktonic phase of the 24-hour biofilm grown with an MOI of 10^−5^ compared to the control without phage. Regarding the adhered phase (biofilm), there were some major differences compared to the results observed for the planktonic phase. Interestingly, biomass quantification by crystal violet staining revealed a significant (*P*-value = 0.003) increase in biofilm formation after 24 hours of incubation in the sample inoculated with an MOI of 10^−5^ (Fig. 1C). Additionally, at the 24-hour time point, viable cells could be observed even at an MOI of 10^−3^ and there was no significant (*P*-value = 0.107) decrease in cell counts at an MOI of 10^−5^ (Fig. 1D). Biofilm formation in the presence of an MOI of 10^−3^ was also monitored by confocal laser scanning microscopy. Timing of bacterial growth and cell lysis by phiIPLA-RODI confirmed the observations described above (Supplementary video S1).

Considering the data gathered at the 24-hour time point, the minimum inhibitory MOI was determined to be 10^−3^ (10^3^ PFU/well), whereas the minimum bactericidal MOI was 10^−2^ (10^4^ PFU/well).

All subsequent experiments aimed at characterizing biofilms grown under phage predation were performed at an MOI of 10^−5^ (10 PFU/well). These conditions were selected because there was no major reduction in viable cell numbers, which would alter the expression of the quorum sensing regulon. This would, in turn, mask transcriptional changes specifically due to attack by the phage. Moreover, we intended on studying more in depth the characteristics of the enhanced biofilm formed in the presence of this low viral dose.

### Disaggregation of phage-containing biofilms leads to generalized cell lysis

**Figure 2:**
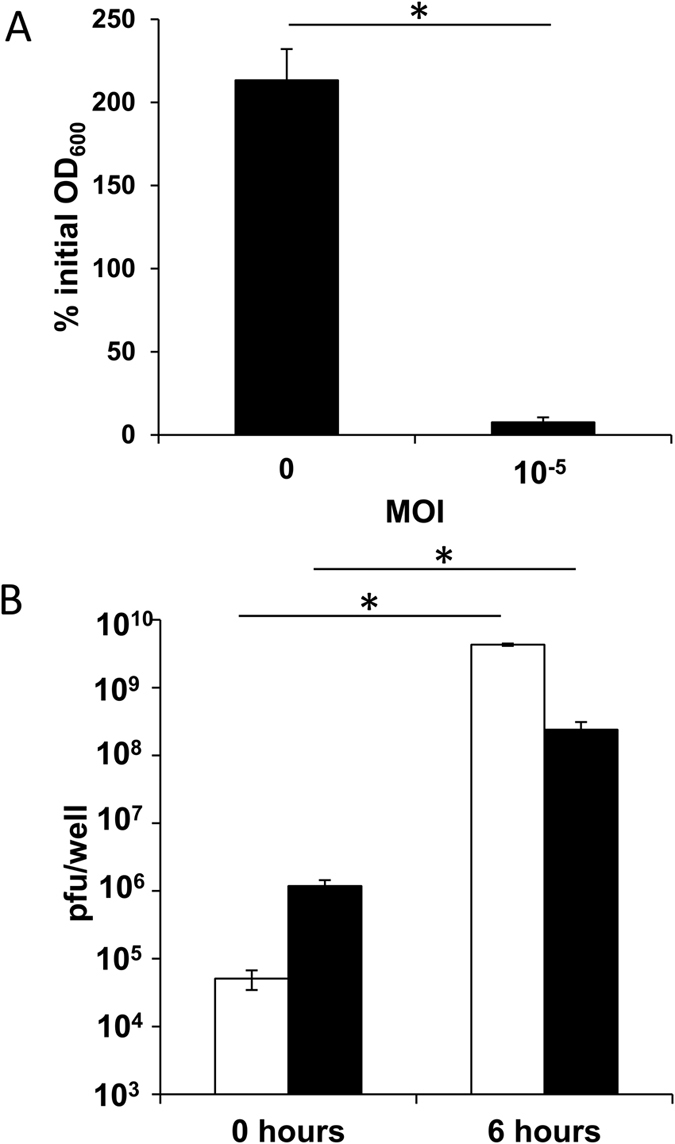
Evolution of cell density and phage titer after disaggregation of 24-hour biofilms of *S. aureus* IPLA 1 formed in the absence or presence of phiIPLA-RODI. (**A**) Percentage of the initial OD_600_ after 6 hours of incubation for biofilms grown without phage or in the presence of phage at an MOI of 10^−5^. (**B**) Phage titer determined before and after 6 hours of incubation at 37 °C in biofilms formed in the presence of phiIPLA-RODI at an MOI of 10^−5^. White and black bars correspond to phage titer of the matrix and the adhered cells, respectively. Values represent the average and standard deviation of 3 independent biological repeats. *P-values < 0.05.

Before continuing the analysis of phage-treated biofilms, it was necessary to confirm that cells remained sensitive to phiIPLA-RODI. To do that, adhered cells were taken from the control and the infected biofilms and subsequently resuspended in fresh TSB-g medium. Cell density was measured as OD_600_ before and after incubation for 6 hours at 37 °C. At the end of the experiment, OD_600_ values had doubled in the control sample, whereas cell density was drastically reduced, by more than 90%, in the sample corresponding to the phage-infected biofilm (Fig. 2A). This demonstrated that the cells in the treated biofilm were susceptible to phage infection, resulting in cell lysis. Consequently, differences between the treated and the control samples would not be due to the selection of phage resistant mutants.

Additionally, the number of plaque-forming units (PFU) associated to cells or free in the extracellular matrix was determined before and after the 6-hour incubation of the infected sample. The total phage count at time point 0 was 10^6^ PFU/well, which is a considerable increase from the starting inoculum of 10 PFU/well (Fig. 2B). It is noteworthy that the majority of the viral particles were associated to cells and not free in the extracellular matrix. In the postincubation sample, most infecting particles were free in the supernatant while only about 1% was cell-associated. This result is to be expected given the high level of lysis observed. Also, the total phage titer at the end of the experiment was 4 × 10^9^ PFU/well, which demonstrates that there was propagation and not just lysis of the already infected cells (Fig. 2B). These results suggest that the viable cell counts corresponding to the treated biofilms may actually represent the number of cells not infected by the phage. In addition to these, the biofilm community would consist of a small but significant subset of viral carrier cells that would restart the lytic cycle upon reactivation.

### Biofilms formed in the presence of subinhibitory MOIs of phiIPLA-RODI exhibit structural changes

**Figure 3:**
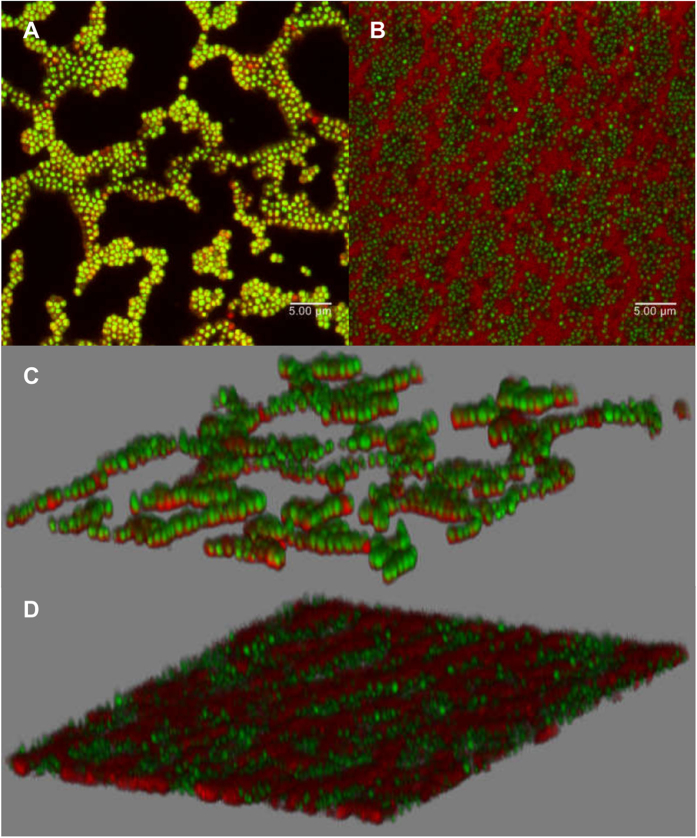
Confocal laser scanning microscopy images showing 24-hour biofilms formed in the presence or not of phiIPLA-RODI at an MOI of 10^−5^. (**A** and **C**) untreated IPLA 1 biofilm. (**B** and **D**) IPLA1 biofilm formed with phage. Samples were stained with SYTO^®^ 9 and PI. Green represents live cells and red represents dead cells or eDNA.

Confocal laser scanning microscopy (CLSM) was used to further study the differences between 24-hour biofilms grown without phage predation and in the presence of a subinhibitory MOI of 10^−5^. After incubation, the adhered cells were stained with SYTO^®^ 9 and propidium iodide (PI) to distinguish between live cells (green), and damaged cells or eDNA (red). The control sample showed a typical “island-like” structure composed of different layers, in which most damaged cells (red) were located near the bottom (Fig. 3A and C). In contrast, the sample developed in the presence of phiIPLA-RODI exhibited a remarkably different architecture. Thus, cells covered a greater surface of the glass well, but seemed to be organized in a flatter structure (Fig. 3B and D). Also, a red net-like pattern surrounding the cells could be observed, which would very likely correspond to eDNA. Nevertheless, the increased abundance of eDNA in the treated versus the control biofilms remained to be confirmed.

**Figure 4:**
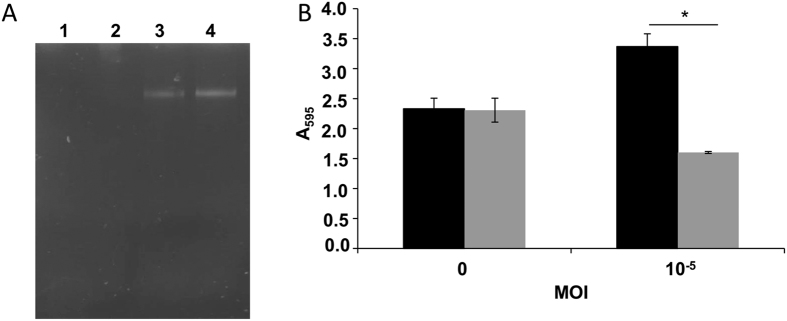
Comparison of eDNA content between untreated biofilms and those formed in the presence of low phage concentrations. (**A**) Agarose gel visualization of eDNA prepared from untreated biofilms (lanes 1 and 2) and biofilms formed in the presence of phiIPLA-RODI (MOI of 10^−5^) (lanes 3 and 4). (**B**) DNAse treatment of 24 hour-biofilms developed without phage or with an MOI of 10^−5^. Black and grey bars represent biofilms incubated without or with 200 μg/ml DNAse I for one hour at 37 °C. Values represent the average and standard deviation of four independent biological replicates. *P-values < 0.05.

Based on the observations made by microscopy, the next step was to demonstrate that bacteriophage-infected biofilms were richer in eDNA. First, the DNA present in the extracellular matrix was obtained from biofilms subject to low phage predation and non-treated controls. Agarose gel electrophoresis of these samples showed that those biofilms developed in the presence of phiIPLA-RODI contained more DNA than control biofilms of *S. aureus* IPLA 1 (Fig. 4A). Additionally, biofilms formed under phage predation were more sensitive to DNAse treatment than their non-infected counterparts (Fig. 4B). Indeed, the biomass of control biofilms hardly changed during a 2-hour DNAse treatment. In contrast, the biomass of the phage-containing biofilm was reduced to approximately 47%. These results demonstrate that the biofilms formed under low-phage predation have a different extracellular matrix, containing a greater proportion of eDNA. These biofilms seem to be more stable and better withstand the washing steps of crystal violet staining, resulting in increased attached biomass.

### The *S. aureus* IPLA 1 biofilm population exhibits a distinct transcriptional profile under low-level phage predation

**Figure 5:**
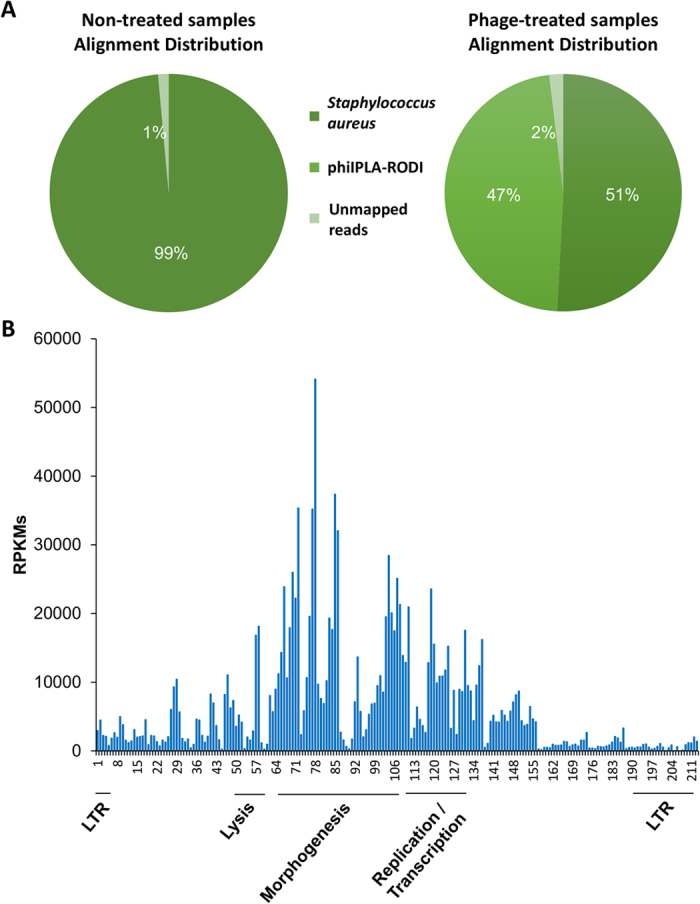
Transcriptomic analysis by RNA-seq of biofilms exposed to phiIPLA-RODI compared to untreated controls. (**A**) Average alignment distribution of three biological repeats of samples untreated or treated with phage phiIPLA-RODI. (**B**) normalized mean reads per kilobase million (RPKM) values corresponding to the different open reading frames (ORFs) of phiIPLA-RODI genome in the treated samples.

Given the phenotypical differences observed between the infected and uninfected biofilms, it appeared important to discern if they also displayed distinct transcriptional profiles. To determine if that was the case, the transcriptome of 24-hour biofilms formed in the presence or absence of a low dose of phiIPLA-RODI (10 PFU/well, corresponding to an MOI of 10^−5^) was analysed through RNA-seq. Transcripts were then aligned with the *S. aureus* NCTC 8325 and phage phiIPLA-RODI genomes. In the control sample, 99% of sequences aligned with *S. aureus* NCTC8325 (Fig. 5A). However, only 51% aligned with the *S. aureus* reference genome in the bacteriophage-treated samples, with an additional 47% mapping to phiIPLA-RODI sequences (Fig. 5A).

Regarding bacteriophage gene expression, further analysis revealed that the genes showing the highest expression were located within the morphogenesis module (Fig. 5B, Table S1). Nonetheless, significant transcription was also observed for genes involved in lysis and replication/transcription (Fig. 5B, Table S1). These results were not surprising as the biofilm is not reflective of a synchronized infection. Therefore, it would be expected to harvest the infected cells at different stages of phage development. Also, in a previous study performed on *Lactococcus lactis*, a similar transcriptional profile was observed for phages Tuc2009 and c2 at late stages of the lytic cycle^5^.

Regarding *S. aureus* genes, a total of 1063 transcripts showed significant changes (adjusted *P*-values < 0.01) between the two samples, of which 548 were downregulated and 515 were upregulated in the infected biofilms (Table S2). Interestingly, a subset of these genes indicated activation of the stringent response. Indeed, 76 genes (Table 1) showed the same regulation pattern observed in microarray analyses of the stringent response regulon performed by Anderson *et al*.^23^ and Reiß *et al*.^24^. For instance, the bifunctional (p)ppGpp synthase and hydrolase RSH-encoding gene, a homolog of *relA*/*spoT*, is upregulated 4.9-fold. Another (p)ppGpp synthase gene, *relP*, was also upregulated by 2.21-fold in the phage-treated sample. This synthase, however, is not usually part of the stringent response and is induced by cell wall targeting antimicrobials^25^. The stringent response is typically triggered by amino acid starvation and tends to inhibit protein synthesis and promote amino acid biosynthesis. Other known triggers of this response are osmotic stress, carbon source starvation and depletion of fatty acids. The alarmone (p)ppGpp has been shown to participate in diverse processes such as biofilm development, sporulation, entry in the stationary phase and persistence, as well as virulence, particularly during chronic infections, and tolerance to antimicrobials^26^,^27^,^28^,^29^.

Amongst the genes typically regulated by (p)ppGpp also worth mentioning are those encoding ribosomal proteins, which were downregulated (between 2- and 3-fold) in the phage-treated sample. Many genes encoding other proteins involved in the translation machinery were also downregulated, including several tRNA synthase-encoding genes (*lysS, metG, pheS, aspS, valS*) and the translation initiation factor-encoding gene *infB*. However, other genes coding for tRNA synthases were upregulated, namely *argS, trpS* and *tyrS*. Regarding aminoacid metabolism genes, most showed increased expression (*dapB, hom, hisC, asd, hisZ*), while others were repressed (*arcC2, argF, arcA*). For instance, *asd*, the gene encoding the aspartate-semialdehyde dehydrogenase was upregulated by 4.3-fold. This enzyme participates in the biosynthesis of lysine, methionine, leucine and isoleucine from aspartate. Another product of this pathway is diaminopimelate, which is essential for bacterial cell wall formation. Further overlaps with the stringent response regulon included genes coding for proteins involved in energy metabolism such as ATP synthetase (*atpB, atpC, atpD, atpE, atpG*) and quinol oxidase (*qoxA-D*) subunits.

Interestingly, the gene encoding the major autolysin AtlA was upregulated by 5.42-fold. Also sortase A-encoding gene *srtA* was induced 2.95-fold. These two genes overlapped with the stringent response microarrays, confirming that they are part of the (p)ppGpp regulon in *S. aureus* IPLA 1. AtlA participates in cell wall metabolism, while SrtA participates in the processing of surface proteins. There were other changes in genes affecting cell wall synthesis and metabolism. For instance, several genes related to biosynthesis of peptidoglycan were downregulated (*glmM, glmU* and *glmS*). In contrast, some capsule-related genes were slightly upregulated, as were genes involved in D-alanylation of teichoic acids (*dltA, dltB* and *dltC*). In *S. aureus*, D-alanylation of teichoic acids plays an important role in adherence and, as a result, contributes to the initial stages of biofilm formation. Indeed, strains harboring mutations in the *dlt* operon cannot attach to surfaces or form biofilms^30^. The *dlt* operon can be induced by cations or cationic antimicrobial peptides, vancomycin and clindamycin^31^,^32^. D-alanylation also increases the net positive charge of the cell surface, thereby reducing the inhibition of autolysins (positively charged) by binding to the negatively charged teichoic acids^33^. Moreover, D-alanylation of teichoic acids has been related to phage resistance^34^. Interestingly, genes involved in restriction-modification, another known antiphage system, were also upregulated. The upregulation of mechanisms that give protection from bacteriophage infection may be a response of bacterial cells to the attack by the virus. In turn, it may be a competition strategy of the invading phage to prevent further infection by other viruses.

Regarding genes involved in nucleotide metabolism, most of the upregulated genes participate in purine biosynthesis (*purC, purl, purB, purH, purM, purQ*), while most of the downregulated genes are involved in the synthesis of pyrimidines (*pyrR, pyrC, pyrE, pyrF*). Interestingly, a similar trend was observed by Corrigan *et al*.^35^ in the transcriptomic analysis of *S. aureus gdpD* mutants. This mutation leads to the accumulation of c-di-AMP, whose signaling pathway exhibits cross talk with (p)ppGpp. Indeed, *gdpP* mutants show increased (p)ppGpp levels and, in turn, (p)ppGpp is known to inhibit GdpP in a dose-dependent manner^35^. Several genes involved in DNA repair were downregulated (*recU, recF, recO*) except *mutS* and *mutL*, which were induced. A similar trend was observed for genes involved in cell division and DNA replication, which were largely downregulated (*gyrA, gyrB, dinB*), although *parE*, which encodes DNA topoisomerase 4 subunit B was upregulated.

Also remarkable was the downregulation of genes encoding intracellular proteases (including *clpB, clpC, clpL* and *clpX*) and chaperones (*dnaK, dnaJ, groEL, grpE* and foldase *prsA*). In fact, *clpB* displayed the most pronounced change in gene expression, being downregulated by 112-fold. Many of these proteins are part of the heat shock response regulon and participate in refolding or degradation of misfolded or defective proteins. In contrast to *S. aureus*, infection of *E. coli* by phage PRD1 activates the heat shock regulon^6^. Nonetheless, it seems clear that stress responses are generally affected by bacteriophage infection.

Genes related to virulence and adhesion were also dysregulated. For instance, *icaA* was upregulated by 3-fold, whereas genes coding for lipase, gamma hemolysin, aureolysin, and delta hemolysin (*hld*) were downregulated. In contrast, *nuc*, encoding a thermonuclease, was induced by nearly 6-fold. In addition to *hld*, genes *agrA* and *agrC* were also downregulated. The staphyloxanthin biosynthesis operon (*crtNMQPO*) was upregulated between 2- and 5-fold. This carotenoid pigment gives certain *S. aureus* strains their characteristic golden color, and protects the microbe from the reactive oxygen species produced by the immune system. There were also changes in the expression of many genes involved in transport. In particular, a large number of genes encoding small molecule transporters were upregulated, although others were downregulated.

Exposure to phage phiIPLA-RODI had a dramatic impact on the expression of transcriptional regulators, some of which were induced (*sarZ, lytR*) and some were repressed (*ctsR, vraR, rot, sarV*). It is worth noting that *sigA* was downregulated while the stress sigma factor *sigB* was upregulated.

Dysregulation of selected genes identified in the RNA-seq analysis was confirmed by RT-qPCR (Table 2).

### Subinhibitory mupirocin increases resistance of *S. aureus* IPLA 1 to phage phiIPLA-RODI but not biofilm formation

As mentioned above, the phage-infected biofilm displayed an upregulation of the stringent response. Indeed, at least 7% of the genes identified by RNA-seq analysis belong to this regulon. For that reason, it seemed interesting to study whether this subset of genes played a role in phage resistance or increased biofilm formation. A common method to induce this response *in vitro* is exposure to the antibiotic mupirocin. To determine if the stringent response could be a defense mechanism against phage infection, we investigated the existence of synergism or antagonism between exposure to mupirocin and phage phiIPLA-RODI by the checkerboard assay. The presence of the phage at subinhibitory concentrations did not alter the mupirocin MIC (0.06 μg/ml). In contrast, low mupirocin concentrations led to a higher phage MIC. Thus, mupirocin concentrations corresponding to 1/16 × MIC led to a 10-fold increase in the MIC to phiIPLA-RODI that went from 5 × 10^4^ PFU/ml to 5 × 10^5^ PFU/ml. With higher concentrations of mupirocin (1/8, 1/4 and 1/2 of the MIC) sensitivity to the phage was reduced by 100-fold (MIC = 5 × 10^6^ PFU/ml). These results can give us some hints about the possible role of the stringent response induced by phage infection. On the one hand, it could mean that phage proliferation inside the bacterial cell triggers a response that would prevent superinfection or infection by other phages. It could also be due to the bacterium responding to prevent the virus from killing the cell. Within the context of bacteriophage therapy, it seems that treatment under conditions that minimize the activation of the stringent response may also lead to more effective biofilm elimination.

Interestingly, exposure to subinhibitory concentrations of mupirocin did not promote biofilm formation (data not shown). This suggests that upregulation of the stringent response is not the major cause of the increased adhered biomass observed in the presence of phiIPLA-RODI. Nevertheless, it cannot be ruled out that the greater expression of the major autolysin AtlA due to activation of the stringent response can have an additive effect with phage lysis and potentiate the accumulation of eDNA in the extracellular matrix. Indeed, previous reports have demonstrated the importance of autolysins in *S. epidermidis* and *S. aureus* for attachment and formation of DNA-rich biofilms^36^,^37^.

### Higher MOIs of phiIPLA-RODI can also enhance biofilm formation in more resistant strains

**Figure 6:**
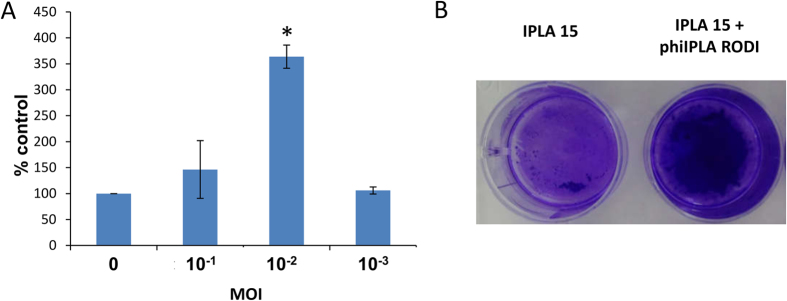
Effect of subinhibitory phage concentration on biofilm formation of strain *S. aureus* IPLA 15. (**A**) Adhered biomass of *S. aureus* IPLA 15 in the presence of different MOIs of phage phiIPLA-RODI after 24 hours of incubation at 37 °C determined by crystal violet staining and shown as percentage of the untreated control. Data correspond to the average and standard deviation of four independent biological replicates. The values obtained for each phage concentration were compared to the control grown without phage and *P*-values < 0.05 (*) were considered significant. (**B**) Photograph showing the crystal violet-stained biofilm formed by IPLA 15 in the absence (left) or presence (right) of phiIPLA-RODI at an MOI of 10^−2^.

The study of biofilms formed by *S. aureus* IPLA 1 cells exposed to a lytic phage revealed notable changes in the bacterial population. Nevertheless, it would be very interesting to test whether this phenomenon can occur in other *S. aureus* strains, in particular strains with a lesser sensitivity to phiIPLA-RODI infection. Strain *S. aureus* IPLA 15 was isolated from the meat industry and is, like IPLA 1, a medium-level biofilm former. However, this strain is 1000 times more resistant to phiIPLA-RODI, as observed by minimum inhibitory concentration (MIC) determination (MIC was 10^6^ PFU/well, which corresponds to an MOI of 1). The effect of subinhibitory phage MOIs on biofilm formation by IPLA 15 was analyzed. These experiments showed a 3.6-fold increase in biofilm formation at an MOI of 10^−2^, which is 1000× higher than the MOI leading to the maximum increase in strain IPLA1 (Fig. 6). This seems to indicate that the biofilm-promoting effect by the phage requires a certain degree of infection of the host strain.

## Discussion

It is becoming increasingly evident that understanding the dynamics of natural bacterial populations might hold the key to controlling them for our benefit. A good example is the need to overcome the growing problem of antimicrobial resistance. To do that, it is first essential to attain a greater knowledge about the physiological state of bacterial cells when they are exposed to antibiotics or disinfectants^38^,^39^. In this study, we have attempted the characterization of *S. aureus* cells in a biofilm exposed to a lytic phage. Biofilms are known to be the most common mode of bacterial growth not only in nature, but also in many man-made environments^16^,^17^. In the case of this pathogen, there are two main relevant settings, namely the clinic and the food industry, and in both biofilm formation contributes to the persistence and antimicrobial resistance of *S. aureus*^40^. The consequence of this is a greater risk for human health. Another characteristic of natural populations is their interplay with bacteriophages^3^. These viruses can infect and sometimes kill bacterial cells and constitute the most abundant “organisms” on the planet. Additionally, the utilization of phages as antimicrobial weapons is gaining attention in recent years^10^. Experience with antibiotic therapy has shown that the more we understand the effects of antimicrobials at low doses, the more we can prevent undesirable consequences or maximize their efficacy^41^. Phage therapy should be no exception. Within this context, our results could be taken both as a dissection of a real-life microbial population subject to phage predation, as much as a potential result of treatment with bacteriophages at doses that do not lead to complete eradication of the target cells. In both situations, transcriptional and physiological changes resulting from exposure to phages might affect sensitivity of the bacterial cells to antibiotics or disinfectants.

Overall, our results confirm the link between enhanced biofilm formation and lysogeny^42^,^43^,^44^ or exposure to lytic phages at concentrations that do not eradicate the population^45^,^46^. In the case of lytic phages, a study by Hosseinidoust *et al*.^45^ showed that phage predation induced biofilm formation in *P. aeruginosa, S. aureus* and *Salmonella enterica* serotype Typhimurium through undetermined non-evolutionary mechanisms. Regarding lysogenic phages, some authors have hypothesized that release of prophages could be important for the process of biofilm development in bacteria^47^. Indeed, there are many examples in which lysogeny leads to a greater biofilm formation^42^,^43^. Phage-promoted biofilms are also slightly different in composition, exhibiting a greater content in eDNA^43^,^44^. This DNA is likely a consequence of lysis by the phage, although the participation of increased autolytic activity cannot be ruled out. In that sense, the fact that phage infected biofilms of *S. aureus* showed increased expression of the gene encoding the major autolysin AtlA is very interesting. Further work should determine if this upregulation has an influence on the distinct biofilm structure displayed under low-level phage predation.

Thanks to the use of transcriptomic analyses, we have begun to understand the changes in the expression profile of cells subject to phage predation at different stages. Indeed, several recent studies have provided us with valuable information regarding gene expression of bacterial cells infected with lytic or lysogenic phages. Some examples are the articles about *P. aeruginosa*^8^,^9^, *Yersinia enterocolitica*^48^, *E. coli*^6^ or *L. lactis*^5^,^7^. However, we still had no information on *S. aureus* and, also importantly, none of these studies had attempted transcriptional profiling of surface-attached populations. Biofilm communities are notoriously complex, which makes interpretation of transcriptional or proteomic analysis more difficult. It is particularly challenging to determine which subset of the population is responsible for the observed expression changes. Albeit not a perfect system, global transcriptomic analyses of biofilm communities still provide information about general population trends in gene expression, thereby providing a good starting point to comprehend the interplay between phages and bacteria inside a biofilm. Thus, once established gene expression trends in our *in vitro* model, it would be interesting to determine if the same genes are dysregulated during phage infection of other biofilm models. For instance, subsequent experiments should study models mimicking a catheter or animal infection or models resembling the biofilms formed on food industrial surfaces.

Some of the transcriptional changes observed here are similar to those found in other phage-host models. For example, infection of *L. lactis* with the lytic phage c2 resulted in the upregulation of genes involved in D-Ala modification of teichoic acids, as well as downregulation of genes involved in metabolism, DNA replication, transcription and translation^7^. Overall, this response seemed to lead to preservation of cellular energy. D-alanylation of teichoic acids alters the structure of the cell surface and affects multiple phenotypes, including increased phage resistance and adherence to surfaces. Ainsworth *et al*.^5^ also studied transcriptional changes in *L. lactis* in response to lytic infection by phages Tuc2009 and c2. In that study, most host transcriptional changes were observed at late infection stages and were phage specific. Interestingly, infection with phage Tuc2009 induced transcription of genes involved in translation at 25 to 45 minutes post-infection, which might be linked to the high production of viral proteins at that stage. During c2 infection, there is an upregulation of amino acid biosynthesis genes and nitrogen metabolism. It does not seem that this change is due to nutrient depletion and the authors explain this phenomenon as a consequence of the intense bacteriophage protein synthesis, which may result in a temporary depletion of intracellular amino acids. Interestingly, host protein shut-off is known to occur in plant and animal cells upon viral infections^49^,^50^. In general, the responses observed in *L. lactis* as well as other species, like *E. coli*^6^ and *P. aeruginosa*^8^, involve very limited changes in gene expression, which affected mostly stress responses and occurred in late stages of the infection. In the study by Zhao *et al*.^8^ there was downregulation of ribosomal protein genes and many transcriptional regulators. Amongst the few genes upregulated several were related to cell wall metabolism. Very interestingly, the article by Poranen *et al*.^6^ on *E. coli* describes the upregulation of *spoT* at 30 minutes post infection with phage PRD1. Additionally, a recent study using a metabolomics approach detected that changes in the levels of (p)ppGpp were fairly common in the infection of *P. aeruginosa* with different phages^51^. This clearly suggests that albeit the stringent response is not always present upon phage infection, it is fairly widespread in different microbe-phage systems. In that sense, it is noteworthy that, in our model, induction of the stringent response by subinhibitory mupirocin increases resistance to phage infection. It would be very interesting to perform studies on other bacteria to assess if this is a more widespread phenomenon. Despite these similarities between the abovementioned studies and the model presented here, it must be noted that there are also notable differences. First, we have studied a biofilm model, whereas previous transcriptional analyses had been performed using liquid cultures, generally in the exponential phase. Also, we have used low-level phage pressure and allowed the host-phage system to reach equilibrium, whereas liquid culture assays were performed for relatively short incubation times and synchronized infection of the bacterial population was preferred.

Our results suggest that, under low phage predation, the *S. aureus* biofilm community can reach a certain level of infection then slow down the advance of the phage. Thus, the bacterial population can obtain the benefits of a more stable thicker biofilm, which is protective against other external sources of stress. At the same time, activation of the stringent response would keep the proliferation of the phage under control to avoid complete eradication of the bacteria. Although this may seem counterintuitive, this situation would also have benefits for the bacteriophage as the biofilm would be a continuous reservoir of new virions and sensitive host cells. Indeed, extermination of the host would ultimately lead to disappearance of the predator. Therefore, under the conditions of the experiment, interactions between prey and predator would reach an equilibrium that is advantageous to both. Nevertheless, the specific details of this equilibrium still need to be determined in subsequent studies. For instance, it will be imperative to discern which of the observed changes correspond to non-infected cells and which are displayed by cells infected by the bacteriophage. Also, it is necessary to dissect which of the identified genes bear relevance towards the biofilm increase observed, as well as towards phage sensitivity. To do that, subsequent studies should analyse the effects of lack or overexpression of selected genes whose expression changed in response to phage predation. Here, we have identified a potential link between the stringent response and phage resistance. Nonetheless, this phenomenon requires further attention to determine the molecular mechanisms involved.

The information presented in this study can be very useful to develop new products that minimize the occurrence of undesired effects upon application of phage-containing antimicrobials against *S. aureus*. For instance, our results identified the main responses of *S. aureus* biofilm cells exposed to phage predation. Subsequent studies should aim to determine whether inhibition of these responses may enhance the use of phages as antimicrobials. Furthermore, antibiotics and disinfectants may be improved by taking into consideration the effect of bacteriophages in the target populations. As mentioned previously, natural populations might exhibit some similarities to our *in vitro* biofilm model. More specifically, biofilm communities in nature are likely subject to phage predation but not generally at a level that would lead to complete eradication of the bacterial population. Therefore, the trascriptome of such communities might share some trends with the ones observed here. With this in mind, it would be interesting to determine if biofilms developed under low-level phage predation exhibit different sensitivity to commonly used antibiotics or disinfectants. Ultimately, when it comes to fighting bacteria, knowledge is our best weapon.

## Methods

### Bacterial strains, bacteriophages and culture conditions

Two different *S. aureus* strains were used for this study, namely IPLA 1 and IPLA 15, which had been respectively isolated from samples taken in the dairy and meat industry^52^. *S. aureus* cultures were routinely grown in TSB (Tryptic Soy Broth, Scharlau, Barcelona, Spain) at 37 °C with shaking or on Baird-Parker agar plates (AppliChem, Germany). The lytic bacteriophage phiIPLA-RODI^21^ was propagated on *S. aureus* IPLA 1 as previously described^21^. Mupirocin was purchased from Panreac Quimica SLU (Spain).

### Biofilm formation assays

Biofilms were grown in 12-well microtiter plates (Thermo Scientific, NUNC, Madrid, Spain) according to the method described by Herrera *et al*.^53^ with some modifications. Briefly, overnight cultures of *S. aureus* were diluted in TSBg (TSB supplemented with 0.25% w/v D-( + )-glucose) to obtain a cell suspension of 10^6^ cfu/ml. 1 ml aliquots of this suspension were used to inoculate each well and then 1 ml of phage suspensions at different titers were added. 1 ml of TSBg was added to the control well. These microtiter plates were then incubated for 3, 5, 7 or 24 hours at 37 °C.

Following incubation during the desired time, the planktonic phase was removed and analyzed for OD_600_ and viable cell counts. The adhered phase was washed twice with phosphate-buffered saline (PBS) buffer (137 mM NaCl, 2.7 mM KCl, 10 mM Na_2_HPO_4_ and 2 mM KH_2_PO_4_; pH 7.4) and subsequently stained with crystal violet or scraped to determine viable cell counts.

Crystal violet staining was performed by adding 2 ml of 0.1% (w/v) crystal violet to each well. Following 15 minutes of incubation, the excess dye was removed by washing twice with water. The crystal violet attached to the well was distained with 2 ml of a 33% (v/v) solution of acetic acid and absorbance at 595 nm was quantified with a Bio-Rad Benchmark plus microplate spectrophotometer (Bio-Rad Laboratories, Hercules, CA, USA).

In order to count the adhered cells, each well was washed with PBS and subsequently scraped twice with sterile cotton swabs. Then these cells were resuspended by vigorously vortexing for 1 min as described previously^54^. Serial dilutions of the resulting suspension were then plated on TSA and incubated at 37 °C.

The minimum inhibitory MOI was considered to be the one in which no visible growth could be observed in the well after 24 hours of incubation at 37 °C. The minimum bactericidal MOI was determined to be the one in which no viable cells could be recovered following incubation for 24 hours.

### Confocal microscopy

24 hour-old biofilms were formed on 2-well μ-slides with a glass bottom (ibidi, USA) in the presence of phage phiIPLA-RODI or SM (negative control). After removing the planktonic phase, wells were washed with PBS and stained with Live/Dead^®^ BacLight^TM^ kit (Invitrogen AG, Basel, Switzerland). Samples were observed with a confocal scanning laser microscope (DMi8, Leica Microsystems) using a 100 × oil objective.

### Anaysis of eDNA composition of biofilms

Recovery and visualization of eDNA from *S. aureus* biofilms was performed as described by Kaplan *et al*.^55^. Briefly, biofilms were formed in 12-well microtiter plates. After 24 hours, the planktonic phase was removed and the biofilm was washed once with PBS. 1 ml of TE buffer (10 mM Tris, 1 mM EDTA [pH 8]) was added and the adhered cells were scraped from the bottom of the well with a pipette tip. The cell suspension was transferred to a 1.5-ml tube and centrifuged at 13,000 rpm for 30 s. The supernatant was discarded and the pellets were resuspended in 200 μl of TE. After a second centrifugation step, 20 μl of the supernatant were loaded into a 1% agarose gel and subsequently stained with ethidium bromide.

Additionally, to compare the eDNA content of different biofilms, DNAse treatment was performed as previously described with some modifications^54^. Briefly, biofilms were preformed for 24 hours in 12-well microtiter plates. The planktonic phase was removed and the adhered cells were washed once with PBS. Then, 1 ml of 200 μg/ml DNAse in activity buffer (150 mM NaCl and 1 mM CaCl_2_) or buffer alone were added to the wells. The microtiter plate was incubated for 1 hour at 37 °C. After that, the liquid was removed and biofilms were stained with crystal violet.

### RNA purification

In order to perform the transcriptomic analysis, total RNA was isolated from *S. aureus* IPLA 1 biofilms grown with or without addition of bacteriophage at an MOI of 10^−5^. Following 24 hours of incubation at 37 °C, the supernatant was removed and the adhered cells were washed with PBS and subsequently scraped with a 1-ml pipette tip in a solution containing 1 ml RNA protect (Qiagen) and 0.5 ml PBS. Cells were then incubated at room temperature for 5 minutes, pelleted at 5,000 × g for 10 min and stored at −80 °C until further processing. Samples were thawed and cells were lysed by mechanical disruption with a FastPrep^®^-24 in a solution of phenol-chloroform 1:1, glass beads (Sigma) and 80 mM DTT. RNA was isolated using the Illustra RNA spin Mini kit (GE Healthcare) and treated with Turbo DNAse (Ambion) to remove traces of genomic DNA. For storage, 1 μl Superase inhibitor (Ambion) was added to 50 μl of sample. RNA concentration was measured by using a microplate spectrophotometer Epoch (Biotek). RNA quality was checked by agarose gel electrophoresis of the samples.

### RNA-seq and RT-qPCR

A total of 8 μg of RNA from each sample were sent to Macrogen Inc. (South Korea) for sequencing using the Illumina HiSeq2000 platform (Illumina, San Diego, CA, USA). Bioinformatic analysis was performed at Dreamgenics (Dreamgenics, Oviedo, Spain). Quality control of the reads was performed with FastQC. RNA-seq reads were mapped to the *S. aureus* NCTC 8325 and phage phiIPLA-RODI genomes by using BowTie2. Only the uniquely mapped reads were kept for the subsequent analyses. Differential gene expression analysis was performed using EDGE-pro software. Quantitative reverse transcription-PCR (RT-qPCR) was performed to verify transcriptional changes for selected differentially-expressed genes identified in the RNA-seq analysis. Briefly, 0.5 μg of purified RNA were converted into cDNA with iScript™ Reverse Transcription Supermix for RT-qPCR (BioRad). The resulting cDNA was then diluted 1:25 and 2.5 μl were added to each well together with Power SYBR Green PCR Master Mix (Applied BioSystems) for qPCR analysis.

RNA-Seq data have been deposited in NCBI’s Gene Expression Omnibus (GEO) and can be accessed through GEO series accession number GSE87706.

### Assessment of interactions between mupirocin and phiIPLA-RODI

The technique used to test whether there were synergistic or antagonistic interactions between the phage phiIPLA-RODI and the antibiotic mupirocin was the checkerboard assay^56^. This method is based on the broth microdilution technique, in which two different antimicrobials are diluted in a two-dimensional fashion. Thus, each well of a 96-well microtiter plate contained a unique combination of mupirocin concentration and MOI. Broth microdilution was performed following the CLSI guidelines^57^,^58^ but using TSBg as a growth medium. The minimum inhibitory concentration (MIC) for each antimicrobial was determined as the lowest concentration inhibiting visible bacterial growth after 24 hours of incubation at 37 °C. The experiment was performed with four independent biological repeats.

### Statistical analyses

All experiments were performed with at least three independent biological replicates and on a minimum of two different days. Data were analyzed with a two-tailed Student’s t-test by using IBM SPSS Statistics for Windows, Version 22.0 (IBN Corp. Armonk, NY). *P*-values < 0.05 were considered significant.

## Additional Information

**How to cite this article**: Fernández, L. *et al*. Low-level predation by lytic phage phiIPLA-RODI promotes biofilm formation and triggers the stringent response in *Staphylococcus aureus. Sci. Rep.* **7**, 40965; doi: 10.1038/srep40965 (2017).

**Publisher's note:** Springer Nature remains neutral with regard to jurisdictional claims in published maps and institutional affiliations.

## Supplementary Material

Supplementary Video

Supplementary Tables

## Figures and Tables

**Table 1:** List of genes related to the stringent response that are dysregulated in biofilms treated with subinhibitory doses of phiIPLA-RODI compared to untreated biofilms according to RNA-seq.

Gene ID	Gene name	Gene product	FC
SAOUHSC_00005	*gyrB*	DNA gyrase subunit B	−2.89
SAOUHSC_00006	*gyrA*	DNA gyrase subunit A	−3.92
SAOUHSC_00121		Capsular polysaccharide synthesis enzyme O-acetyl transferase Cap5H, putative	2.03
SAOUHSC_00187	*pflB*	Formate acetyltransferase	−4.57
SAOUHSC_00188	*pflA*	Pyruvate formate-lyase-activating enzyme	−5.52
SAOUHSC_00350	*rpsR*	30 S ribosomal protein S18	−2.69
SAOUHSC_00471	*glmU*	Bifunctional protein GlmU	−2.73
SAOUHSC_00474	*rplY*	50 S ribosomal protein L25	−2.06
SAOUHSC_00528	*rpsG*	30 S ribosomal protein S7	−2.07
SAOUHSC_00733	*hisC*	Histidinol-phosphate aminotransferase	3.53
SAOUHSC_00780	*uvrA*	UvrABC system protein A	−2.07
SAOUHSC_00796	*pgk*	Phosphoglycerate kinase	−2.90
SAOUHSC_00797	*tpiA*	Triosephosphate isomerase	−3.32
SAOUHSC_00799	*eno*	Enolase	−4.31
SAOUHSC_00802		Carboxylesterase, putative	−2.11
SAOUHSC_00818	*nuc*	Thermonuclease	5.72
SAOUHSC_00933	*trpS*	Tryptophan–tRNA ligase	2.25
SAOUHSC_00994	*atl*	Bifunctional autolysin	5.42
SAOUHSC_01002	*qoxA*	Probable quinol oxidase subunit 2	−2.54
SAOUHSC_01092	*pheS*	Phenylalanine–tRNA ligase alpha subunit	−2.04
SAOUHSC_01093	*pheT*	Phenylalanine–tRNA ligase beta subunit	−3.45
SAOUHSC_01164	*pyrR*	Bifunctional protein PyrR	−2.25
SAOUHSC_01168	*pyrC*	Dihydroorotase	−4.79
SAOUHSC_01207	*ffh*	Signal recognition particle protein	−2.50
SAOUHSC_01216	*sucC*	Succinyl-CoA ligase [ADP-forming] subunit beta	−2.80
SAOUHSC_01247	*rbfA*	Ribosome-binding factor A	−4.19
SAOUHSC_01276	*glpK*	Glycerol kinase	−2.56
SAOUHSC_01320	*hom*	Homoserine dehydrogenase	3.25
SAOUHSC_01395	*asd*	Aspartate-semialdehyde dehydrogenase	4.35
SAOUHSC_01396	*dapA*	4-hydroxy-tetrahydrodipicolinate synthase	4.40
SAOUHSC_01397	*dapB*	4-hydroxy-tetrahydrodipicolinate reductase	2.81
SAOUHSC_01466	*recU*	Holliday junction resolvase RecU	−3.02
SAOUHSC_01504		Ferredoxin, putative	−3.37
SAOUHSC_01585	*srrB*	Sensor protein SrrB	−3.29
SAOUHSC_01586	*srrA*	Transcriptional regulatory protein SrrA	−5.06
SAOUHSC_01601		Alpha-glucosidase, putative	−2.76
SAOUHSC_01668	*era*	GTPase Era	−2.96
SAOUHSC_01681	*prmA*	Ribosomal protein L11 methyltransferase	−13.21
SAOUHSC_01715	*udk*	Uridine kinase	−2.38
SAOUHSC_01737	*aspS*	Aspartate–tRNA ligase	−2.95
SAOUHSC_01742	*rsh*	GTP pyrophosphokinase	4.91
SAOUHSC_01755	*rpmA*	50 S ribosomal protein L27	−3.58
SAOUHSC_01767	*valS*	Valine–tRNA ligase	−3.46
SAOUHSC_01771	*hemL1*	Glutamate-1-semialdehyde 2,1-aminomutase 1	−2.90
SAOUHSC_01772	*hemB*	Delta-aminolevulinic acid dehydratase	−2.71
SAOUHSC_01776	*hemA*	Glutamyl-tRNA reductase	−2.06
SAOUHSC_01784	*rplT*	50 S ribosomal protein L20	−2.31
SAOUHSC_01818	*ald2*	Alanine dehydrogenase 2	−2.89
SAOUHSC_01886	*ribH*	6,7-dimethyl-8-ribityllumazine synthase	−10.04
SAOUHSC_01887	*ribBA*	Riboflavin biosynthesis protein	−7.35
SAOUHSC_01889	*ribD*	Riboflavin biosynthesis protein	−4.03
SAOUHSC_01961	*hemH*	Ferrochelatase	−2.32
SAOUHSC_01972	*prsA*	Foldase protein PrsA	−3.26
SAOUHSC_02254	*groEL*	60 kDa chaperonin	−4.48
SAOUHSC_02340	*atpC*	ATP synthase epsilon chain	−2.87
SAOUHSC_02341	*atpD*	ATP synthase subunit beta	−2.87
SAOUHSC_02349	*atpE*	ATP synthase subunit c	−2.38
SAOUHSC_02350	*atpB*	ATP synthase subunit a	−2.31
SAOUHSC_02477	*rpsI*	30 S ribosomal protein S9	−2.08
SAOUHSC_02505	*rplP*	50 S ribosomal protein L16	−2.02
SAOUHSC_02506	*rpsC*	30 S ribosomal protein S3	−2.11
SAOUHSC_02509	*rplB*	50 S ribosomal protein L2	−2.72
SAOUHSC_02510	*rplW*	50 S ribosomal protein L23	−2.71
SAOUHSC_02511	*rplD*	50 S ribosomal protein L4	−2.65
SAOUHSC_02536	*moaA*	Cyclic pyranopterin monophosphate synthase	−2.81
SAOUHSC_02537	*mobA*	Probable molybdenum cofactor guanylyltransferase	−3.16
SAOUHSC_02635	*tcaA*	Membrane-associated protein TcaA	−2.32
SAOUHSC_02669	*sarZ*	HTH-type transcriptional regulator SarZ	4.75
SAOUHSC_02696	*fmhA*	FmhA protein	2.68
SAOUHSC_02834	*srtA*	Sortase	2.95
SAOUHSC_02850	*cidB*	Holin-like protein CidB	−2.28
SAOUHSC_02965	*arcC2*	Carbamate kinase 2	−6.75
SAOUHSC_02969	*arcA*	Arginine deiminase	−5.89
SAOUHSC_03002	*zwf*	Poly-beta-1,6-N-acetyl-D-glucosamine synthase	3.16
SAOUHSC_03002	*icaA*	Poly-beta-1,6-N-acetyl-D-glucosamine synthase	3.16
SAOUHSC_03055	*rpmH*	50 S ribosomal protein L34	−3.07

**Table 2:** Confirmation by RT-qPCR of the dysregulation for selected genes identified by RNA-seq analysis.

Gene	Gene product	FC (RT-qPCR)	FC (RNA-seq)
*atl*	Bifunctional autolysin	31.92 ± 18.84	5.42
*rsh*	GTP pyrophosphokinase	33.90 ± 17.22	4.91
*relP*	GTP pyrophosphokinase	23.54 ± 8.95	2.21
*clpC*	ATP-dependent Clp protease, ATP-binding subunit	−4.96 ± 0.99	−22.97
*clpB*	ATP-dependent Clp protease, ATP-binding subunit	−28.03 ± 9.39	−112.83
*crtP*	Diapolycopene oxygenase	25.59 ± 14.78	3.34
*dltA*	D-alanine—poly(phosphoribitol) ligase subunit 1	20.21 ± 11.85	3.69
*sigB*	RNA polymerase sigma factor	16.13 ± 5.73	2.56
*nuc*	Thermonuclease	19.20 ± 11.24	5.72
*icaA*	Poly-beta-1,6-N-acetyl-D-glucosamine synthase	4.06 ± 0.76	3.16
