# Supplementary material for: Low-level predation by lytic phage phiIPLA-RODI promotes biofilm formation and triggers the stringent response in Staphylococcus aureus
Source: Sci Rep. 2017 Jan 19;7:40965. doi: 10.1038/srep40965 (PMC5244418; doi:10.1038/srep40965)
Supplement: Supplementary Tables [file srep40965-s2.pdf]

Low-level predation by lytic phage phiIPLA-RODI promotes biofilm formation and triggers the stringent response in *Staphylococcus aureus*

Running title: *Staphylococcus* biofilms under low phage predation

Lucía Fernández*, Silvia González, Ana Belén Campelo, Beatriz Martínez, Ana Rodríguez, Pilar García

Address: Instituto de Productos Lácteos de Asturias (IPLA-CSIC). Paseo Río Linares s/n 33300- Villaviciosa, Asturias, Spain.

***Corresponding author:** Lucía Fernández
IPLA-CSIC, 33300-Villaviciosa, Asturias, Spain.

e-mail: lucia.fernandez@ipla.csic.es
Phone: +34 985 89 21 31
Fax: +34 985 89 22 33

Table S1. Normalized mean reads per kilobase million (RPKM) values corresponding to the different open reading frames (ORFs) of phiIPLA-RODI genome in the treated samples

gene_ID	start_coord	end_coord	RODI-1 Reads	RODI-2 Reads	RODI-3 Reads	RODI-1 RPKM	RODI-2 RPKM	RODI-3 RPKM	RPKM MEAN	RPKM STD	gene_product_acce	gene_name
AVU41_gp001	14	496	7265	4771	7648	3210	2128	3504	**2947.333333**	724.6304805	YP_009195837.1	terminal repeat-encoded protein
AVU41_gp002	575	739	3937	2829	3479	5092	3694	4665	**4483.666667**	716.423292	YP_009195838.1	terminal repeat-encoded protein
AVU41_gp003	739	1008	3190	2073	3133	2522	1654	2568	**2248**	514.9330054	YP_009195839.1	TreT
AVU41_gp004	1093	1314	2503	1646	2382	2406	1597	2374	**2125.666667**	458.1182526	YP_009195840.1	TreU
AVU41_gp005	1642	1932	1046	970	1200	767	718	912	**799**	100.8811182	YP_009195841.1	hypothetical protein
AVU41_gp006	2036	2272	1823	1778	2467	1642	1616	2303	**1853.666667**	389.3511697	YP_009195842.1	BofL
AVU41_gp007	2274	2759	5572	5262	6959	2447	2332	3168	**2649**	453.1302241	YP_009195843.1	hypothetical protein
AVU41_gp008	2772	3179	3516	3108	4572	1839	1641	2480	**1986.666667**	438.559384	YP_009195844.1	hypothetical protein
AVU41_gp009	3179	3610	9576	8941	11293	4731	4459	5784	**4991.333333**	699.8116413	YP_009195845.1	UboA
AVU41_gp010	3613	3804	3336	3022	3763	3708	3391	4337	**3812**	481.498702	YP_009195846.1	hypothetical protein
AVU41_gp011	3801	4286	3305	3091	4100	1451	1370	1867	**1562.666667**	266.653958	YP_009195847.1	membrane protein
AVU41_gp012	4279	4710	2428	2089	2732	1200	1042	1399	**1213.666667**	178.8919599	YP_009195848.1	hypothetical protein
AVU41_gp013	4724	5266	3328	3414	4129	1308	1354	1683	**1448.333333**	204.5246521	YP_009195849.1	nucleotidyl transferase
AVU41_gp014	5278	5766	6345	6231	8380	2769	2745	3792	**3102**	597.678007	YP_009195850.1	hypothetical protein
AVU41_gp015	5779	6177	3378	3210	4436	1807	1733	2460	**2000**	400.0862407	YP_009195851.1	hypothetical protein
AVU41_gp016	6174	6881	6144	6251	8004	1852	1902	2501	**2085**	361.1329395	YP_009195852.1	phosphatase
AVU41_gp017	6981	7535	6037	5482	5303	2322	2128	2114	**2188**	116.258333	YP_009195853.1	hypothetical protein
AVU41_gp018	7551	7868	7066	6899	6048	4742	4674	4208	**4541.333333**	290.6704893	YP_009195854.1	hypothetical protein
AVU41_gp019	8854	9402	2185	1898	2869	849	745	1156	**916.6666667**	213.6921462	YP_009195855.1	hypothetical protein
AVU41_gp020	9406	9624	2219	1802	2772	2163	1773	2801	**2245.666667**	518.9617841	YP_009195856.1	hypothetical protein
AVU41_gp021	9625	9819	1871	1570	2425	2048	1734	2752	**2178**	521.3022156	YP_009195857.1	hypothetical protein
AVU41_gp022	9809	10546	4348	3530	5848	1257	1030	1753	**1346.666667**	369.7463094	YP_009195858.1	hypothetical protein
AVU41_gp023	10609	10713	335	292	463	681	599	976	**752**	198.2750615	YP_009195859.1	hypothetical protein
AVU41_gp024	10735	10962	1610	1501	1905	1507	1418	1849	**1591.333333**	227.5397401	YP_009195860.1	aspartate aminotransferase
AVU41_gp025	10964	11350	2175	1928	2765	1199	1073	1581	**1284.333333**	264.5322917	YP_009195861.1	hypothetical protein
AVU41_gp026	11447	11620	1724	1641	1644	2115	2032	2091	**2079.333333**	42.71221527	YP_009195862.1	hypothetical protein
AVU41_gp027	11661	12143	13837	13526	12982	6114	6033	5947	**6031.333333**	83.51247412	YP_009195863.1	hypothetical protein
AVU41_gp028	12193	12735	21313	21377	27265	8377	8481	11110	**9322.666667**	1548.74928	YP_009195864.1	hypothetical protein
AVU41_gp029	12735	13265	23677	23393	29378	9516	9490	12242	**10416**	1581.415821	YP_009195865.1	hypothetical protein
AVU41_gp030	13268	13432	4071	4012	4881	5266	5238	6546	**5683.333333**	747.2224122	YP_009195866.1	membrane protein
AVU41_gp031	13435	13722	2278	1776	3181	1688	1328	2444	**1820**	569.5893257	YP_009195867.1	membrane protein
AVU41_gp032	13722	14567	4783	3788	6867	1207	965	1796	**1322.666667**	427.4041803	YP_009195868.1	hypothetical protein
AVU41_gp033	14580	15698	8045	6581	12094	1534	1267	2391	**1730.666667**	587.2412906	YP_009195869.1	AAA family ATPase
AVU41_gp034	15851	16177	614	656	765	401	432	518	**450.3333333**	60.61627944	YP_009195870.1	hypothetical protein
AVU41_gp035	16170	16586	1544	1932	1989	790	998	1055	**947.6666667**	139.4859611	YP_009195871.1	hypothetical protein
AVU41_gp036	16718	17020	5337	5998	7994	3759	4264	5838	**4620.333333**	1084.338662	YP_009195872.1	NTP-PPase
AVU41_gp037	17020	17208	3201	3761	4758	3615	4287	5570	**4490.666667**	993.2856253	YP_009195873.1	hypothetical protein
AVU41_gp038	17252	17413	1350	1629	2048	1779	2166	2797	**2247.333333**	513.8504971	YP_009195874.1	hypothetical protein
AVU41_gp039	17413	19464	9764	10668	15555	1016	1120	1677	**1271**	355.4307246	YP_009195875.1	hypothetical protein
AVU41_gp040	19543	19806	2595	2249	2986	2098	1835	2503	**2145.333333**	336.5060673	YP_009195876.1	hypothetical protein
AVU41_gp041	19823	19996	6780	6140	7010	8316	7602	8914	**8277.333333**	656.8541188	YP_009195877.1	peptidoglycan binding protein
AVU41_gp042	20003	20581	19312	17405	19132	7119	6476	7312	**6969**	437.7202303	YP_009195878.1	hypothetical protein
AVU41_gp043	20574	21173	9978	7850	12773	3549	2818	4711	**3692.666667**	954.642516	YP_009195879.1	nucleoside 2-deoxyribosyltransferase
AVU41_gp044	21166	22059	6664	5609	7706	1591	1352	1907	**1616.666667**	278.3888168	YP_009195880.1	RNA ligase
AVU41_gp045	22063	22287	253	255	304	240	244	299	**261**	32.96968304	YP_009195881.1	hypothetical protein
AVU41_gp046	22356	23096	25456	23540	34928	7332	6844	10430	**8202**	1944.871204	YP_009195882.1	PhoH-related protein
AVU41_gp047	23148	23762	27662	26138	40088	9600	9156	14423	**11059.66667**	2921.179956	YP_009195883.1	hypothetical protein
AVU41_gp048	23778	24203	10524	10653	15774	5272	5387	8193	**6284**	1654.242123	YP_009195884.1	ribonuclease
AVU41_gp049	24193	24384	5604	5524	8335	6229	6198	9606	**7344.333333**	1958.722117	YP_009195885.1	hypothetical protein
AVU41_gp050	24407	25048	8752	9321	13661	2909	3128	4708	**3581.666667**	981.5601527	YP_009195886.1	hypothetical protein
AVU41_gp051	25038	25268	4584	4822	7272	4235	4497	6966	**5232.666667**	1506.815959	YP_009195887.1	DNA binding protein
AVU41_gp052	25271	25498	3861	4178	5144	3614	3948	4992	**4184.666667**	718.8388786	YP_009195888.1	hypothetical protein

AVU41_gp053	25607	26299	1469	1155	635	452	359	203	**338**	125.8213019	YP_009195889.1	transglycosylase
AVU41_gp054	26497	27291	6536	5291	10391	1755	1434	2892	**2027**	766.112916	YP_009195890.1	membrane protein
AVU41_gp055	27291	27599	1943	1822	3039	1342	1270	2176	**1596**	503.583161	YP_009195891.1	hypothetical protein
AVU41_gp056	27712	28332	7312	8527	9061	2513	2958	3229	**2900**	361.5065698	YP_009195892.1	hypothetical protein
AVU41_gp057	28395	29885	133411	112691	101786	19097	16282	15106	**16828.33333**	2050.824306	YP_009195893.1	endolysin
AVU41_gp058	29885	30388	49610	42239	35054	21008	18054	15390	**18150.66667**	2810.247201	YP_009195894.1	holin
AVU41_gp059	30473	30658	1312	1003	761	1505	1162	905	**1190.666667**	301.0254696	YP_009195895.1	hypothetical protein
AVU41_gp060	32211	32429	280	292	223	273	287	225	**261.6666667**	32.5166624	YP_009195896.1	hypothetical protein
AVU41_gp061	32909	33118	1097	1000	727	1115	1026	766	**969**	181.3477323	YP_009195897.1	hypothetical protein
AVU41_gp062	33131	33463	14729	13806	8734	9440	8931	5804	**8058.333333**	1968.828162	YP_009195898.1	hypothetical protein
AVU41_gp063	33476	33802	10441	9343	6250	6815	6155	4229	**5733**	1343.656206	YP_009195899.1	membrane protein
AVU41_gp064	34243	34629	17048	19193	12013	9402	10684	6869	**8985**	1941.3843	YP_009195900.1	membrane protein
AVU41_gp065	34607	34885	15366	17116	10999	11754	13216	8723	**11231**	2291.704388	YP_009195901.1	hypothetical protein
AVU41_gp066	34882	35292	27305	31601	22802	14179	16564	12276	**14339.66667**	2148.510259	YP_009195902.1	hypothetical protein
AVU41_gp067	35307	35657	38490	43395	34365	23404	26634	21664	**23900.66667**	2521.950304	YP_009195903.1	terminase large subunit
AVU41_gp068	35897	36667	39613	42217	32044	10965	11796	9196	**10652.33333**	1327.900724	YP_009195904.1	VSR homing endonuclease
AVU41_gp069	36734	38194	117317	132310	113521	17138	19509	17193	**17946.66667**	1353.299794	YP_009195905.1	terminase large subunit
AVU41_gp070	38187	39008	94010	107990	93952	24409	28301	25291	**26000.33333**	2040.657084	YP_009195906.1	hypothetical protein
AVU41_gp071	39165	39644	47206	53379	47202	20989	23957	21759	**22235**	1540.190897	YP_009195907.1	hypothetical protein
AVU41_gp072	39686	40936	185309	209017	217667	31614	35993	38500	**35369**	3485.151503	YP_009195908.1	membrane protein
AVU41_gp073	41021	41362	3965	4162	3090	2474	2622	1999	**2365**	325.4888631	YP_009195909.1	membrane protein
AVU41_gp074	41372	41752	11515	11811	7605	6450	6678	4417	**5848.333333**	1244.802126	YP_009195910.1	hypothetical protein
AVU41_gp075	41756	43447	85790	90354	73991	10821	11504	9676	**10667**	923.6790568	YP_009195911.1	portal protein
AVU41_gp076	43641	44414	85103	75376	50044	23466	20979	14307	**19584**	4736.173455	YP_009195912.1	prohead protease
AVU41_gp077	44433	45383	179706	168734	116254	40330	38222	27049	**35200.33333**	7137.513036	YP_009195913.1	hypothetical protein
AVU41_gp078	45499	46890	400189	355493	289563	61358	55016	46029	**54134.33333**	7702.438726	YP_009195914.1	capsid protein
AVU41_gp079	46982	47278	15120	15188	9738	10865	11016	7255	**9712**	2129.163451	YP_009195915.1	hypothetical protein
AVU41_gp080	47291	48199	35073	33918	27137	8235	8038	6606	**7626.333333**	889.107605	YP_009195916.1	hypothetical protein
AVU41_gp081	48213	49091	29854	30190	24121	7249	7399	6072	**6906.666667**	726.72301	YP_009195917.1	hypothetical protein
AVU41_gp082	49091	49711	31388	31848	24688	10787	11048	8797	**10210.66667**	1231.20686	YP_009195918.1	hypothetical protein
AVU41_gp083	49730	50566	84852	82994	56840	21636	21361	15026	**19341**	3739.428432	YP_009195919.1	hypothetical protein
AVU41_gp084	50568	50783	20270	18872	13855	20028	18822	14193	**17681**	3080.294953	YP_009195920.1	hypothetical protein
AVU41_gp085	50810	52573	357465	306226	250417	43249	37397	31412	**37352.66667**	5918.624531	YP_009195921.1	major tail sheath protein
AVU41_gp086	52646	52984	59237	50340	41201	37294	31990	26893	**32059**	5200.843297	YP_009195922.1	tail tube
AVU41_gp087	53779	54783	12733	13501	11690	2704	2894	2574	**2724**	160.9347694	YP_009195923.1	Ioh
AVU41_gp088	54840	54980	990	1115	1015	1499	1704	1593	**1598.666667**	102.6174124	YP_009195924.1	hypothetical protein
AVU41_gp089	55023	55481	1450	1502	1196	674	705	577	**652**	66.7757441	YP_009195925.1	hypothetical protein
AVU41_gp090	55494	55688	238	241	296	260	266	336	**287.3333333**	42.25320501	YP_009195926.1	hypothetical protein
AVU41_gp091	55770	56081	2581	1886	3034	1766	1302	2152	**1740**	425.5960526	YP_009195927.1	hypothetical protein
AVU41_gp092	56213	56671	17573	17600	10456	8171	8260	5041	**7157.333333**	1833.338576	YP_009195928.1	hypothetical protein
AVU41_gp093	56715	57251	37759	36595	27545	15007	14681	11350	**13679.33333**	2023.836538	YP_009195929.1	tail morphogenetic protein
AVU41_gp094	57307	61362	115298	114435	93488	6067	6078	5100	**5748.333333**	561.5000742	YP_009195930.1	TMP protein
AVU41_gp095	61441	63867	23300	23989	21662	2049	2129	1975	**2051**	77.01947806	YP_009195931.1	CHAP domain protein
AVU41_gp096	63881	64768	11986	13188	12693	2881	3199	3163	**3081**	174.1378764	YP_009195932.1	protease
AVU41_gp097	64768	67314	59937	61044	66555	5022	5163	5782	**5322.333333**	404.2775449	YP_009195933.1	glycerophosphoryl diester phosphatase
AVU41_gp098	67421	68212	22376	25359	27205	6030	6898	7601	**6843**	786.9428188	YP_009195934.1	hypothetical protein
AVU41_gp099	68212	68736	14306	17270	18795	5816	7086	7922	**6941.333333**	1060.426958	YP_009195935.1	hypothetical protein
AVU41_gp100	68736	69440	27111	31742	33870	8207	9699	10630	**9512**	1222.276155	YP_009195936.1	baseplate protein
AVU41_gp101	69455	70501	47274	55072	56453	9636	11331	11931	**10966**	1190.241572	YP_009195937.1	baseplate protein
AVU41_gp102	70522	73587	108767	122687	131758	7571	8620	9509	**8566.666667**	970.1001666	YP_009195938.1	hypothetical protein
AVU41_gp103	73698	74219	43363	48259	49367	17729	19916	20926	**19523.66667**	1634.211227	YP_009195939.1	baseplate protein
AVU41_gp104	74240	77698	427519	413149	519699	26378	25731	33245	**28451.33333**	4164.022374	YP_009195940.1	adsorption-associated tail protein
AVU41_gp105	77747	77905	14311	14889	15019	19210	20173	20901	**20094.66667**	848.2171499	YP_009195941.1	hypothetical protein
AVU41_gp106	77906	79825	147215	146940	169589	16364	16487	19544	**17465**	1801.516861	YP_009195942.1	carbohydrate binding domain protein

AVU41_gp107	79847	80218	41204	41929	46099	23640	24281	27420	**25113.66667**	2022.894049	YP_009195943.1	hypothetical protein
AVU41_gp108	80225	81601	129068	118055	158599	20005	18469	25485	**21319.66667**	3688.133042	YP_009195944.1	hypothetical protein
AVU41_gp109	81691	83439	104968	126857	104460	12809	15625	13216	**13883.33333**	1521.993539	YP_009195945.1	DNA helicase
AVU41_gp110	83451	85064	88245	107746	91658	11669	14381	12566	**12872**	1381.652272	YP_009195946.1	Rep protein
AVU41_gp111	85057	86499	126228	154229	138061	18670	23025	21170	**20955**	2185.446179	YP_009195947.1	ATPase
AVU41_gp112	86578	86997	3541	3255	3802	1799	1670	2003	**1824**	167.901757	YP_009195948.1	hypothetical protein
AVU41_gp113	86997	88022	15407	11488	20011	3205	2412	4316	**3311**	956.4157046	YP_009195949.1	exonuclease
AVU41_gp114	88022	88399	11285	8773	13360	6372	5000	7821	**6397.666667**	1410.675134	YP_009195950.1	hypothetical protein
AVU41_gp115	88399	90318	40434	33423	47864	4495	3750	5516	**4587**	886.5872771	YP_009195951.1	ATPase
AVU41_gp116	90318	90914	10093	8169	12113	3608	2948	4490	**3682**	773.6588395	YP_009195952.1	hypothetical protein
AVU41_gp117	90929	91996	13291	10508	15639	2656	2120	3240	**2672**	560.1714023	YP_009195953.1	DNA primase
AVU41_gp118	92062	92400	20634	20095	19622	12991	12770	12808	**12856.33333**	118.1623177	YP_009195954.1	hypothetical protein
AVU41_gp119	92400	92852	49783	48713	49343	23455	23166	24102	**23574.33333**	479.2747994	YP_009195955.1	hypothetical protein
AVU41_gp120	92839	93447	42706	41351	46882	14966	14627	17034	**15542.33333**	1302.89383	YP_009195956.1	resolvase
AVU41_gp121	93437	93856	20775	20403	16505	10557	10465	8695	**9905.666667**	1049.476695	YP_009195957.1	ribonucleotide reductase flavodoxin
AVU41_gp122	93871	95985	113256	109276	96148	11429	11130	10059	**10872.66667**	720.3404288	YP_009195958.1	ribonucleotide reductase large subunit
AVU41_gp123	95999	97048	56205	55409	47206	11424	11368	9948	**10913.33333**	836.4719561	YP_009195959.1	ribonucleotide reductase small subunit
AVU41_gp124	97066	97395	19006	18752	16105	12292	12241	10799	**11777.33333**	847.6451695	YP_009195960.1	hypothetical protein
AVU41_gp125	97379	97699	23791	22298	21680	15818	14964	14944	**15242**	498.9308569	YP_009195961.1	thioredoxin-like protein
AVU41_gp126	97906	98502	8806	7651	10555	3148	2761	3912	**3273.666667**	585.6998663	YP_009195962.1	hypothetical protein
AVU41_gp127	98512	98817	12562	10494	14275	8762	7388	10322	**8824**	1467.982289	YP_009195963.1	integration host factor
AVU41_gp128	98893	102111	31013	33510	42341	2056	2243	2910	**2403**	448.9198147	YP_009195964.1	DNA polymerase
AVU41_gp129	102181	102423	8199	9380	12466	7201	8316	11351	**8956**	2147.748821	YP_009195965.1	hypothetical protein
AVU41_gp130	102440	102922	15910	18319	23451	7030	8171	10743	**8648**	1901.904046	YP_009195966.1	hypothetical protein
AVU41_gp131	103009	104280	91416	91552	125481	15338	15505	21828	**17557**	3699.736883	YP_009195967.1	hypothetical protein
AVU41_gp132	104340	105596	50955	53327	61353	8652	9139	10800	**9530.333333**	1126.202616	YP_009195968.1	DNA repair protein
AVU41_gp133	105600	105953	12959	14284	15460	7813	8692	9663	**8722.666667**	925.3811827	YP_009195969.1	hypothetical protein
AVU41_gp134	105940	106602	12067	12596	15643	3884	4093	5221	**4399.333333**	719.2164718	YP_009195970.1	sigma factor
AVU41_gp135	106730	107362	31878	29859	22414	10748	10162	7835	**9581.666667**	1540.773291	YP_009195971.1	putative Ig-like protein
AVU41_gp136	107385	107897	33697	30152	24383	14019	12662	10517	**12399.33333**	1765.714114	YP_009195972.1	major tail protein
AVU41_gp137	107912	108139	19923	18041	13344	18649	17046	12950	**16215**	2938.974481	YP_009195973.1	tail protein
AVU41_gp138	108235	108495	663	694	483	542	573	409	**508**	87.12634504	YP_009195974.1	hypothetical protein
AVU41_gp139	108499	109254	3949	4476	3081	1115	1275	902	**1097.333333**	187.1265169	YP_009195975.1	hypothetical protein
AVU41_gp140	109247	110497	23377	27880	23574	3988	4801	4170	**4319.666667**	426.6641927	YP_009195976.1	DNA polymerase
AVU41_gp141	110511	110879	7774	9969	8765	4496	5820	5256	**5190.666667**	664.4135259	YP_009195977.1	hypothetical protein
AVU41_gp142	110866	111177	5423	6820	5991	3710	4709	4249	**4222.666667**	500.0203329	YP_009195978.1	hypothetical protein
AVU41_gp143	111241	111777	9692	11338	10168	3852	4548	4190	**4196.666667**	348.0478894	YP_009195979.1	hypothetical protein
AVU41_gp144	111770	112537	19858	22730	20159	5518	6376	5808	**5900.666667**	436.4416723	YP_009195980.1	hypothetical protein
AVU41_gp145	112515	112961	10472	11957	9676	5000	5762	4790	**5184**	511.456743	YP_009195981.1	hypothetical protein
AVU41_gp146	112961	113824	16185	17832	17363	3998	4446	4447	**4297**	258.9420785	YP_009195982.1	hypothetical protein
AVU41_gp147	114196	114927	18281	20746	19738	5330	6105	5966	**5800.333333**	413.2073733	YP_009195983.1	hypothetical protein
AVU41_gp148	114945	115403	14287	15585	15329	6643	7315	7390	**7116**	411.3429226	YP_009195984.1	hypothetical protein
AVU41_gp149	115468	115911	14866	16965	18288	7146	8231	9114	**8163.666667**	985.7262974	YP_009195985.1	hypothetical protein
AVU41_gp150	115928	116632	26860	26753	31296	8131	8175	9823	**8709.666667**	964.4259087	YP_009195986.1	hypothetical protein
AVU41_gp151	116694	117092	8561	8582	7207	4579	4634	3997	**4403.333333**	352.9678928	YP_009195987.1	hypothetical protein
AVU41_gp152	117239	117481	4192	3925	4341	3682	3480	3953	**3705**	237.3373127	YP_009195988.1	hypothetical protein
AVU41_gp153	117486	118043	10184	9448	10176	3895	3648	4035	**3859.333333**	195.9498235	YP_009195989.1	membrane protein
AVU41_gp154	118079	118255	5309	5072	5458	6402	6173	6823	**6466**	329.6922808	YP_009195990.1	hypothetical protein
AVU41_gp155	118248	118496	5396	4815	5812	4625	4166	5165	**4652**	500.0469978	YP_009195991.1	membrane protein
AVU41_gp156	118489	118722	4678	4008	5022	4267	3690	4749	**4235.333333**	530.2097069	YP_009195992.1	hypothetical protein
AVU41_gp157	118804	119448	857	860	862	284	287	296	**289**	6.244997998	YP_009195993.1	ribulose carboxylase/oxygenase
AVU41_gp158	119464	119712	168	216	248	144	187	220	**183.6666667**	38.10949138	YP_009195994.1	hypothetical protein
AVU41_gp159	119724	119900	383	433	434	462	527	543	**510.6666667**	42.89910644	YP_009195995.1	hypothetical protein
AVU41_gp160	119893	120189	661	723	740	475	524	551	**516.6666667**	38.52704678	YP_009195996.1	hypothetical protein

AVU41_gp161	120237	120419	402	321	462	469	378	559	**468.6666667**	90.5004604	YP_009195997.1	membrane protein
AVU41_gp162	120432	120803	1719	1298	2006	986	752	1193	**977**	220.6377121	YP_009195998.1	hypothetical protein
AVU41_gp163	120816	121163	1336	1008	1562	819	624	993	**812**	184.5995666	YP_009195999.1	hypothetical protein
AVU41_gp164	121169	121441	1052	873	1142	822	689	926	**812.3333333**	118.7953422	YP_009196000.1	membrane protein
AVU41_gp165	121511	121816	1244	1049	1363	868	738	986	**864**	124.0483777	YP_009196001.1	hypothetical protein
AVU41_gp166	121831	122181	2273	1908	2611	1382	1171	1646	**1399.666667**	237.9922968	YP_009196002.1	hypothetical protein
AVU41_gp167	122215	122394	1125	974	1233	1334	1166	1516	**1338.666667**	175.0466604	YP_009196003.1	hypothetical protein
AVU41_gp168	122620	123030	1254	937	1416	651	491	762	**634.6666667**	136.2363143	YP_009196004.1	membrane protein
AVU41_gp169	123032	123256	889	733	1090	843	702	1072	**872.3333333**	186.735999	YP_009196005.1	hypothetical protein
AVU41_gp170	123269	123469	990	745	1013	1051	798	1115	**988**	167.6275634	YP_009196006.1	hypothetical protein
AVU41_gp171	123470	123760	1035	747	1120	759	553	852	**721.3333333**	153.0174282	YP_009196007.1	membrane protein
AVU41_gp172	123853	124146	2285	1561	2555	1659	1144	1923	**1575.333333**	396.1821971	YP_009196008.1	hypothetical protein
AVU41_gp173	124143	125051	6566	4684	8461	1542	1110	2060	**1570.666667**	475.6483295	YP_009196009.1	phosphoribosyl pyrophosphate synthetase
AVU41_gp174	125069	126538	18849	12563	23224	2737	1841	3496	**2691.333333**	828.4445264	YP_009196010.1	nicotinamide phosphoribosyltransferase
AVU41_gp175	126629	126940	655	416	748	448	287	530	**421.6666667**	123.6217349	YP_009196011.1	hypothetical protein
AVU41_gp176	126957	127190	522	273	547	476	251	517	**414.6666667**	143.2142917	YP_009196012.1	hypothetical protein
AVU41_gp177	127270	127464	372	193	305	407	213	346	**322**	99.2018145	YP_009196013.1	hypothetical protein
AVU41_gp178	127478	127801	1120	650	1217	738	432	831	**667**	208.7606285	YP_009196014.1	hypothetical protein
AVU41_gp179	127814	128176	1234	743	1066	726	441	650	**605.6666667**	147.5816158	YP_009196015.1	hypothetical protein
AVU41_gp180	128176	128415	755	451	676	671	405	623	**566.3333333**	141.7650639	YP_009196016.1	hypothetical protein
AVU41_gp181	128488	128898	1495	1004	1615	776	526	869	**723.6666667**	177.387523	YP_009196017.1	hypothetical protein
AVU41_gp182	128903	129157	1092	798	1169	914	674	1014	**867.3333333**	174.737899	YP_009196018.1	hypothetical protein
AVU41_gp183	129261	129659	2499	1612	2972	1337	870	1648	**1285**	391.5980082	YP_009196019.1	hypothetical protein
AVU41_gp184	129673	130101	4533	2591	5340	2255	1301	2754	**2103.333333**	738.2779513	YP_009196020.1	hypothetical protein
AVU41_gp185	130103	130378	2635	1528	3089	2038	1193	2476	**1902.333333**	652.1704787	YP_009196021.1	hypothetical protein
AVU41_gp186	130392	130781	2403	1367	3060	1315	755	1736	**1268.666667**	492.1385306	YP_009196022.1	hypothetical protein
AVU41_gp187	130897	131523	10770	5699	12327	3666	1958	4350	**3324.666667**	1231.989177	YP_009196023.1	hypothetical protein
AVU41_gp188	131604	131720	196	124	201	358	228	380	**322**	82.14621111	YP_009196024.1	hypothetical protein
AVU41_gp189	131734	132135	884	622	1151	469	333	634	**478.6666667**	150.7326552	YP_009196025.1	hypothetical protein
AVU41_gp190	132167	132376	535	400	660	544	410	695	**549.6666667**	142.5844779	YP_009196026.1	hypothetical protein
AVU41_gp191	132376	132732	677	588	853	405	355	529	**429.6666667**	89.5842248	YP_009196027.1	hypothetical protein
AVU41_gp192	133332	133631	814	659	960	579	473	708	**586.6666667**	117.6874391	YP_009196028.1	TreA
AVU41_gp193	133647	133832	505	389	549	579	451	653	**561**	102.1958903	YP_009196029.1	TreB
AVU41_gp194	133939	134226	1328	1065	1377	984	797	1058	**946.3333333**	134.5151788	YP_009196030.1	TreC
AVU41_gp195	134226	134552	1627	1199	1726	1062	790	1168	**1006.666667**	194.9803409	YP_009196031.1	TreE
AVU41_gp196	134567	134860	803	545	828	583	399	623	**535**	119.4654762	YP_009196032.1	TreE
AVU41_gp197	134864	135049	262	156	265	301	181	315	**265.6666667**	73.65686209	YP_009196033.1	TreF
AVU41_gp198	135186	135479	570	369	598	414	270	450	**378**	95.2470472	YP_009196034.1	TreE
AVU41_gp199	135483	135740	849	567	874	702	473	750	**641.6666667**	148.0281505	YP_009196035.1	TreF
AVU41_gp200	135828	136067	1265	932	1389	1125	837	1281	**1081**	225.2465316	YP_009196036.1	hypothetical protein
AVU41_gp201	136078	136425	961	695	967	589	430	615	**544.6666667**	100.1515518	YP_009196037.1	hypothetical protein
AVU41_gp202	136630	136968	124	90	129	78	57	84	**73**	14.17744688	YP_009196038.1	hypothetical protein
AVU41_gp203	137279	137587	723	608	635	499	424	455	**459.3333333**	37.68730998	YP_009196039.1	TreJ
AVU41_gp204	137793	138077	1200	1044	1143	899	789	887	**858.3333333**	60.3434614	YP_009196040.1	hypothetical protein
AVU41_gp205	138152	138343	55	43	33	61	48	38	**49**	11.53256259	YP_009196041.1	hypothetical protein
AVU41_gp206	138843	139325	1159	1344	1542	512	599	706	**605.6666667**	97.1716694	YP_009196042.1	HNH homing endonuclease
AVU41_gp207	139493	139651	27	23	23	36	31	32	**33**	2.645751311	YP_009196043.1	TreN
AVU41_gp208	139724	139870	94	84	83	136	123	125	**128**	7	YP_009196044.1	hypothetical protein
AVU41_gp209	140036	140359	1470	1070	1477	968	711	1009	**896**	161.5208965	YP_009196045.1	TreP
AVU41_gp210	140445	140840	2216	1589	2761	1194	864	1543	**1200.333333**	339.5443025	YP_009196046.1	hypothetical protein
AVU41_gp211	141309	141530	1455	708	1361	1399	687	1357	**1147.666667**	399.5013559	YP_009196047.1	hypothetical protein
AVU41_gp212	141789	141953	1891	1127	1640	2446	1471	2199	**2038.666667**	506.8888767	YP_009196048.1	hypothetical protein
AVU41_gp213	142033	142269	1734	1037	1848	1562	943	1725	**1410**	412.5639344	YP_009196049.1	hypothetical protein

Table S2. Full list of genes dysregulated in biofilms treated with subinhibitory doses of phiIPLA-RODI compared to untreated biofilms according to RNA-seq

Gene ID	Gene name	Gene product	Fold-change	padj
SAOUHSC_00003	SAOUHSC_00003	Putative uncharacterized protein	-2.66	1.04E-06
SAOUHSC_00004	recF	DNA replication and repair protein RecF	-2.47	1.08E-07
SAOUHSC_00005	gyrB	DNA gyrase subunit B	-2.89	1.77E-13
SAOUHSC_00006	gyrA	DNA gyrase subunit A	-3.92	6.47E-19
SAOUHSC_00008	hutH	Histidine ammonia-lyase	-2.23	9.86E-10
SAOUHSC_00010	SAOUHSC_00010	Putative uncharacterized protein	2.03	7.94E-04
SAOUHSC_00012	SAOUHSC_00012	Putative uncharacterized protein	2.10	2.57E-03
SAOUHSC_00021	walK	Sensor protein kinase WalK	-3.80	1.15E-28
SAOUHSC_00022	SAOUHSC_00022	Putative uncharacterized protein	-5.44	3.28E-37
SAOUHSC_00023	SAOUHSC_00023	Putative uncharacterized protein	-5.48	3.85E-40
SAOUHSC_00027	rlmH	Ribosomal RNA large subunit methyltransferase H	-2.15	3.76E-08
SAOUHSC_00036	SAOUHSC_00036	Putative uncharacterized protein	-2.19	3.48E-05
SAOUHSC_00057	SAOUHSC_00057	Putative uncharacterized protein	-4.02	3.53E-22
SAOUHSC_00058	SAOUHSC_00058	Putative uncharacterized protein	-4.03	7.96E-21
SAOUHSC_00060	SAOUHSC_00060	Putative uncharacterized protein	3.26	8.01E-10
SAOUHSC_00061	SAOUHSC_00061	Putative uncharacterized protein	-2.89	2.06E-16
SAOUHSC_00074	SAOUHSC_00074	Periplasmic binding protein, putative	4.80	5.34E-04
SAOUHSC_00081	SAOUHSC_00081	Putative uncharacterized protein	-3.00	2.33E-11
SAOUHSC_00082	SAOUHSC_00082	Putative uncharacterized protein	-2.69	2.69E-14
SAOUHSC_00083	SAOUHSC_00083	Putative uncharacterized protein	-2.29	5.67E-11
SAOUHSC_00085	SAOUHSC_00085	Putative uncharacterized protein	2.93	8.68E-10
SAOUHSC_00086	SAOUHSC_00086	3-ketoacyl-acyl carrier protein reductase, putative	-10.40	6.84E-35
SAOUHSC_00094	SAOUHSC_00094	Uncharacterized protein SAOUHSC_00094	-16.68	1.82E-33
SAOUHSC_00097			-2.62	1.40E-04
SAOUHSC_00100			-3.72	1.79E-19
SAOUHSC_00101	deoB	Phosphopentomutase	-8.81	3.25E-41
SAOUHSC_00102	SAOUHSC_00102	Phosphonates ABC transporter, permease protein CC0363, puta	-2.08	1.39E-05
SAOUHSC_00103	SAOUHSC_00103	Phosphonates ABC transporter, permease protein CC0363, puta	-2.00	6.68E-04
SAOUHSC_00104	phnC	Phosphonates import ATP-binding protein PhnC	-2.41	1.89E-05
SAOUHSC_00106	SAOUHSC_00106	Putative uncharacterized protein	4.17	8.38E-09
SAOUHSC_00114	SAOUHSC_00114	Capsular polysaccharide biosynthesis protein, putative	2.29	1.30E-03
SAOUHSC_00121	SAOUHSC_00121	Capsular polysaccharide synthesis enzyme O-acetyl transferase	2.03	2.43E-03
SAOUHSC_00129	SAOUHSC_00129	UDP-N-acetylglucosamine 2-epimerase	-2.34	1.34E-13
SAOUHSC_00134	SAOUHSC_00134	Putative uncharacterized protein	2.34	6.24E-03
SAOUHSC_00142	SAOUHSC_00142	Formate dehydrogenase, NAD-dependent, putative	-7.47	2.44E-21
SAOUHSC_00158	SAOUHSC_00158	PTS system EIIBC component SAOUHSC_00158	-2.34	2.87E-04
SAOUHSC_00160	SAOUHSC_00160	Putative uncharacterized protein	-2.11	2.50E-03
SAOUHSC_00162	SAOUHSC_00162	Type I site-specific deoxyribonuclease, HsdR family, putative	2.39	6.56E-13
SAOUHSC_00163	SAOUHSC_00163	Putative uncharacterized protein	-2.14	1.31E-05
SAOUHSC_00171	SAOUHSC_00171	Gamma-glutamyltranspeptidase, putative	-2.65	1.12E-11
SAOUHSC_00173	azoR	FMN-dependent NADH-azoreductase	-3.42	5.36E-16
SAOUHSC_00178	SAOUHSC_00178	Maltose ABC transporter, permease protein	-2.67	1.10E-05
SAOUHSC_00179	SAOUHSC_00179	Putative uncharacterized protein	-2.76	2.12E-07
SAOUHSC_00180	SAOUHSC_00180	Putative uncharacterized protein	-2.45	3.67E-09
SAOUHSC_00181	SAOUHSC_00181	Putative uncharacterized protein	-3.03	2.25E-10
SAOUHSC_00185	SAOUHSC_00185	Uncharacterized sensor-like histidine kinase SAOUHSC_00185	3.20	1.13E-07
SAOUHSC_00186	SAOUHSC_00186	Lipoprotein, putative	2.46	1.51E-06
SAOUHSC_00187	pflB	Formate acetyltransferase	-4.57	3.18E-26
SAOUHSC_00188	pflA	Pyruvate formate-lyase-activating enzyme	-5.52	2.45E-50
SAOUHSC_00189	SAOUHSC_00189	Putative uncharacterized protein	-3.08	9.56E-13
SAOUHSC_00195	SAOUHSC_00195	Acetyl-CoA acetyltransferase, putative	-2.72	7.58E-06
SAOUHSC_00201	SAOUHSC_00201	Putative uncharacterized protein	-2.14	1.78E-13
SAOUHSC_00202	SAOUHSC_00202	Putative uncharacterized protein	3.53	2.78E-22
SAOUHSC_00203	SAOUHSC_00203	Putative uncharacterized protein	2.20	1.15E-07
SAOUHSC_00204	SAOUHSC_00204	Globin domain protein	2.52	6.01E-28
SAOUHSC_00216	SAOUHSC_00216	PTS system component	-2.24	4.42E-07
SAOUHSC_00217	SAOUHSC_00217	Sorbitol dehydrogenase, putative	-2.62	2.75E-07
SAOUHSC_00218	SAOUHSC_00218	Putative uncharacterized protein	-3.02	1.23E-05
SAOUHSC_00219	SAOUHSC_00219	Putative uncharacterized protein	-3.44	5.04E-15
SAOUHSC_00220	SAOUHSC_00220	Putative uncharacterized protein	3.10	7.97E-10
SAOUHSC_00221	SAOUHSC_00221	Alcohol dehydrogenase, zinc-containing	2.09	8.18E-07
SAOUHSC_00222	SAOUHSC_00222	TagB protein, putative	3.48	1.14E-13
SAOUHSC_00231	lytR	Sensory transduction protein LytR	3.14	2.16E-14
SAOUHSC_00234	SAOUHSC_00234	Putative uncharacterized protein	-2.25	7.25E-17
SAOUHSC_00242	SAOUHSC_00242	Putative uncharacterized protein	-2.58	3.56E-11
SAOUHSC_00247	SAOUHSC_00247	Putative uncharacterized protein	-3.77	1.24E-22
SAOUHSC_00249	SAOUHSC_00249	Putative uncharacterized protein	2.15	3.65E-04
SAOUHSC_00250	SAOUHSC_00250	Putative uncharacterized protein	2.41	2.61E-05
SAOUHSC_00251	SAOUHSC_00251	Putative uncharacterized protein	3.86	1.74E-16
SAOUHSC_00253	SAOUHSC_00253	Putative uncharacterized protein	3.43	3.91E-26
SAOUHSC_00260	SAOUHSC_00260	Putative uncharacterized protein	3.35	4.32E-05
SAOUHSC_00261	SAOUHSC_00261	Putative uncharacterized protein	2.40	5.01E-06
SAOUHSC_00282	SAOUHSC_00282	Branched-chain amino acid transport system II carrier protein	6.01	4.13E-14
SAOUHSC_00284	SAOUHSC_00284	5'-nucleotidase, lipoprotein e(P4) family	4.72	2.26E-14
SAOUHSC_00289	SAOUHSC_00289	Putative uncharacterized protein	4.48	2.56E-04
SAOUHSC_00293	SAOUHSC_00293	Putative uncharacterized protein	-3.47	4.31E-05
SAOUHSC_00297	SAOUHSC_00297	Putative uncharacterized protein	3.34	4.75E-07
SAOUHSC_00302	SAOUHSC_00302	Putative uncharacterized protein	3.39	2.96E-07
SAOUHSC_00316	SAOUHSC_00316	Putative uncharacterized protein	4.09	7.04E-07
SAOUHSC_00319	SAOUHSC_00319	Putative uncharacterized protein	-2.87	7.08E-20
SAOUHSC_00320	SAOUHSC_00320	NADPH-dependent FMN reductase, putative	-4.02	1.51E-35
SAOUHSC_00325	SAOUHSC_00325	Efem/EfeO family lipoprotein	-2.43	1.18E-03
SAOUHSC_00326	SAOUHSC_00326	Putative uncharacterized protein	-2.54	1.51E-06
SAOUHSC_00327	SAOUHSC_00327	Putative uncharacterized protein	-2.35	1.08E-06

SAOUHSC_00328	tatC	Sec-independent protein translocase protein TatC	2.67	5.87E-08
SAOUHSC_00330	SAOUHSC_00330	Putative uncharacterized protein	8.48	1.17E-11
SAOUHSC_00331	SAOUHSC_00331	Putative uncharacterized protein	-3.01	7.86E-05
SAOUHSC_00332	SAOUHSC_00332	Putative uncharacterized protein	-6.06	1.29E-30
SAOUHSC_00333	SAOUHSC_00333	ABC transporter, ATP-binding protein, putative	-6.62	4.59E-47
SAOUHSC_00334	SAOUHSC_00334	Putative uncharacterized protein	-6.55	1.08E-44
SAOUHSC_00335	SAOUHSC_00335	Putative uncharacterized protein	7.83	8.67E-12
SAOUHSC_00336	SAOUHSC_00336	Probable acetyl-CoA acyltransferase	-2.77	5.35E-22
SAOUHSC_00344	SAOUHSC_00344	Putative uncharacterized protein	-2.07	9.41E-07
SAOUHSC_00346	ychF	Ribosome-binding ATPase YchF	-2.44	3.07E-11
SAOUHSC_00347	SAOUHSC_00347	Putative uncharacterized protein	-2.84	1.21E-10
SAOUHSC_00349	SAOUHSC_00349	Single-stranded DNA-binding protein	-2.67	2.49E-18
SAOUHSC_00350	rpsR	30S ribosomal protein S18	-2.69	2.34E-18
SAOUHSC_00357	SAOUHSC_00357	Putative uncharacterized protein	2.15	2.20E-04
SAOUHSC_00359	SAOUHSC_00359	Phosphoglycerate mutase family protein	2.18	5.63E-08
SAOUHSC_00360	SAOUHSC_00360	Putative uncharacterized protein	2.01	6.68E-06
SAOUHSC_00363	SAOUHSC_00363	Putative uncharacterized protein	2.62	6.15E-06
SAOUHSC_00364	ahpF	Alkyl hydroperoxide reductase subunit F	-3.86	4.38E-21
SAOUHSC_00371	SAOUHSC_00371	Putative uncharacterized protein	-3.04	1.70E-15
SAOUHSC_00372	xpt	Xanthine phosphoribosyltransferase	8.25	3.11E-34
SAOUHSC_00373	SAOUHSC_00373	Xanthine permease, putative	5.23	7.80E-31
SAOUHSC_00381	SAOUHSC_00381	Putative uncharacterized protein	-2.44	3.75E-10
SAOUHSC_00397	SAOUHSC_00397	Type I restriction-modification system, M subunit	2.58	2.15E-12
SAOUHSC_00398	SAOUHSC_00398	Restriction modification system specificity subunit, putative	4.07	2.14E-22
SAOUHSC_00406	SAOUHSC_00406	Putative uncharacterized protein	2.78	3.62E-05
SAOUHSC_00407	SAOUHSC_00407	Putative uncharacterized protein	4.20	1.31E-07
SAOUHSC_00409	SAOUHSC_00409	Putative uncharacterized protein	-4.81	8.51E-20
SAOUHSC_00410	SAOUHSC_00410	Putative uncharacterized protein	-2.04	1.67E-05
SAOUHSC_00411	SAOUHSC_00411	Putative uncharacterized protein	-23.42	6.39E-22
SAOUHSC_00413	SAOUHSC_00413	UPF0753 protein SAOUHSC_00413	-3.05	9.59E-09
SAOUHSC_00414	SAOUHSC_00414	Putative uncharacterized protein	-2.74	7.44E-08
SAOUHSC_00417	SAOUHSC_00417	Putative uncharacterized protein	-2.21	3.13E-14
SAOUHSC_00424	SAOUHSC_00424	ABC transporter, permease protein, putative	-3.15	2.47E-04
SAOUHSC_00426	SAOUHSC_00426	Lipoprotein	-2.71	3.36E-06
SAOUHSC_00427	sle1	N-acetylmuramoyl-L-alanine amidase sle1	10.27	1.78E-100
SAOUHSC_00429	SAOUHSC_00429	MutT/nudix family protein, putative	-2.01	2.00E-04
SAOUHSC_00430	SAOUHSC_00430	Putative uncharacterized protein	-3.25	4.39E-14
SAOUHSC_00431	SAOUHSC_00431	Putative uncharacterized protein	2.70	4.57E-07
SAOUHSC_00433	SAOUHSC_00433	Putative uncharacterized protein	4.34	3.53E-20
SAOUHSC_00434	SAOUHSC_00434	Transcriptional regulator, lysR family, putative	2.43	1.34E-05
SAOUHSC_00437	SAOUHSC_00437	Putative uncharacterized protein	-6.65	1.26E-20
SAOUHSC_00438	SAOUHSC_00438	Alpha amylase family protein, putative	-5.06	2.93E-14
SAOUHSC_00439	SAOUHSC_00439	Putative uncharacterized protein	-4.40	8.15E-16
SAOUHSC_00440	SAOUHSC_00440	Putative uncharacterized protein	-9.37	3.96E-36
SAOUHSC_00450	SAOUHSC_00450	Orn/Lys/Arg decarboxylase, putative	2.55	3.97E-11
SAOUHSC_00451	tmk	Thymidylate kinase	2.56	3.72E-13
SAOUHSC_00455	SAOUHSC_00455	Putative uncharacterized protein	-2.22	1.38E-14
SAOUHSC_00456	SAOUHSC_00456	Putative uncharacterized protein	-3.48	3.54E-23
SAOUHSC_00457	SAOUHSC_00457	Putative uncharacterized protein	-3.15	4.12E-24
SAOUHSC_00458	SAOUHSC_00458	UPF0213 protein SAOUHSC_00458	-2.59	8.31E-13
SAOUHSC_00459	rsmI	Ribosomal RNA small subunit methyltransferase I	-2.75	2.27E-10
SAOUHSC_00461	metG	Methionine--tRNA ligase	-3.00	2.86E-19
SAOUHSC_00462	SAOUHSC_00462	Putative uncharacterized protein	-3.96	1.34E-28
SAOUHSC_00468	SAOUHSC_00468	Putative uncharacterized protein	-2.97	3.81E-11
SAOUHSC_00469	spoVG	Putative septation protein SpoVG	-2.71	4.22E-12
SAOUHSC_00471	glmU	Bifunctional protein GlmU	-2.73	1.28E-10
SAOUHSC_00472	prs	Ribose-phosphate pyrophosphokinase	-2.22	1.20E-12
SAOUHSC_00473	SAOUHSC_00473	Putative uncharacterized protein	-2.18	7.81E-13
SAOUHSC_00474	rplY	50S ribosomal protein L25	-2.06	2.24E-12
SAOUHSC_00479	SAOUHSC_00479	Putative uncharacterized protein	-2.03	4.17E-11
SAOUHSC_00482	SAOUHSC_00482	Putative uncharacterized protein	-2.52	9.79E-11
SAOUHSC_00484	tilS	tRNA(Ile)-lysidine synthase	2.13	9.47E-10
SAOUHSC_00485	SAOUHSC_00485	Hypoxanthine phosphoribosyltransferase	2.00	5.39E-05
SAOUHSC_00489	SAOUHSC_00489	Dihydropteroate synthase	-2.52	1.03E-15
SAOUHSC_00490	SAOUHSC_00490	Dihydroneopterin aldolase	-2.82	5.54E-13
SAOUHSC_00491	SAOUHSC_00491	2-amino-4-hydroxy-6-hydroxymethyldihydropteridine pyrophos	-3.28	1.61E-23
SAOUHSC_00493	lysS	Lysine--tRNA ligase	-3.26	1.22E-20
SAOUHSC_00500	pdxT	Glutamine amidotransferase subunit PdxT	-2.03	2.66E-06
SAOUHSC_00502	ctsR	Transcriptional regulator CtsR	-7.24	7.02E-22
SAOUHSC_00503	SAOUHSC_00503	UvrB/uvrC motif domain protein	-9.09	4.61E-27
SAOUHSC_00504	SAOUHSC_00504	Putative ATP:guanido phosphotransferase SAOUHSC_00504	-10.59	7.91E-32
SAOUHSC_00505	clpC	ATP-dependent Clp protease ATP-binding subunit ClpC	-22.97	1.00E-52
SAOUHSC_00506	SAOUHSC_00506	Putative uncharacterized protein	-23.28	1.17E-58
SAOUHSC_00515	SAOUHSC_00515	Putative uncharacterized protein	2.51	4.36E-16
SAOUHSC_00516	secE	Protein translocase subunit SecE	2.42	2.06E-16
SAOUHSC_00517	nusG	Transcription termination/antitermination protein NusG	2.13	4.22E-15
SAOUHSC_00524	rpoB	DNA-directed RNA polymerase subunit beta	-2.92	2.05E-18
SAOUHSC_00525	rpoC	DNA-directed RNA polymerase subunit beta'	-5.03	2.50E-35
SAOUHSC_00528	rpsG	30S ribosomal protein S7	-2.07	1.17E-09
SAOUHSC_00529	fusA	Elongation factor G	-2.56	1.46E-16
SAOUHSC_00531	SAOUHSC_00531	Putative uncharacterized protein	2.27	6.05E-11
SAOUHSC_00533	hchA	Molecular chaperone Hsp31 and glyoxalase 3	3.07	1.39E-11
SAOUHSC_00538	SAOUHSC_00538	Haloacid dehalogenase-like hydrolase, putative	2.95	2.00E-18
SAOUHSC_00539	SAOUHSC_00539	Putative uncharacterized protein	3.58	3.34E-13
SAOUHSC_00540	SAOUHSC_00540	Putative uncharacterized protein	4.36	6.71E-06
SAOUHSC_00544	sdrC	Serine-aspartate repeat-containing protein C	-3.90	6.13E-21

SAOUHSC_00545	sdrD	Serine-aspartate repeat-containing protein D	-5.59	4.67E-22
SAOUHSC_00550	SAOUHSC_00550	Putative uncharacterized protein	2.86	1.77E-22
SAOUHSC_00551	SAOUHSC_00551	Putative uncharacterized protein	4.85	2.97E-31
SAOUHSC_00552	nagB	Glucosamine-6-phosphate deaminase	-2.15	1.58E-06
SAOUHSC_00556	SAOUHSC_00556	Proline/betaine transporter, putative	3.42	1.39E-15
SAOUHSC_00557	SAOUHSC_00557	Putative uncharacterized protein	3.61	9.95E-06
SAOUHSC_00559	SAOUHSC_00559	Putative uncharacterized protein	-2.91	7.70E-07
SAOUHSC_00560	SAOUHSC_00560	Putative uncharacterized protein	-4.22	4.90E-13
SAOUHSC_00561	SAOUHSC_00561	Putative uncharacterized protein	-8.57	1.14E-40
SAOUHSC_00562	SAOUHSC_00562	Phosphomethylpyrimidine kinase	2.37	4.59E-17
SAOUHSC_00564	ung	Uracil-DNA glycosylase	3.34	8.45E-15
SAOUHSC_00565	SAOUHSC_00565	Putative uncharacterized protein	3.18	5.18E-11
SAOUHSC_00569	SAOUHSC_00569	Putative uncharacterized protein	3.76	6.54E-07
SAOUHSC_00571	SAOUHSC_00571	Putative uncharacterized protein	2.12	3.96E-04
SAOUHSC_00572	SAOUHSC_00572	Putative uncharacterized protein	2.36	1.28E-05
SAOUHSC_00573	SAOUHSC_00573	Putative heme-dependent peroxidase SAOUHSC_00573	2.90	5.53E-19
SAOUHSC_00577	SAOUHSC_00577	Mevalonate kinase, putative	-2.47	1.18E-12
SAOUHSC_00578	SAOUHSC_00578	Diphosphomevalonate decarboxylase	-3.65	3.17E-20
SAOUHSC_00579	SAOUHSC_00579	Phosphomevalonate kinase	-4.77	7.94E-28
SAOUHSC_00580	SAOUHSC_00580	UPF0741 protein SAOUHSC_00580	-4.22	1.09E-33
SAOUHSC_00585	SAOUHSC_00585	Putative uncharacterized protein	2.69	3.17E-03
SAOUHSC_00586	SAOUHSC_00586	Putative uncharacterized protein	2.60	1.21E-05
SAOUHSC_00587	SAOUHSC_00587	Putative uncharacterized protein	2.02	6.94E-05
SAOUHSC_00588	SAOUHSC_00588	Putative uncharacterized protein	2.98	4.74E-05
SAOUHSC_00589	SAOUHSC_00589	Putative uncharacterized protein	3.86	9.61E-04
SAOUHSC_00591	SAOUHSC_00591	Putative uncharacterized protein	3.67	1.76E-05
SAOUHSC_00592	SAOUHSC_00592	Putative uncharacterized protein	3.43	1.95E-03
SAOUHSC_00596	SAOUHSC_00596	Putative uncharacterized protein	3.52	2.13E-03
SAOUHSC_00603	SAOUHSC_00603	Putative uncharacterized protein	-3.07	2.91E-28
SAOUHSC_00604	SAOUHSC_00604	Putative uncharacterized protein	-2.67	2.07E-23
SAOUHSC_00605	SAOUHSC_00605	Putative uncharacterized protein	3.13	5.29E-11
SAOUHSC_00608	adh	Alcohol dehydrogenase	-6.51	3.30E-40
SAOUHSC_00609	SAOUHSC_00609	Putative uncharacterized protein	-5.30	1.67E-22
SAOUHSC_00610	SAOUHSC_00610	Putative uncharacterized protein	2.65	2.89E-12
SAOUHSC_00611	argS	Arginine--tRNA ligase	2.00	5.76E-12
SAOUHSC_00613	SAOUHSC_00613	Iron compound ABC transporter, substrate-binding protein, put	3.14	4.44E-11
SAOUHSC_00617	SAOUHSC_00617	Putative uncharacterized protein	-5.04	4.56E-15
SAOUHSC_00619	SAOUHSC_00619	Putative uncharacterized protein	-8.10	1.26E-51
SAOUHSC_00624	SAOUHSC_00624	Integrase/recombinase, putative	5.92	1.84E-03
SAOUHSC_00625	mnhA2	Putative antiporter subunit mnhA2	3.98	4.62E-03
SAOUHSC_00626	mnhB2	Putative antiporter subunit mnhB2	4.40	1.18E-05
SAOUHSC_00627	mnhC2	Putative antiporter subunit mnhC2	2.99	8.80E-03
SAOUHSC_00640	SAOUHSC_00640	Putative uncharacterized protein	4.36	2.47E-22
SAOUHSC_00641	tagH	Teichoic acids export ATP-binding protein TagH	-2.09	8.94E-12
SAOUHSC_00646	SAOUHSC_00646	Penicillin-binding protein 4, putative	2.65	3.08E-06
SAOUHSC_00647	SAOUHSC_00647	Putative uncharacterized protein	-2.20	1.02E-09
SAOUHSC_00648	SAOUHSC_00648	Putative uncharacterized protein	-3.53	1.68E-27
SAOUHSC_00651	SAOUHSC_00651	Putative uncharacterized protein	-2.83	5.52E-09
SAOUHSC_00655	SAOUHSC_00655	Putative uncharacterized protein	-2.65	1.63E-12
SAOUHSC_00656	SAOUHSC_00656	Putative uncharacterized protein	-3.67	1.41E-18
SAOUHSC_00658	SAOUHSC_00658	Putative uncharacterized protein	-4.75	1.62E-29
SAOUHSC_00661	SAOUHSC_00661	Putative uncharacterized protein	4.71	1.88E-11
SAOUHSC_00663	SAOUHSC_00663	Putative uncharacterized protein	3.20	1.77E-25
SAOUHSC_00667	SAOUHSC_00667	ABC transporter ATP-binding protein, putative	2.57	1.04E-14
SAOUHSC_00668	SAOUHSC_00668	ABC transporter permease, putative	2.77	7.13E-11
SAOUHSC_00671	SAOUHSC_00671	Secretory antigen SsaA-like protein	9.36	5.45E-82
SAOUHSC_00672	SAOUHSC_00672	Putative uncharacterized protein	2.57	5.58E-07
SAOUHSC_00673	SAOUHSC_00673	Putative uncharacterized protein	3.79	5.96E-08
SAOUHSC_00683	SAOUHSC_00683	Putative uncharacterized protein	4.44	1.76E-11
SAOUHSC_00685	SAOUHSC_00685	Putative uncharacterized protein	4.50	3.70E-17
SAOUHSC_00686	SAOUHSC_00686	Putative uncharacterized protein	3.46	7.18E-10
SAOUHSC_00687	SAOUHSC_00687	Putative uncharacterized protein	3.60	7.11E-09
SAOUHSC_00690	SAOUHSC_00690	Putative uncharacterized protein	3.09	9.99E-09
SAOUHSC_00691	uppP	Undecaprenyl-diphosphatase	2.04	1.96E-07
SAOUHSC_00693	SAOUHSC_00693	Putative uncharacterized protein	-2.76	1.65E-11
SAOUHSC_00696	SAOUHSC_00696	Putative uncharacterized protein	2.75	8.49E-18
SAOUHSC_00697	SAOUHSC_00697	Putative uncharacterized protein	2.87	4.29E-10
SAOUHSC_00698	SAOUHSC_00698	Putative uncharacterized protein	-3.65	1.06E-32
SAOUHSC_00699	SAOUHSC_00699	Deoxyribodipyrimidine photolyase, putative	-3.27	9.51E-26
SAOUHSC_00701	SAOUHSC_00701	Putative uncharacterized protein	2.45	5.44E-06
SAOUHSC_00704	SAOUHSC_00704	Putative uncharacterized protein	12.44	2.70E-39
SAOUHSC_00706	SAOUHSC_00706	Putative uncharacterized protein	-4.52	9.66E-19
SAOUHSC_00707	SAOUHSC_00707	Tagatose-6-phosphate kinase	-5.98	1.50E-28
SAOUHSC_00708	SAOUHSC_00708	Fructose specific permease, putative	-6.17	1.15E-33
SAOUHSC_00709	SAOUHSC_00709	Putative uncharacterized protein	-6.11	2.25E-20
SAOUHSC_00710	SAOUHSC_00710	N-acetylglucosamine-6-phosphate deacetylase	3.32	3.82E-26
SAOUHSC_00711	SAOUHSC_00711	Putative uncharacterized protein	3.43	5.08E-30
SAOUHSC_00729	SAOUHSC_00729	ABC transporter, ATP-binding protein	3.17	1.41E-26
SAOUHSC_00731	SAOUHSC_00731	ABC transporter domain protein	3.08	8.41E-20
SAOUHSC_00732	SAOUHSC_00732	Amino acid ABC transporter, permease protein, putative	2.12	2.88E-13
SAOUHSC_00733	hisC	Histidinol-phosphate aminotransferase	3.53	8.89E-25
SAOUHSC_00738	SAOUHSC_00738	Putative uncharacterized protein	10.46	4.57E-42
SAOUHSC_00743	SAOUHSC_00743	Ribonucleotide-disphosphate reductase beta chain, putative	3.32	3.38E-23
SAOUHSC_00744	SAOUHSC_00744	Putative uncharacterized protein	3.78	1.74E-05
SAOUHSC_00746	SAOUHSC_00746	Putative uncharacterized protein	2.70	3.37E-04
SAOUHSC_00753	SAOUHSC_00753	Putative uncharacterized protein	3.03	7.42E-08

SAOUHSC_00756	SAOUHSC_00756	Putative uncharacterized protein	-3.27	4.03E-19
SAOUHSC_00762	SAOUHSC_00762	Putative uncharacterized protein	2.03	7.67E-07
SAOUHSC_00763	SAOUHSC_00763	Putative uncharacterized protein	2.36	9.62E-11
SAOUHSC_00767	SAOUHSC_00767	Uncharacterized protein SAOUHSC_00767	-4.04	2.17E-19
SAOUHSC_00770	SAOUHSC_00770	Putative uncharacterized protein	-2.09	7.94E-06
SAOUHSC_00774	SAOUHSC_00774	Putative uncharacterized protein	2.00	2.68E-03
SAOUHSC_00775	SAOUHSC_00775	Putative uncharacterized protein	2.94	1.21E-04
SAOUHSC_00780	uvrA	UvrABC system protein A	-2.07	3.89E-06
SAOUHSC_00781	hprK	HPr kinase/phosphorylase	2.37	9.76E-17
SAOUHSC_00782	lgt	Prolipoprotein diacylglyceryl transferase	2.53	3.43E-18
SAOUHSC_00784	SAOUHSC_00784	Putative uncharacterized protein	-2.30	9.20E-14
SAOUHSC_00786	SAOUHSC_00786	Putative uncharacterized protein	-2.47	7.61E-08
SAOUHSC_00789	whiA	Putative sporulation transcription regulator WhiA	-2.75	7.89E-21
SAOUHSC_00793	SAOUHSC_00793	Putative uncharacterized protein	2.50	1.93E-10
SAOUHSC_00795	SAOUHSC_00795	Glyceraldehyde-3-phosphate dehydrogenase, type I	-6.94	3.47E-38
SAOUHSC_00796	pgk	Phosphoglycerate kinase	-2.90	1.58E-19
SAOUHSC_00797	tpiA	Triosephosphate isomerase	-3.32	1.27E-19
SAOUHSC_00798	gpmI	2,3-bisphosphoglycerate-independent phosphoglycerate mutas	-3.49	1.16E-22
SAOUHSC_00799	eno	Enolase	-4.31	6.68E-30
SAOUHSC_00802	SAOUHSC_00802	Carboxylesterase, putative	-2.11	5.28E-09
SAOUHSC_00803	rnr	Ribonuclease R	-2.98	7.80E-15
SAOUHSC_00804	smpB	SsrA-binding protein	-3.12	3.94E-13
SAOUHSC_00818	SAOUHSC_00818	Thermonuclease	5.72	7.07E-08
SAOUHSC_00820	SAOUHSC_00820	Putative uncharacterized protein	3.91	1.17E-05
SAOUHSC_00821	SAOUHSC_00821	Putative uncharacterized protein	4.84	1.49E-06
SAOUHSC_00822	SAOUHSC_00822	Putative uncharacterized protein	3.67	7.88E-06
SAOUHSC_00823	SAOUHSC_00823	Putative uncharacterized protein	-5.53	1.14E-12
SAOUHSC_00824	SAOUHSC_00824	Putative uncharacterized protein	-2.54	5.48E-07
SAOUHSC_00828	SAOUHSC_00828	Putative uncharacterized protein	2.60	7.60E-04
SAOUHSC_00830	SAOUHSC_00830	Putative uncharacterized protein	-2.89	1.54E-18
SAOUHSC_00834	SAOUHSC_00834	Thioredoxin, putative	2.54	4.39E-09
SAOUHSC_00835	SAOUHSC_00835	Putative uncharacterized protein	2.01	2.18E-07
SAOUHSC_00843	SAOUHSC_00843	Putative uncharacterized protein	2.04	4.47E-10
SAOUHSC_00844	SAOUHSC_00844	Lipoprotein	2.15	2.02E-14
SAOUHSC_00848	SAOUHSC_00848	Putative uncharacterized protein	-2.20	2.40E-16
SAOUHSC_00849	SAOUHSC_00849	Aminotransferase, class V superfamily, putative	-2.63	8.02E-15
SAOUHSC_00850	SAOUHSC_00850	Putative uncharacterized protein	-2.54	1.30E-15
SAOUHSC_00851	SAOUHSC_00851	UPF0051 protein SAOUHSC_00851	-3.01	4.12E-25
SAOUHSC_00858	SAOUHSC_00858	Putative uncharacterized protein	2.27	2.57E-11
SAOUHSC_00862	SAOUHSC_00862	Putative uncharacterized protein	3.06	2.67E-13
SAOUHSC_00863	SAOUHSC_00863	Putative uncharacterized protein	3.36	5.42E-10
SAOUHSC_00864	SAOUHSC_00864	Putative uncharacterized protein	5.91	1.61E-38
SAOUHSC_00865	nagD	Protein NagD homolog	3.24	1.89E-29
SAOUHSC_00867	SAOUHSC_00867	Putative uncharacterized protein	5.04	3.53E-07
SAOUHSC_00868	SAOUHSC_00868	Putative uncharacterized protein	7.17	3.00E-39
SAOUHSC_00869	dltA	D-alanine--poly(phosphoribitol) ligase subunit 1	3.69	1.12E-28
SAOUHSC_00870	SAOUHSC_00870	DltB protein, putative	2.33	1.50E-08
SAOUHSC_00871	dltC	D-alanine--poly(phosphoribitol) ligase subunit 2	2.44	7.64E-07
SAOUHSC_00876	SAOUHSC_00876	UPF0349 protein SAOUHSC_00876	2.93	1.09E-15
SAOUHSC_00878	SAOUHSC_00878	NADH dehydrogenase-like protein SAOUHSC_00878	-6.42	1.68E-60
SAOUHSC_00882	SAOUHSC_00882	Putative uncharacterized protein	-2.20	1.20E-05
SAOUHSC_00888	mnhB1	Na(+)/H(+) antiporter subunit B1	2.68	6.94E-08
SAOUHSC_00889	mnhA1	Na(+)/H(+) antiporter subunit A1	2.92	8.79E-26
SAOUHSC_00890	SAOUHSC_00890	Putative uncharacterized protein	2.61	2.44E-08
SAOUHSC_00893	SAOUHSC_00893	FMN oxidoreductase, putative	4.48	2.68E-12
SAOUHSC_00895	SAOUHSC_00895	Glutamate dehydrogenase	-2.84	3.08E-22
SAOUHSC_00896	SAOUHSC_00896	Putative uncharacterized protein	-3.28	2.89E-09
SAOUHSC_00897	SAOUHSC_00897	Putative uncharacterized protein	2.05	2.17E-07
SAOUHSC_00901	SAOUHSC_00901	Putative uncharacterized protein	3.37	4.96E-11
SAOUHSC_00902	SAOUHSC_00902	Signal peptidase IA, putative	2.97	2.07E-10
SAOUHSC_00909	SAOUHSC_00909	Putative uncharacterized protein	2.60	9.76E-16
SAOUHSC_00912	SAOUHSC_00912	ATP-dependent Clp protease, ATP-binding subunit ClpB	-112.83	1.68E-85
SAOUHSC_00913	SAOUHSC_00913	Putative uncharacterized protein	-140.47	1.98E-118
SAOUHSC_00914	SAOUHSC_00914	2-isopropylmalate synthase, putative	-3.13	4.78E-04
SAOUHSC_00916	SAOUHSC_00916	Putative uncharacterized protein	2.83	7.31E-03
SAOUHSC_00923	SAOUHSC_00923	Putative uncharacterized protein	7.86	4.00E-12
SAOUHSC_00924	SAOUHSC_00924	Putative uncharacterized protein	7.06	3.95E-10
SAOUHSC_00925	SAOUHSC_00925	Putative uncharacterized protein	4.15	7.89E-09
SAOUHSC_00926	SAOUHSC_00926	Oligopeptide ABC transporter, ATP-binding protein, putative	2.69	1.54E-05
SAOUHSC_00927	SAOUHSC_00927	Oligopeptide ABC transporter, substrate-binding protein, putat	2.08	5.94E-04
SAOUHSC_00931	SAOUHSC_00931	Oligopeptide ABC transporter, permease protein, putative	2.28	8.44E-04
SAOUHSC_00933	trpS	Tryptophan--tRNA ligase	2.25	1.28E-11
SAOUHSC_00934	spxA	Regulatory protein Spx	-4.24	6.48E-17
SAOUHSC_00935	mecA	Adapter protein MecA	-4.15	4.84E-31
SAOUHSC_00936	SAOUHSC_00936	Putative uncharacterized protein	-2.63	6.77E-11
SAOUHSC_00937	SAOUHSC_00937	Oligoendopeptidase F	5.77	2.61E-35
SAOUHSC_00938	SAOUHSC_00938	UPF0413 protein SAOUHSC_00938	2.32	5.79E-10
SAOUHSC_00939	SAOUHSC_00939	Putative uncharacterized protein	2.98	1.83E-18
SAOUHSC_00943	ppnK	Probable inorganic polyphosphate/ATP-NAD kinase	-2.26	4.26E-06
SAOUHSC_00948	SAOUHSC_00948	Putative uncharacterized protein	3.07	5.42E-16
SAOUHSC_00949	SAOUHSC_00949	Putative uncharacterized protein	2.33	5.69E-04
SAOUHSC_00951	SAOUHSC_00951	UPF0477 protein SAOUHSC_00951	-5.42	4.75E-25
SAOUHSC_00957	SAOUHSC_00957	Putative uncharacterized protein	-2.44	2.45E-07
SAOUHSC_00958	SAOUHSC_00958	Serine protease HtrA-like	2.84	5.72E-10
SAOUHSC_00962	SAOUHSC_00962	Putative uncharacterized protein	-6.94	1.85E-22
SAOUHSC_00968	SAOUHSC_00968	Putative uncharacterized protein	3.37	5.41E-03

SAOUHSC_00975	SAOUHSC_00975	Putative uncharacterized protein	3.42	2.58E-03
SAOUHSC_00979	SAOUHSC_00979	Putative uncharacterized protein	4.80	1.37E-18
SAOUHSC_00980	SAOUHSC_00980	Putative uncharacterized protein	2.58	1.26E-09
SAOUHSC_00981	SAOUHSC_00981	Putative uncharacterized protein	2.35	3.30E-04
SAOUHSC_00985	SAOUHSC_00985	Enoyl-CoA hydratase/isomerase family protein, putative	-2.97	8.75E-19
SAOUHSC_00989	SAOUHSC_00989	Putative uncharacterized protein	4.77	7.03E-14
SAOUHSC_00991	SAOUHSC_00991	Putative uncharacterized protein	2.87	7.20E-05
SAOUHSC_00994	atl	Bifunctional autolysin	5.42	1.96E-54
SAOUHSC_00995	SAOUHSC_00995	UPF0039 protein SAOUHSC_00995	7.13	2.68E-13
SAOUHSC_00997	SAOUHSC_00997	Uncharacterized protein SAOUHSC_00997	2.33	5.51E-14
SAOUHSC_00999	qoxD	Probable quinol oxidase subunit 4	-4.64	1.09E-24
SAOUHSC_01000	qoxC	Probable quinol oxidase subunit 3	-3.86	1.51E-22
SAOUHSC_01001	qoxB	Probable quinol oxidase subunit 1	-3.05	1.04E-18
SAOUHSC_01002	qoxA	Probable quinol oxidase subunit 2	-2.54	1.51E-12
SAOUHSC_01007	folD	Bifunctional protein FolD	-3.11	1.97E-19
SAOUHSC_01008	SAOUHSC_01008	Phosphoribosylaminoimidazole carboxylase, catalytic subunit	18.54	2.93E-58
SAOUHSC_01009	SAOUHSC_01009	Phosphoribosylaminoimidazole carboxylase, ATPase subunit	28.76	5.45E-82
SAOUHSC_01010	purC	Phosphoribosylaminoimidazole-succinocarboxamide synthase	33.49	2.62E-58
SAOUHSC_01011	SAOUHSC_01011	Phosphoribosylformylglycinamidine synthase, PurS protein	28.82	3.67E-45
SAOUHSC_01012	purQ	Phosphoribosylformylglycinamidine synthase 1	22.50	2.27E-44
SAOUHSC_01013	purL	Phosphoribosylformylglycinamidine synthase 2	12.41	4.90E-33
SAOUHSC_01014	SAOUHSC_01014	Amidophosphoribosyltransferase	6.50	4.52E-22
SAOUHSC_01015	purM	Phosphoribosylformylglycinamidine cyclo-ligase	4.84	3.28E-17
SAOUHSC_01016	SAOUHSC_01016	Phosphoribosylglycinamide formyltransferase, putative	4.03	3.43E-15
SAOUHSC_01017	purH	Bifunctional purine biosynthesis protein PurH	2.23	6.92E-08
SAOUHSC_01019	SAOUHSC_01019	Putative uncharacterized protein	-2.79	2.08E-15
SAOUHSC_01021	SAOUHSC_01021	Putative uncharacterized protein	-2.17	3.89E-08
SAOUHSC_01037	SAOUHSC_01037	Putative uncharacterized protein	3.46	2.23E-25
SAOUHSC_01040	SAOUHSC_01040	Pyruvate dehydrogenase complex, E1 component, alpha subun	-3.78	4.59E-27
SAOUHSC_01041	SAOUHSC_01041	Pyruvate dehydrogenase complex, E1 component, pyruvate de	-5.18	4.83E-39
SAOUHSC_01042	SAOUHSC_01042	Dihydrolipoamide S-acetyltransferase component of pyruvate c	-6.50	3.94E-50
SAOUHSC_01043	SAOUHSC_01043	Dihydrolipoyl dehydrogenase	-10.69	1.02E-64
SAOUHSC_01044	SAOUHSC_01044	UPF0223 protein SAOUHSC_01044	-2.76	5.62E-12
SAOUHSC_01051	SAOUHSC_01051	Putative uncharacterized protein	2.20	3.99E-06
SAOUHSC_01054	SAOUHSC_01054	UPF0637 protein SAOUHSC_01054	3.85	2.16E-14
SAOUHSC_01055	SAOUHSC_01055	Inositol monophosphatase family protein, putative	-6.79	5.50E-46
SAOUHSC_01057	SAOUHSC_01057	Putative uncharacterized protein	-2.30	2.84E-08
SAOUHSC_01060	SAOUHSC_01060	Putative uncharacterized protein	2.32	7.70E-07
SAOUHSC_01061	SAOUHSC_01061	Putative uncharacterized protein	2.45	8.12E-13
SAOUHSC_01062	SAOUHSC_01062	UPF0358 protein SAOUHSC_01062	2.64	3.75E-20
SAOUHSC_01063	SAOUHSC_01063	Putative uncharacterized protein	3.35	9.64E-14
SAOUHSC_01064	SAOUHSC_01064	Pyruvate carboxylase	2.42	5.65E-10
SAOUHSC_01069	SAOUHSC_01069	Putative uncharacterized protein	2.19	3.70E-11
SAOUHSC_01072	SAOUHSC_01072	UPF0298 protein SAOUHSC_01072	2.50	8.92E-07
SAOUHSC_01077	SAOUHSC_01077	Putative uncharacterized protein	-2.08	3.90E-08
SAOUHSC_01081	isdA	Iron-regulated surface determinant protein A	5.87	3.66E-09
SAOUHSC_01084	SAOUHSC_01084	Putative uncharacterized protein	2.48	7.21E-03
SAOUHSC_01092	pheS	Phenylalanine--tRNA ligase alpha subunit	-2.04	1.50E-06
SAOUHSC_01093	pheT	Phenylalanine--tRNA ligase beta subunit	-3.45	5.11E-14
SAOUHSC_01095	rnhC	Ribonuclease HIII	3.05	5.62E-08
SAOUHSC_01101	SAOUHSC_01101	Putative uncharacterized protein	2.34	4.18E-09
SAOUHSC_01103	SAOUHSC_01103	Succinate dehydrogenase cytochrome b-558 subunit, putative	-4.65	5.31E-21
SAOUHSC_01104	SAOUHSC_01104	Succinate dehydrogenase, flavoprotein chain TC0881, putative	-6.52	1.80E-31
SAOUHSC_01105	SAOUHSC_01105	Iron-sulphur subunit of succinate dehydrogenase, putative	-7.62	7.09E-37
SAOUHSC_01110	SAOUHSC_01110	Fibrinogen-binding protein-related	-2.23	6.12E-06
SAOUHSC_01123	SAOUHSC_01123	Putative uncharacterized protein	-2.28	1.69E-05
SAOUHSC_01124	SAOUHSC_01124	Putative uncharacterized protein	3.63	1.11E-03
SAOUHSC_01125	SAOUHSC_01125	Putative uncharacterized protein	7.28	6.55E-09
SAOUHSC_01127	SAOUHSC_01127	Putative uncharacterized protein	6.04	5.46E-19
SAOUHSC_01133	SAOUHSC_01133	Putative uncharacterized protein	2.59	6.78E-13
SAOUHSC_01134	SAOUHSC_01134	Putative uncharacterized protein	4.23	1.90E-12
SAOUHSC_01135	SAOUHSC_01135	Putative uncharacterized protein	-3.05	1.77E-15
SAOUHSC_01136	SAOUHSC_01136	Putative uncharacterized protein	-3.54	1.06E-25
SAOUHSC_01138	SAOUHSC_01138	Uncharacterized N-acetyltransferase SAOUHSC_01138	-3.94	3.63E-22
SAOUHSC_01160	SAOUHSC_01160	Putative uncharacterized protein	2.36	8.57E-13
SAOUHSC_01161	SAOUHSC_01161	Truncated transposase	2.81	9.50E-08
SAOUHSC_01163	SAOUHSC_01163	Pseudouridine synthase	-2.07	1.36E-09
SAOUHSC_01164	pyrR	Bifunctional protein PyrR	-2.25	1.97E-08
SAOUHSC_01165	SAOUHSC_01165	Uracil permease, putative	-4.58	7.74E-04
SAOUHSC_01166	pyrB	Aspartate carbamoyltransferase	-6.22	1.68E-14
SAOUHSC_01168	pyrC	Dihydroorotase	-4.79	4.95E-14
SAOUHSC_01169	carA	Carbamoyl-phosphate synthase small chain	-4.56	7.27E-17
SAOUHSC_01170	carB	Carbamoyl-phosphate synthase large chain	-4.01	3.27E-20
SAOUHSC_01171	pyrF	Orotidine 5'-phosphate decarboxylase	-3.86	2.99E-15
SAOUHSC_01172	pyrE	Orotate phosphoribosyltransferase	-3.95	2.47E-21
SAOUHSC_01175	SAOUHSC_01175	Fibronectin-binding protein A-related	2.14	3.63E-07
SAOUHSC_01176	gmk	Guanylate kinase	2.99	5.70E-23
SAOUHSC_01180	SAOUHSC_01180	Putative uncharacterized protein	2.33	3.80E-08
SAOUHSC_01181	SAOUHSC_01181	Putative uncharacterized protein	-2.56	4.12E-15
SAOUHSC_01182			2.25	8.16E-11
SAOUHSC_01184	SAOUHSC_01184	Sun protein	2.09	5.24E-11
SAOUHSC_01186	SAOUHSC_01186	Putative uncharacterized protein	2.44	4.27E-14
SAOUHSC_01187	SAOUHSC_01187	Putative uncharacterized protein	2.00	9.99E-11
SAOUHSC_01189	SAOUHSC_01189	Ribulose-phosphate 3-epimerase	-2.70	6.25E-23
SAOUHSC_01190	SAOUHSC_01190	Putative uncharacterized protein	-2.92	1.59E-21
SAOUHSC_01191	rpmB	50S ribosomal protein L28	-6.40	3.61E-41

SAOUHSC_01198	SAOUHSC_01198	Malonyl CoA-acyl carrier protein transacylase	-2.21	1.11E-07
SAOUHSC_01199	SAOUHSC_01199	3-oxoacyl-(Acyl-carrier-protein) reductase, putative	-2.35	2.78E-09
SAOUHSC_01207	ffh	Signal recognition particle protein	-2.50	1.45E-24
SAOUHSC_01210	trmD	tRNA (guanine-N(1)-)-methyltransferase	-2.95	6.72E-09
SAOUHSC_01214	SAOUHSC_01214	Ribosome biogenesis GTPase A	-3.10	1.38E-15
SAOUHSC_01215	rnhB	Ribonuclease HII	-4.25	3.06E-24
SAOUHSC_01216	sucC	Succinyl-CoA ligase [ADP-forming] subunit beta	-2.80	9.38E-22
SAOUHSC_01218	SAOUHSC_01218	Succinyl-CoA ligase [ADP-forming] subunit alpha	-2.91	2.07E-19
SAOUHSC_01221	SAOUHSC_01221	Putative uncharacterized protein	2.28	1.06E-03
SAOUHSC_01242	rimP	Ribosome maturation factor RimP	-3.07	1.57E-17
SAOUHSC_01243	nusA	Transcription termination/antitermination protein NusA	-4.99	1.04E-30
SAOUHSC_01244	SAOUHSC_01244	Putative uncharacterized protein	-6.44	2.34E-40
SAOUHSC_01245	SAOUHSC_01245	Putative uncharacterized protein	-5.12	2.65E-36
SAOUHSC_01246	infB	Translation initiation factor IF-2	-11.77	1.67E-49
SAOUHSC_01247	rbfA	Ribosome-binding factor A	-4.19	1.31E-26
SAOUHSC_01248	truB	tRNA pseudouridine synthase B	3.19	1.53E-09
SAOUHSC_01249	SAOUHSC_01249	Riboflavin biosynthesis protein RibF	2.61	2.61E-20
SAOUHSC_01255	SAOUHSC_01255	Putative uncharacterized protein	3.13	1.05E-06
SAOUHSC_01257	SAOUHSC_01257	Putative uncharacterized protein	-2.23	1.72E-08
SAOUHSC_01263	rny	Ribonuclease Y	-3.47	5.86E-29
SAOUHSC_01264	SAOUHSC_01264	Putative uncharacterized protein	4.26	1.07E-23
SAOUHSC_01266	SAOUHSC_01266	Putative uncharacterized protein	-3.58	5.62E-19
SAOUHSC_01267	SAOUHSC_01267	Putative uncharacterized protein	-4.12	1.64E-24
SAOUHSC_01269	miaB	(Dimethylallyl)adenosine tRNA methylthiotransferase MiaB	2.99	2.32E-22
SAOUHSC_01270	SAOUHSC_01270	Putative uncharacterized protein	2.88	1.18E-21
SAOUHSC_01271	SAOUHSC_01271	Putative uncharacterized protein	2.31	1.34E-13
SAOUHSC_01272	mutS	DNA mismatch repair protein MutS	3.70	3.87E-40
SAOUHSC_01273	mutL	DNA mismatch repair protein MutL	3.81	3.28E-26
SAOUHSC_01274	SAOUHSC_01274	Glycerol uptake operon antiterminator regulatory protein, puta	2.67	1.50E-11
SAOUHSC_01276	glpK	Glycerol kinase	-2.56	1.48E-08
SAOUHSC_01282	SAOUHSC_01282	Glutathione peroxidase	2.14	1.08E-10
SAOUHSC_01285	SAOUHSC_01285	Putative uncharacterized protein	3.25	8.02E-15
SAOUHSC_01295	SAOUHSC_01295	Putative uncharacterized protein	-2.84	8.78E-03
SAOUHSC_01296	SAOUHSC_01296	Putative uncharacterized protein	-7.36	2.32E-04
SAOUHSC_01304	SAOUHSC_01304	Putative uncharacterized protein	-4.26	5.41E-27
SAOUHSC_01305	SAOUHSC_01305	Putative uncharacterized protein	-3.59	2.56E-07
SAOUHSC_01310			2.46	1.51E-12
SAOUHSC_01316	SAOUHSC_01316	Putative uncharacterized protein	2.29	7.01E-04
SAOUHSC_01320	SAOUHSC_01320	Homoserine dehydrogenase	3.25	1.43E-07
SAOUHSC_01323	SAOUHSC_01323	Putative uncharacterized protein	-2.90	1.40E-11
SAOUHSC_01324	SAOUHSC_01324	Putative uncharacterized protein	-5.51	1.46E-13
SAOUHSC_01325	SAOUHSC_01325	Putative uncharacterized protein	-4.98	5.47E-13
SAOUHSC_01326	SAOUHSC_01326	Putative uncharacterized protein	2.09	4.28E-06
SAOUHSC_01330	guaC	GMP reductase	3.59	5.25E-21
SAOUHSC_01331	SAOUHSC_01331	Putative uncharacterized protein	3.81	2.71E-05
SAOUHSC_01332	SAOUHSC_01332	Putative uncharacterized protein	2.61	7.95E-18
SAOUHSC_01333	lexA	LexA repressor	-2.31	4.41E-06
SAOUHSC_01336	SAOUHSC_01336	UPF0291 protein SAOUHSC_01336	4.18	8.82E-18
SAOUHSC_01340	SAOUHSC_01340	Putative uncharacterized protein	2.37	1.67E-09
SAOUHSC_01344	SAOUHSC_01344	Putative uncharacterized protein	3.09	1.02E-26
SAOUHSC_01347	SAOUHSC_01347	Aconitate hydratase 1	-3.42	1.60E-17
SAOUHSC_01351	parE	DNA topoisomerase 4 subunit B	2.77	4.04E-15
SAOUHSC_01353	SAOUHSC_01353	Putative uncharacterized protein	2.47	1.00E-04
SAOUHSC_01356	glcT	Protein GlcT	3.34	5.86E-20
SAOUHSC_01357	SAOUHSC_01357	Putative uncharacterized protein	4.12	1.69E-10
SAOUHSC_01358	SAOUHSC_01358	Putative uncharacterized protein	2.45	2.88E-18
SAOUHSC_01366	SAOUHSC_01366	Anthranilate synthase component I	4.73	4.30E-04
SAOUHSC_01370	trpF	N-(5'-phosphoribosyl)anthranilate isomerase	4.10	7.66E-03
SAOUHSC_01372	trpA	Tryptophan synthase alpha chain	-2.41	5.66E-08
SAOUHSC_01373	femA	Aminoacyltransferase FemA	3.86	2.44E-20
SAOUHSC_01374	femB	Aminoacyltransferase FemB	2.26	4.33E-11
SAOUHSC_01375	SAOUHSC_01375	Putative uncharacterized protein	7.03	6.91E-19
SAOUHSC_01376	SAOUHSC_01376	Putative uncharacterized protein	-2.19	5.93E-12
SAOUHSC_01377	oppF2	Putative oligopeptide transport ATP-binding protein oppF2	-3.08	1.03E-22
SAOUHSC_01378	oppD2	Putative oligopeptide transport ATP-binding protein oppD2	-2.49	6.62E-13
SAOUHSC_01379	oppC2	Putative oligopeptide transport system permease protein oppC	-3.27	2.10E-18
SAOUHSC_01380	oppB2	Putative oligopeptide transport system permease protein oppB	-3.15	1.34E-14
SAOUHSC_01381	SAOUHSC_01381	Putative uncharacterized protein	-2.53	5.37E-05
SAOUHSC_01383	SAOUHSC_01383	Putative uncharacterized protein	6.93	7.10E-37
SAOUHSC_01394	SAOUHSC_01394	Aspartokinase	3.77	4.69E-06
SAOUHSC_01395	asd	Aspartate-semialdehyde dehydrogenase	4.35	5.76E-07
SAOUHSC_01396	dapA	4-hydroxy-tetrahydrodipicolinate synthase	4.40	7.12E-07
SAOUHSC_01397	dapB	4-hydroxy-tetrahydrodipicolinate reductase	2.81	8.50E-06
SAOUHSC_01399	SAOUHSC_01399	Uncharacterized hydrolase SAOUHSC_01399	2.19	7.31E-06
SAOUHSC_01402	msa	Protein msa	-3.14	1.28E-03
SAOUHSC_01403	cspA	Cold shock protein CspA	-2.45	7.07E-16
SAOUHSC_01404	SAOUHSC_01404	Putative uncharacterized protein	-2.21	3.58E-14
SAOUHSC_01405	SAOUHSC_01405	Putative uncharacterized protein	2.48	6.86E-08
SAOUHSC_01408	SAOUHSC_01408	TelA-like protein SAOUHSC_01408	-2.02	8.12E-06
SAOUHSC_01412	SAOUHSC_01412	Putative uncharacterized protein	-2.47	5.39E-17
SAOUHSC_01414	SAOUHSC_01414	Putative uncharacterized protein	-5.12	4.10E-21
SAOUHSC_01415	SAOUHSC_01415	Putative uncharacterized protein	-4.24	1.24E-18
SAOUHSC_01416	odhB	Dihydrolipoyllysine-residue succinyltransferase component of 2	-4.55	3.89E-45
SAOUHSC_01418	odhA	2-oxoglutarate dehydrogenase E1 component	-3.67	1.25E-34
SAOUHSC_01427	SAOUHSC_01427	Putative uncharacterized protein	3.18	2.07E-19
SAOUHSC_01428	SAOUHSC_01428	Putative uncharacterized protein	5.34	2.76E-08

SAOUHSC_01429	SAOUHSC_01429	UPF0346 protein SAOUHSC_01429	-4.54	1.23E-25
SAOUHSC_01430	SAOUHSC_01430	Phosphotransferase system enzyme IIA, putative	-4.43	1.45E-22
SAOUHSC_01431	msrB	Peptide methionine sulfoxide reductase MsrB	-3.37	9.67E-20
SAOUHSC_01432	msrA2	Peptide methionine sulfoxide reductase MsrA 2	-3.04	4.16E-16
SAOUHSC_01435	thyA	Thymidylate synthase	2.84	2.45E-06
SAOUHSC_01438	SAOUHSC_01438	Putative uncharacterized protein	2.19	4.38E-08
SAOUHSC_01439	SAOUHSC_01439	Putative uncharacterized protein	3.53	3.60E-17
SAOUHSC_01440	SAOUHSC_01440	Putative uncharacterized protein	2.36	1.58E-05
SAOUHSC_01443	SAOUHSC_01443	Putative uncharacterized protein	3.89	2.63E-04
SAOUHSC_01447	ebh	Extracellular matrix-binding protein ebh	-5.16	9.29E-42
SAOUHSC_01448	norB	Quinolone resistance protein NorB	2.44	1.77E-03
SAOUHSC_01450	SAOUHSC_01450	Putative uncharacterized protein	3.20	3.51E-11
SAOUHSC_01451	tdcB	L-threonine dehydratase catabolic TdcB	2.62	9.20E-09
SAOUHSC_01452	ald1	Alanine dehydrogenase 1	10.65	1.77E-42
SAOUHSC_01455	SAOUHSC_01455	Putative uncharacterized protein	2.65	4.43E-23
SAOUHSC_01460	SAOUHSC_01460	Putative uncharacterized protein	2.22	8.53E-08
SAOUHSC_01464	SAOUHSC_01464	Putative uncharacterized protein	2.02	3.15E-13
SAOUHSC_01466	recU	Holliday junction resolvase RecU	-3.02	5.02E-15
SAOUHSC_01467	SAOUHSC_01467	Penicillin-binding protein 2	-3.23	1.50E-18
SAOUHSC_01468	SAOUHSC_01468	Putative uncharacterized protein	-2.86	9.33E-11
SAOUHSC_01469	nth	Endonuclease III	-2.81	2.39E-11
SAOUHSC_01477	SAOUHSC_01477	Putative uncharacterized protein	-2.90	3.49E-12
SAOUHSC_01479	SAOUHSC_01479	UPF0302 protein SAOUHSC_01479	2.12	2.06E-06
SAOUHSC_01485	ndk	Nucleoside diphosphate kinase	2.28	1.59E-05
SAOUHSC_01486	SAOUHSC_01486	Heptaprenyl diphosphate syntase component II, putative	3.95	2.03E-31
SAOUHSC_01487	SAOUHSC_01487	Menaquinone biosynthesis methyltransferase, putative	2.93	7.96E-21
SAOUHSC_01488	SAOUHSC_01488	Putative uncharacterized protein	3.62	3.73E-21
SAOUHSC_01489	SAOUHSC_01489	Putative uncharacterized protein	3.57	6.75E-13
SAOUHSC_01490	SAOUHSC_01490	DNA-binding protein HU, putative	2.11	4.75E-10
SAOUHSC_01492	der	GTPase Der	2.33	2.42E-11
SAOUHSC_01496	SAOUHSC_01496	Cytidylate kinase	3.68	1.16E-17
SAOUHSC_01502	SAOUHSC_01502	ATP-dependent DNA helicase RecQ, putative	3.47	2.47E-08
SAOUHSC_01503	SAOUHSC_01503	Putative uncharacterized protein	2.81	2.00E-11
SAOUHSC_01504	SAOUHSC_01504	Ferredoxin, putative	-3.37	1.08E-20
SAOUHSC_01513	SAOUHSC_01513	Putative uncharacterized protein	-3.24	1.08E-12
SAOUHSC_01575	SAOUHSC_01575	Helix-turn-helix domain protein	-2.28	1.36E-08
SAOUHSC_01585	srrB	Sensor protein SrrB	-3.29	5.11E-19
SAOUHSC_01586	srrA	Transcriptional regulatory protein SrrA	-5.06	4.42E-22
SAOUHSC_01590	SAOUHSC_01590	Putative uncharacterized protein	2.52	8.68E-07
SAOUHSC_01591	xerD	Tyrosine recombinase XerD	-2.44	1.34E-11
SAOUHSC_01593	SAOUHSC_01593	NUDIX domain protein	2.70	1.08E-09
SAOUHSC_01594	SAOUHSC_01594	Putative uncharacterized protein	3.98	1.77E-42
SAOUHSC_01595	SAOUHSC_01595	Putative uncharacterized protein	4.01	1.49E-10
SAOUHSC_01596	SAOUHSC_01596	Putative uncharacterized protein	3.31	7.56E-16
SAOUHSC_01597	SAOUHSC_01597	Pyrroline-5-carboxylate reductase	2.91	3.15E-20
SAOUHSC_01598	rnz	Ribonuclease Z	2.23	4.56E-10
SAOUHSC_01599	zwf	Glucose-6-phosphate 1-dehydrogenase	2.40	6.53E-16
SAOUHSC_01601	SAOUHSC_01601	Alpha-glucosidase, putative	-2.76	7.18E-19
SAOUHSC_01604	SAOUHSC_01604	Putative uncharacterized protein	3.83	4.17E-11
SAOUHSC_01610	SAOUHSC_01610	UPF0403 protein SAOUHSC_01610	2.53	5.36E-18
SAOUHSC_01611	SAOUHSC_01611	2-oxoisovalerate dehydrogenase, E2 component, dihydrolipoar	-3.18	2.20E-22
SAOUHSC_01612	SAOUHSC_01612	2-oxoisovalerate dehydrogenase, E1 component, beta subunit,	-3.14	8.61E-21
SAOUHSC_01613	SAOUHSC_01613	2-oxoisovalerate dehydrogenase, E1 component, alpha subunit	-2.90	4.47E-20
SAOUHSC_01618	SAOUHSC_01618	Geranyltranstransferase, putative	2.63	1.80E-13
SAOUHSC_01619	xseB	Exodeoxyribonuclease 7 small subunit	2.26	2.75E-08
SAOUHSC_01622	SAOUHSC_01622	Putative uncharacterized protein	2.43	5.19E-13
SAOUHSC_01623	SAOUHSC_01623	Acetyl-CoA carboxylase, biotin carboxylase	2.39	9.11E-22
SAOUHSC_01624	SAOUHSC_01624	Acetyl-CoA carboxylase, biotin carboxyl carrier protein	2.77	6.64E-24
SAOUHSC_01626	SAOUHSC_01626	Proline dipeptidase, putative	3.41	2.62E-20
SAOUHSC_01627	SAOUHSC_01627	Putative uncharacterized protein	2.39	1.03E-11
SAOUHSC_01628	SAOUHSC_01628	Putative uncharacterized protein	2.10	7.72E-04
SAOUHSC_01632	gcvPB	Probable glycine dehydrogenase (decarboxylating) subunit 2	-2.53	7.58E-15
SAOUHSC_01633	gcvPA	Probable glycine dehydrogenase (decarboxylating) subunit 1	-2.14	9.35E-13
SAOUHSC_01644	SAOUHSC_01644	Putative uncharacterized protein	-3.27	1.44E-23
SAOUHSC_01645	SAOUHSC_01645	Putative uncharacterized protein	-2.79	3.17E-24
SAOUHSC_01646	SAOUHSC_01646	Glucokinase, putative	-2.61	2.14E-19
SAOUHSC_01652	SAOUHSC_01652	Penicillin-binding protein 3	2.14	5.91E-12
SAOUHSC_01655	fur	Ferric uptake regulation protein	-2.75	3.27E-14
SAOUHSC_01656	SAOUHSC_01656	Putative uncharacterized protein	-2.47	3.75E-10
SAOUHSC_01660	SAOUHSC_01660	Putative GTP cyclohydrolase 1 type 2	-2.04	3.24E-10
SAOUHSC_01662	sigA	RNA polymerase sigma factor SigA	-2.42	2.64E-15
SAOUHSC_01664	SAOUHSC_01664	Putative pyruvate, phosphate dikinase regulatory protein	-2.05	7.81E-09
SAOUHSC_01667	recO	DNA repair protein RecO	-3.63	1.56E-20
SAOUHSC_01668	era	GTPase Era	-2.96	1.35E-21
SAOUHSC_01669	SAOUHSC_01669	Putative uncharacterized protein	-2.55	5.19E-15
SAOUHSC_01670	SAOUHSC_01670	Cytidine deaminase	-2.87	1.19E-15
SAOUHSC_01675	SAOUHSC_01675	Putative uncharacterized protein	-4.51	3.47E-26
SAOUHSC_01676	SAOUHSC_01676	UPF0365 protein SAOUHSC_01676	-3.57	1.73E-22
SAOUHSC_01677	SAOUHSC_01677	Putative uncharacterized protein	-2.28	9.56E-10
SAOUHSC_01681	prmA	Ribosomal protein L11 methyltransferase	-13.21	1.35E-51
SAOUHSC_01682	dnaJ	Chaperone protein DnaJ	-10.77	2.58E-54
SAOUHSC_01683	dnaK	Chaperone protein DnaK	-12.28	4.54E-49
SAOUHSC_01684	grpE	Protein GrpE	-3.99	3.55E-18
SAOUHSC_01685	hrcA	Heat-inducible transcription repressor HrcA	-4.30	1.01E-24
SAOUHSC_01691	SAOUHSC_01691	DNA internalization-related competence protein ComEC/Rec2	2.36	7.53E-06
SAOUHSC_01707	SAOUHSC_01707	Putative uncharacterized protein	-3.57	2.17E-16

SAOUHSC_01708	SAOUHSC_01708	UPF0271 protein SAOUHSC_01708	-3.07	1.98E-14
SAOUHSC_01709	SAOUHSC_01709	Acetyl-CoA carboxylase, biotin carboxylase, putative	-2.64	5.73E-12
SAOUHSC_01714	greA	Transcription elongation factor GreA	-2.36	1.47E-12
SAOUHSC_01715	udk	Uridine kinase	-2.38	3.77E-17
SAOUHSC_01716	SAOUHSC_01716	Putative uncharacterized protein	-2.69	2.33E-20
SAOUHSC_01725			-2.62	1.20E-14
SAOUHSC_01730	SAOUHSC_01730	UPF0337 protein SAOUHSC_01730	2.61	4.70E-05
SAOUHSC_01732	SAOUHSC_01732	Putative uncharacterized protein	4.06	2.25E-20
SAOUHSC_01733	SAOUHSC_01733	Putative uncharacterized protein	3.29	5.74E-09
SAOUHSC_01737	aspS	Aspartate--tRNA ligase	-2.95	1.54E-18
SAOUHSC_01739	lytH	Probable cell wall amidase LytH	5.61	1.18E-40
SAOUHSC_01741	dtd	D-tyrosyl-tRNA(Tyr) deacylase	4.93	7.33E-43
SAOUHSC_01742	SAOUHSC_01742	GTP pyrophosphokinase	4.91	1.40E-49
SAOUHSC_01746	SAOUHSC_01746	Protein-export membrane protein SecDF	2.49	2.12E-14
SAOUHSC_01755	rpmA	50S ribosomal protein L27	-3.58	8.26E-21
SAOUHSC_01756	SAOUHSC_01756	Putative uncharacterized protein	-2.34	6.41E-16
SAOUHSC_01760	SAOUHSC_01760	Putative uncharacterized protein	-4.93	1.19E-30
SAOUHSC_01761	SAOUHSC_01761	Putative uncharacterized protein	-3.83	5.00E-25
SAOUHSC_01766	SAOUHSC_01766	Folylpolyglutamate synthase/dihydrofolate synthase, putative	-5.21	3.88E-37
SAOUHSC_01767	valS	Valine--tRNA ligase	-3.46	3.21E-23
SAOUHSC_01771	hemL1	Glutamate-1-semialdehyde 2,1-aminomutase 1	-2.90	1.17E-22
SAOUHSC_01772	hemB	Delta-aminolevulinic acid dehydratase	-2.71	6.78E-21
SAOUHSC_01776	hemA	Glutamyl-tRNA reductase	-2.06	9.18E-07
SAOUHSC_01778	clpX	ATP-dependent Clp protease ATP-binding subunit ClpX	-2.72	4.06E-15
SAOUHSC_01781	SAOUHSC_01781	Putative uncharacterized protein	2.56	3.21E-12
SAOUHSC_01782	SAOUHSC_01782	Putative uncharacterized protein	2.95	1.51E-17
SAOUHSC_01784	rplT	50S ribosomal protein L20	-2.31	2.41E-11
SAOUHSC_01793	nrdR	Transcriptional repressor NrdR	2.28	3.03E-07
SAOUHSC_01801	SAOUHSC_01801	Isocitrate dehydrogenase [NADP]	-6.73	2.07E-44
SAOUHSC_01802	SAOUHSC_01802	Putative uncharacterized protein	-5.22	6.25E-42
SAOUHSC_01803	SAOUHSC_01803	Putative uncharacterized protein	4.77	1.37E-23
SAOUHSC_01804	SAOUHSC_01804	Transposase, putative	5.61	2.09E-04
SAOUHSC_01806	pyk	Pyruvate kinase	-4.16	1.95E-22
SAOUHSC_01807	pfkA	6-phosphofructokinase	-3.01	2.90E-10
SAOUHSC_01808	accA	Acetyl-coenzyme A carboxylase carboxyl transferase subunit al	-3.16	6.05E-17
SAOUHSC_01809	accD	Acetyl-coenzyme A carboxylase carboxyl transferase subunit be	-3.16	9.53E-17
SAOUHSC_01810	SAOUHSC_01810	NADP-dependent malic enzyme, putative	-2.28	2.26E-14
SAOUHSC_01814	SAOUHSC_01814	Putative uncharacterized protein	-3.30	2.44E-14
SAOUHSC_01816	SAOUHSC_01816	Uncharacterized peptidase SAOUHSC_01816	4.87	2.45E-41
SAOUHSC_01817	SAOUHSC_01817	Putative uncharacterized protein	4.67	2.44E-14
SAOUHSC_01818	ald2	Alanine dehydrogenase 2	-2.89	1.60E-25
SAOUHSC_01819	SAOUHSC_01819	Putative universal stress protein SAOUHSC_01819	-21.58	2.21E-47
SAOUHSC_01821	SAOUHSC_01821	Putative uncharacterized protein	2.69	1.56E-17
SAOUHSC_01827	ezrA	Septation ring formation regulator EzrA	-2.54	9.35E-09
SAOUHSC_01830	SAOUHSC_01830	Putative uncharacterized protein	2.10	3.40E-07
SAOUHSC_01832	SAOUHSC_01832	Putative uncharacterized protein	4.12	7.86E-10
SAOUHSC_01833	SAOUHSC_01833	D-3-phosphoglycerate dehydrogenase	2.99	1.29E-06
SAOUHSC_01837	SAOUHSC_01837	1-acyl-sn-glycerol-3-phosphate acyltransferases domain protei	2.26	4.50E-12
SAOUHSC_01838	SAOUHSC_01838	Putative uncharacterized protein	2.71	7.18E-24
SAOUHSC_01839	tyrS	Tyrosine--tRNA ligase	2.95	8.78E-10
SAOUHSC_01840	SAOUHSC_01840	Transglycosylase domain protein	3.38	3.96E-12
SAOUHSC_01841	SAOUHSC_01841	Putative uncharacterized protein	2.46	6.39E-03
SAOUHSC_01843	isdH	Iron-regulated surface determinant protein H	-2.29	4.63E-13
SAOUHSC_01846	SAOUHSC_01846	Acetyl-CoA synthetase, putative	-2.35	5.67E-09
SAOUHSC_01847	SAOUHSC_01847	Putative uncharacterized protein	3.34	2.43E-13
SAOUHSC_01849	SAOUHSC_01849	Putative uncharacterized protein	3.33	3.00E-18
SAOUHSC_01850	SAOUHSC_01850	Catabolite control protein A	-2.43	9.94E-23
SAOUHSC_01854	SAOUHSC_01854	Putative uncharacterized protein	-2.37	3.03E-12
SAOUHSC_01860	SAOUHSC_01860	Putative uncharacterized protein	-3.71	1.99E-28
SAOUHSC_01861	SAOUHSC_01861	Putative uncharacterized protein	-3.16	2.91E-16
SAOUHSC_01865	trmB	tRNA (guanine-N(7)-)-methyltransferase	-2.24	1.45E-07
SAOUHSC_01866	SAOUHSC_01866	Putative uncharacterized protein	-2.87	1.23E-10
SAOUHSC_01867	SAOUHSC_01867	D-alanine aminotransferase	2.07	2.13E-10
SAOUHSC_01868	SAOUHSC_01868	Putative dipeptidase SAOUHSC_01868	5.31	2.08E-52
SAOUHSC_01873	SAOUHSC_01873	Putative uncharacterized protein	-4.93	4.41E-31
SAOUHSC_01877	SAOUHSC_01877	Putative uncharacterized protein	2.40	7.64E-15
SAOUHSC_01879	rot	HTH-type transcriptional regulator rot	-5.48	8.27E-29
SAOUHSC_01884	SAOUHSC_01884	Putative uncharacterized protein	-2.73	9.99E-06
SAOUHSC_01886	ribH	6,7-dimethyl-8-ribityllumazine synthase	-10.04	4.00E-41
SAOUHSC_01887	ribBA	Riboflavin biosynthesis protein RibBA	-7.35	1.37E-28
SAOUHSC_01888	SAOUHSC_01888	Riboflavin synthase, alpha subunit	-5.78	7.98E-19
SAOUHSC_01889	SAOUHSC_01889	Riboflavin biosynthesis protein RibD	-4.03	2.93E-15
SAOUHSC_01890	SAOUHSC_01890	Putative uncharacterized protein	3.06	1.55E-08
SAOUHSC_01895	SAOUHSC_01895	Putative uncharacterized protein	4.00	1.82E-38
SAOUHSC_01896	SAOUHSC_01896	Putative uncharacterized protein	3.52	2.25E-20
SAOUHSC_01898	SAOUHSC_01898	Putative uncharacterized protein	4.07	3.95E-08
SAOUHSC_01899	SAOUHSC_01899	Putative uncharacterized protein	6.24	2.57E-11
SAOUHSC_01901	SAOUHSC_01901	Putative uncharacterized protein	2.03	7.50E-13
SAOUHSC_01902	SAOUHSC_01902	Putative uncharacterized protein	3.18	1.94E-03
SAOUHSC_01903	crcB1	Putative fluoride ion transporter CrcB 1	6.79	2.64E-10
SAOUHSC_01904	crcB2	Putative fluoride ion transporter CrcB 2	3.31	1.03E-05
SAOUHSC_01907	SAOUHSC_01907	Putative uncharacterized protein	2.20	3.48E-13
SAOUHSC_01915	SAOUHSC_01915	Putative uncharacterized protein	-2.89	2.41E-25
SAOUHSC_01917	SAOUHSC_01917	Putative uncharacterized protein	2.74	5.81E-04
SAOUHSC_01924	SAOUHSC_01924	Putative uncharacterized protein	-2.41	1.55E-06
SAOUHSC_01925	SAOUHSC_01925	Putative uncharacterized protein	-2.05	4.74E-05

SAOUHSC_01929	SAOUHSC_01929	Putative uncharacterized protein	-2.90	1.28E-05
SAOUHSC_01933	SAOUHSC_01933	Type I restriction-modification system, M subunit	2.36	1.24E-11
SAOUHSC_01945	SAOUHSC_01945	Membrane protein, putative	5.52	1.50E-05
SAOUHSC_01957	SAOUHSC_01957	Putative uncharacterized protein	-2.96	1.44E-06
SAOUHSC_01958	SAOUHSC_01958	Putative uncharacterized protein	3.60	7.49E-17
SAOUHSC_01959	SAOUHSC_01959	Putative uncharacterized protein	-3.10	1.19E-21
SAOUHSC_01960	SAOUHSC_01960	Protoporphyrinogen oxidase	-3.28	1.12E-21
SAOUHSC_01961	hemH	Ferrochelatase	-2.32	9.47E-15
SAOUHSC_01966	SAOUHSC_01966	Putative uncharacterized protein	2.54	3.57E-13
SAOUHSC_01967	SAOUHSC_01967	ABC transporter, ATP-binding protein, putative	4.13	3.50E-28
SAOUHSC_01971	SAOUHSC_01971	Putative uncharacterized protein	2.63	2.58E-03
SAOUHSC_01972	prsA	Foldase protein PrsA	-3.26	1.22E-15
SAOUHSC_01974	SAOUHSC_01974	Putative uncharacterized protein	2.49	3.64E-20
SAOUHSC_01975	SAOUHSC_01975	Putative uncharacterized protein	3.59	1.98E-20
SAOUHSC_01979	SAOUHSC_01979	Putative uncharacterized protein	5.20	3.59E-17
SAOUHSC_01980	SAOUHSC_01980	DNA-binding response regulator, putative	3.25	1.70E-12
SAOUHSC_01981	SAOUHSC_01981	Sensor histidine kinase, putative	2.05	3.58E-09
SAOUHSC_01983	fumC	Fumarate hydratase class II	-2.72	9.99E-14
SAOUHSC_01986	SAOUHSC_01986	Putative uncharacterized protein	-2.40	2.69E-08
SAOUHSC_01997	perR	Peroxide-responsive repressor PerR	-3.54	7.07E-13
SAOUHSC_01999	SAOUHSC_01999	Putative uncharacterized protein	2.28	9.62E-09
SAOUHSC_02002	SAOUHSC_02002	Putative uncharacterized protein	-2.03	1.49E-03
SAOUHSC_02003	SAOUHSC_02003	Putative multidrug export ATP-binding/permease protein SAOU	-2.61	2.19E-11
SAOUHSC_02004	SAOUHSC_02004	UPF0374 protein SAOUHSC_02004	-2.01	8.51E-05
SAOUHSC_02008	SAOUHSC_02008	Putative uncharacterized protein	2.58	2.99E-11
SAOUHSC_02009	SAOUHSC_02009	Putative uncharacterized protein	3.28	1.94E-26
SAOUHSC_02010	SAOUHSC_02010	Putative uncharacterized protein	3.56	1.26E-16
SAOUHSC_02011	recX	Regulatory protein RecX	3.92	7.45E-28
SAOUHSC_02090	SAOUHSC_02090	Conserved hypothetical phage protein	-2.25	1.30E-05
SAOUHSC_02093	SAOUHSC_02093	UPF0435 protein SAOUHSC_02093	3.04	1.41E-12
SAOUHSC_02098	SAOUHSC_02098	DNA-binding response regulator VraR, putative	-5.89	3.74E-32
SAOUHSC_02099	SAOUHSC_02099	Histidine kinase, putative	-4.76	1.78E-30
SAOUHSC_02100	SAOUHSC_02100	Putative uncharacterized protein	-4.36	1.16E-20
SAOUHSC_02101	SAOUHSC_02101	Putative uncharacterized protein	-3.44	9.38E-17
SAOUHSC_02108	ftnA	Ferritin	-7.44	5.99E-15
SAOUHSC_02109	SAOUHSC_02109	Putative uncharacterized protein	-6.43	1.18E-14
SAOUHSC_02110	SAOUHSC_02110	Putative uncharacterized protein	-2.83	7.53E-18
SAOUHSC_02111	dinB	DNA polymerase IV	-4.62	5.01E-28
SAOUHSC_02112	SAOUHSC_02112	Putative uncharacterized protein	-2.06	2.73E-04
SAOUHSC_02115	SAOUHSC_02115	Putative uncharacterized protein	3.17	2.26E-04
SAOUHSC_02116	gatB	Aspartyl/glutamyl-tRNA(Asn/Gln) amidotransferase subunit B	2.07	1.22E-09
SAOUHSC_02117	gatA	Glutamyl-tRNA(Gln) amidotransferase subunit A	2.69	9.64E-12
SAOUHSC_02118	gatC	Aspartyl/glutamyl-tRNA(Asn/Gln) amidotransferase subunit C	5.92	9.83E-21
SAOUHSC_02119	putP	Sodium/proline symporter	2.01	1.01E-04
SAOUHSC_02126	purB	Adenylosuccinate lyase	3.42	7.98E-19
SAOUHSC_02129	SAOUHSC_02129	Putative uncharacterized protein	2.91	2.18E-03
SAOUHSC_02133	SAOUHSC_02133	Nicotinate phosphoribosyltransferase	2.38	4.86E-19
SAOUHSC_02135	SAOUHSC_02135	Putative uncharacterized protein	2.02	3.48E-13
SAOUHSC_02139	SAOUHSC_02139	Pyrazinamidase/nicotinamidase, putative	4.03	4.87E-30
SAOUHSC_02140	ppaC	Probable manganese-dependent inorganic pyrophosphatase	-2.62	9.43E-14
SAOUHSC_02141	SAOUHSC_02141	Putative uncharacterized protein	6.63	6.37E-03
SAOUHSC_02142	SAOUHSC_02142	Aldehyde dehydrogenase	2.40	5.12E-13
SAOUHSC_02146	SAOUHSC_02146	Putative uncharacterized protein	3.80	6.22E-12
SAOUHSC_02147	SAOUHSC_02147	Putative uncharacterized protein	2.27	2.69E-10
SAOUHSC_02148	SAOUHSC_02148	Putative uncharacterized protein	2.63	2.81E-11
SAOUHSC_02149	SAOUHSC_02149	Putative uncharacterized protein	5.36	2.56E-28
SAOUHSC_02150	SAOUHSC_02150	Putative uncharacterized protein	2.26	1.19E-15
SAOUHSC_02157	SAOUHSC_02157	Putative uncharacterized protein	-3.37	7.70E-10
SAOUHSC_02170	SAOUHSC_02170	Peptidoglycan hydrolase, putative	-4.29	1.09E-33
SAOUHSC_02171	SAOUHSC_02171	Staphylokinase, putative	-2.15	3.63E-06
SAOUHSC_02175	SAOUHSC_02175	Hypothetical phage protein	-7.04	8.85E-41
SAOUHSC_02176	SAOUHSC_02176	Conserved hypothetical phage protein	-7.42	3.03E-43
SAOUHSC_02179	SAOUHSC_02179	Conserved hypothetical phage protein	-3.66	5.39E-05
SAOUHSC_02180	SAOUHSC_02180	Phage minor structural protein, N-terminal region domain prot	-4.19	1.13E-28
SAOUHSC_02181	SAOUHSC_02181	Phi PVL orfs 18-19-like protein	-2.26	1.72E-03
SAOUHSC_02182	SAOUHSC_02182	Tail length tape measure protein	-4.71	7.70E-23
SAOUHSC_02241	SAOUHSC_02241	Uncharacterized leukocidin-like protein 1	-2.54	2.20E-13
SAOUHSC_02243	SAOUHSC_02243	Uncharacterized leukocidin-like protein 2	-2.31	1.02E-06
SAOUHSC_02254	groL	60 kDa chaperonin	-4.48	1.68E-16
SAOUHSC_02257	SAOUHSC_02257	Putative uncharacterized protein	3.68	6.84E-20
SAOUHSC_02260	hld	Delta-hemolysin	-6.14	4.41E-37
SAOUHSC_02264	SAOUHSC_02264	Accessory gene regulator protein C	-4.13	6.84E-35
SAOUHSC_02265	SAOUHSC_02265	Accessory gene regulator protein A	-3.82	3.93E-28
SAOUHSC_02266	SAOUHSC_02266	Putative uncharacterized protein	-2.89	6.73E-15
SAOUHSC_02268	SAOUHSC_02268	Sucrose-6-phosphate dehydrogenase, putative	2.05	2.41E-10
SAOUHSC_02269	SAOUHSC_02269	Sucrose operon repressor, putative	2.23	1.91E-05
SAOUHSC_02274	SAOUHSC_02274	ABC transporter, ATP-binding protein, putative	3.95	2.07E-17
SAOUHSC_02278	SAOUHSC_02278	Ribosomal-protein-alanine acetyltransferase	2.80	6.27E-17
SAOUHSC_02279	SAOUHSC_02279	Putative uncharacterized protein	2.62	3.70E-17
SAOUHSC_02280	SAOUHSC_02280	Putative uncharacterized protein	2.68	3.40E-12
SAOUHSC_02294	SAOUHSC_02294	Putative uncharacterized protein	2.20	5.47E-05
SAOUHSC_02298	SAOUHSC_02298	RNA polymerase sigma factor	2.56	6.23E-16
SAOUHSC_02299	rsbW	Serine-protein kinase RsbW	2.82	1.94E-21
SAOUHSC_02300	rsbV	Anti-sigma-B factor antagonist	2.59	8.91E-19
SAOUHSC_02301	SAOUHSC_02301	SigmaB regulation protein RsbU, putative	2.08	3.54E-06
SAOUHSC_02306	acpS	Holo-[acyl-carrier-protein] synthase	2.52	1.88E-13

SAOUHSC_02307	SAOUHSC_02307	Putative uncharacterized protein	3.07	2.97E-24
SAOUHSC_02308	SAOUHSC_02308	Putative uncharacterized protein	3.11	1.33E-27
SAOUHSC_02309	SAOUHSC_02309	Putative uncharacterized protein	3.20	3.41E-15
SAOUHSC_02314	SAOUHSC_02314	Sensor protein KdpD, putative	3.57	3.70E-17
SAOUHSC_02315	SAOUHSC_02315	DNA-binding response regulator, putative	3.14	1.26E-08
SAOUHSC_02319	SAOUHSC_02319	Putative uncharacterized protein	2.75	1.48E-17
SAOUHSC_02320	SAOUHSC_02320	Putative uncharacterized protein	-2.60	1.96E-12
SAOUHSC_02323	cls	Cardiolipin synthase	3.10	1.19E-15
SAOUHSC_02333	sceD	Probable transglycosylase SceD	10.84	4.34E-17
SAOUHSC_02335	SAOUHSC_02335	Putative uncharacterized protein	3.60	8.19E-09
SAOUHSC_02338	SAOUHSC_02338	Putative uncharacterized protein	4.13	1.94E-15
SAOUHSC_02340	atpC	ATP synthase epsilon chain	-2.87	3.54E-18
SAOUHSC_02341	atpD	ATP synthase subunit beta	-2.87	4.16E-15
SAOUHSC_02343	atpG	ATP synthase gamma chain	-2.46	8.02E-17
SAOUHSC_02349	atpE	ATP synthase subunit c	-2.38	2.74E-10
SAOUHSC_02350	atpB	ATP synthase subunit a	-2.31	7.99E-14
SAOUHSC_02351	SAOUHSC_02351	Putative uncharacterized protein	-2.07	9.56E-13
SAOUHSC_02352	SAOUHSC_02352	UDP-N-acetylglucosamine 2-epimerase	-2.28	2.22E-14
SAOUHSC_02357	SAOUHSC_02357	Putative uncharacterized protein	2.27	2.64E-08
SAOUHSC_02360	tdk	Thymidine kinase	2.27	1.43E-06
SAOUHSC_02364	SAOUHSC_02364	Putative uncharacterized protein	-5.72	1.30E-22
SAOUHSC_02365			-2.53	7.39E-16
SAOUHSC_02367	SAOUHSC_02367	Putative uncharacterized protein	2.99	1.99E-09
SAOUHSC_02372	SAOUHSC_02372	Putative uncharacterized protein	2.73	1.60E-12
SAOUHSC_02376	SAOUHSC_02376	Putative uncharacterized protein	-5.57	5.75E-49
SAOUHSC_02377	pdp	Pyrimidine-nucleoside phosphorylase	-6.11	6.41E-62
SAOUHSC_02378	SAOUHSC_02378	Putative uncharacterized protein	-5.34	1.13E-46
SAOUHSC_02379	deoC	Deoxyribose-phosphate aldolase	-4.44	1.27E-28
SAOUHSC_02381	SAOUHSC_02381	Putative uncharacterized protein	-12.02	1.55E-22
SAOUHSC_02383	SAOUHSC_02383	Putative uncharacterized protein	3.58	5.68E-31
SAOUHSC_02384	SAOUHSC_02384	Putative uncharacterized protein	3.17	6.13E-08
SAOUHSC_02385	SAOUHSC_02385	Mannose-6-phosphate isomerase, class I	3.97	9.25E-25
SAOUHSC_02386	SAOUHSC_02386	Putative uncharacterized protein	3.94	2.90E-16
SAOUHSC_02388	SAOUHSC_02388	Putative uncharacterized protein	-2.00	6.34E-06
SAOUHSC_02389	SAOUHSC_02389	Cation efflux family protein, putative	-2.71	2.03E-12
SAOUHSC_02390	SAOUHSC_02390	Lytic regulatory protein, putative	2.26	1.60E-09
SAOUHSC_02396	SAOUHSC_02396	Putative uncharacterized protein	-2.29	4.97E-13
SAOUHSC_02397	SAOUHSC_02397	ABC transporter, ATP-binding protein, putative	-2.79	8.33E-14
SAOUHSC_02399	glmS	Glutamine--fructose-6-phosphate aminotransferase [isomerizir	-2.40	2.16E-10
SAOUHSC_02404	SAOUHSC_02404	Putative uncharacterized protein	-5.82	8.55E-50
SAOUHSC_02405	glmM	Phosphoglucosamine mutase	-3.83	4.87E-30
SAOUHSC_02419	sepA	Multidrug resistance efflux pump SepA	2.82	2.18E-07
SAOUHSC_02426	SAOUHSC_02426	Membrane protein, putative	-2.10	3.41E-11
SAOUHSC_02444	SAOUHSC_02444	Osmoprotectant transporter, BCCT family, opuD-like protein, p	5.53	2.42E-11
SAOUHSC_02445	SAOUHSC_02445	Putative uncharacterized protein	-2.81	1.04E-11
SAOUHSC_02447	SAOUHSC_02447	Putative uncharacterized protein	-4.21	6.38E-25
SAOUHSC_02457	cobB	NAD-dependent protein deacetylase	-2.34	1.03E-06
SAOUHSC_02458	SAOUHSC_02458	Putative uncharacterized protein	2.46	9.44E-03
SAOUHSC_02459	SAOUHSC_02459	Putative uncharacterized protein	5.71	5.24E-08
SAOUHSC_02466	SAOUHSC_02466	Truncated MHC class II analog protein	-2.61	4.67E-11
SAOUHSC_02467	SAOUHSC_02467	Alpha-acetolactate decarboxylase	-7.63	4.34E-39
SAOUHSC_02468	SAOUHSC_02468	Acetolactate synthase, putative	-3.95	1.86E-29
SAOUHSC_02477	rpsI	30S ribosomal protein S9	-2.08	2.53E-11
SAOUHSC_02484	rplQ	50S ribosomal protein L17	-2.91	3.32E-24
SAOUHSC_02485	rpoA	DNA-directed RNA polymerase subunit alpha	-2.65	1.79E-22
SAOUHSC_02486	rpsK	30S ribosomal protein S11	-2.89	4.98E-23
SAOUHSC_02487	rpsM	30S ribosomal protein S13	-2.21	3.13E-14
SAOUHSC_02492	rplO	50S ribosomal protein L15	-2.09	4.38E-11
SAOUHSC_02494	rpsE	30S ribosomal protein S5	-2.06	2.78E-08
SAOUHSC_02496	rplF	50S ribosomal protein L6	-2.41	8.59E-12
SAOUHSC_02505	rplP	50S ribosomal protein L16	-2.02	4.42E-07
SAOUHSC_02506	rpsC	30S ribosomal protein S3	-2.11	9.78E-07
SAOUHSC_02509	rplB	50S ribosomal protein L2	-2.72	1.13E-10
SAOUHSC_02510	rplW	50S ribosomal protein L23	-2.71	1.88E-12
SAOUHSC_02511	rplD	50S ribosomal protein L4	-2.65	8.22E-14
SAOUHSC_02518	SAOUHSC_02518	Putative uncharacterized protein	2.84	7.94E-04
SAOUHSC_02520	SAOUHSC_02520	Sugar transporter, putative	2.52	6.23E-06
SAOUHSC_02521	SAOUHSC_02521	Putative uncharacterized protein	3.02	1.34E-03
SAOUHSC_02523	SAOUHSC_02523	Putative uncharacterized protein	2.89	6.79E-04
SAOUHSC_02524	SAOUHSC_02524	Putative uncharacterized protein	3.39	2.01E-15
SAOUHSC_02529	SAOUHSC_02529	Putative uncharacterized protein	2.19	1.49E-07
SAOUHSC_02532	sarV	HTH-type transcriptional regulator SarV	-3.03	5.31E-17
SAOUHSC_02536	moaA	Cyclic pyranopterin monophosphate synthase	-2.81	1.57E-15
SAOUHSC_02537	mobA	Probable molybdenum cofactor guanylyltransferase	-3.16	6.31E-21
SAOUHSC_02538	SAOUHSC_02538	Molybdopterin converting factor, subunit 1	-2.58	5.96E-14
SAOUHSC_02540	SAOUHSC_02540	Molybdopterin converting factor moa, putative	-2.47	2.91E-13
SAOUHSC_02541	SAOUHSC_02541	Molybdopterin-guanine dinucleotide biosynthesis protein Mob	-2.44	2.92E-12
SAOUHSC_02542	SAOUHSC_02542	Molybdopterin biosynthesis protein moeA, putative	-2.89	6.27E-13
SAOUHSC_02543	moaC	Cyclic pyranopterin monophosphate synthase accessory protei	-2.62	8.12E-13
SAOUHSC_02544	SAOUHSC_02544	Molybdopterin biosynthesis moaB, putative	-3.10	8.63E-21
SAOUHSC_02549	SAOUHSC_02549	Molybdenum ABC transporter, periplasmic molybdate-binding	2.18	1.78E-14
SAOUHSC_02555	SAOUHSC_02555	Putative uncharacterized protein	-2.40	4.27E-05
SAOUHSC_02556	SAOUHSC_02556	Putative uncharacterized protein	-2.18	1.02E-03
SAOUHSC_02558	ureA	Urease subunit gamma	2.14	1.26E-09
SAOUHSC_02567	SAOUHSC_02567	Putative uncharacterized protein	-6.92	1.51E-17
SAOUHSC_02568	SAOUHSC_02568	Putative uncharacterized protein	-6.85	3.84E-22

SAOUHSC_02569	SAOUHSC_02569	Putative uncharacterized protein	-3.30	1.90E-12
SAOUHSC_02571	ssaA2	Staphylococcal secretory antigen ssaA2	4.76	9.26E-17
SAOUHSC_02575	SAOUHSC_02575	Putative uncharacterized protein	2.29	6.25E-05
SAOUHSC_02576	SAOUHSC_02576	Secretory antigen SsaA, putative	44.15	4.25E-65
SAOUHSC_02577	SAOUHSC_02577	Putative 2-hydroxyacid dehydrogenase SAOUHSC_02577	-4.27	1.86E-25
SAOUHSC_02583	SAOUHSC_02583	Transcriptional regulator, putative	-3.80	5.12E-24
SAOUHSC_02584	SAOUHSC_02584	Putative uncharacterized protein	-2.46	3.27E-17
SAOUHSC_02585	SAOUHSC_02585	Putative uncharacterized protein	3.36	2.06E-24
SAOUHSC_02586	SAOUHSC_02586	Putative uncharacterized protein	3.28	1.57E-05
SAOUHSC_02587	SAOUHSC_02587	Putative uncharacterized protein	5.70	1.63E-20
SAOUHSC_02588	SAOUHSC_02588	Putative uncharacterized protein	4.57	9.18E-11
SAOUHSC_02591	SAOUHSC_02591	Putative uncharacterized protein	2.30	6.83E-08
SAOUHSC_02592	SAOUHSC_02592	Putative uncharacterized protein	2.56	1.65E-08
SAOUHSC_02595	SAOUHSC_02595	Putative uncharacterized protein	2.14	2.64E-04
SAOUHSC_02599	SAOUHSC_02599	Hex regulon repressor, putative	3.39	1.32E-06
SAOUHSC_02600	SAOUHSC_02600	Putative uncharacterized protein	-3.52	2.99E-21
SAOUHSC_02602	SAOUHSC_02602	Putative uncharacterized protein	2.27	6.05E-04
SAOUHSC_02604	SAOUHSC_02604	Putative uncharacterized protein	-2.31	3.70E-16
SAOUHSC_02606	hutI	Imidazolonepropionase	-4.37	5.71E-31
SAOUHSC_02607	hutU	Urocanate hydratase	-4.94	3.68E-25
SAOUHSC_02608	SAOUHSC_02608	Putative uncharacterized protein	2.58	2.33E-05
SAOUHSC_02609	fosB	Metallothiol transferase FosB	4.29	1.16E-03
SAOUHSC_02610	hutG	Formimidoylglutamase	-2.04	2.89E-08
SAOUHSC_02611	lyrA	Lysostaphin resistance protein A	7.65	1.88E-40
SAOUHSC_02613	SAOUHSC_02613	Putative uncharacterized protein	2.11	1.88E-08
SAOUHSC_02614	SAOUHSC_02614	Aldose 1-epimerase, putative	-2.03	1.75E-04
SAOUHSC_02618	SAOUHSC_02618	Putative uncharacterized protein	2.31	1.36E-09
SAOUHSC_02619	SAOUHSC_02619	Putative uncharacterized protein	2.88	2.64E-11
SAOUHSC_02620	SAOUHSC_02620	Putative uncharacterized protein	-3.30	4.97E-19
SAOUHSC_02625	SAOUHSC_02625	Putative uncharacterized protein	2.64	2.87E-11
SAOUHSC_02628	SAOUHSC_02628	Putative uncharacterized protein	3.37	2.38E-20
SAOUHSC_02629	SAOUHSC_02629	Drug resistance transporter, EmrB/QacA subfamily, putative	-2.13	1.21E-10
SAOUHSC_02631	SAOUHSC_02631	Putative uncharacterized protein	3.50	3.70E-11
SAOUHSC_02635	tcaA	Membrane-associated protein TcaA	-2.32	1.91E-05
SAOUHSC_02636	SAOUHSC_02636	Putative uncharacterized protein	-37.53	1.02E-148
SAOUHSC_02637	SAOUHSC_02637	Putative uncharacterized protein	-29.03	1.88E-138
SAOUHSC_02638	SAOUHSC_02638	Putative uncharacterized protein	-55.07	5.14E-88
SAOUHSC_02640	hrtA	Putative hemin import ATP-binding protein HrtA	-101.71	3.86E-92
SAOUHSC_02641	hrtB	Putative hemin transport system permease protein HrtB	-69.11	2.38E-84
SAOUHSC_02644	hssS	Heme sensor protein HssS	-2.06	6.41E-11
SAOUHSC_02647			-4.61	4.97E-26
SAOUHSC_02649	SAOUHSC_02649	Putative uncharacterized protein	2.22	5.19E-11
SAOUHSC_02650	SAOUHSC_02650	Uncharacterized lipoprotein SAOUHSC_02650	-2.46	8.73E-12
SAOUHSC_02653	SAOUHSC_02653	Putative uncharacterized protein	2.01	1.03E-04
SAOUHSC_02661	SAOUHSC_02661	PTS system sucrose-specific IIBC component, putative	-2.41	2.46E-14
SAOUHSC_02662	SAOUHSC_02662	PTS system sucrose-specific IIBC component	-2.34	3.14E-07
SAOUHSC_02664	SAOUHSC_02664	Transcriptional regulator, putative	2.67	3.65E-07
SAOUHSC_02666	SAOUHSC_02666	Putative uncharacterized protein	6.09	1.38E-27
SAOUHSC_02667	SAOUHSC_02667	Putative uncharacterized protein	4.11	1.10E-41
SAOUHSC_02668	SAOUHSC_02668	Putative uncharacterized protein	3.40	3.49E-20
SAOUHSC_02669	sarZ	HTH-type transcriptional regulator SarZ	4.75	8.00E-19
SAOUHSC_02670	SAOUHSC_02670	Putative uncharacterized protein	2.73	8.81E-16
SAOUHSC_02671	narT	Probable nitrate transporter NarT	-2.58	2.46E-12
SAOUHSC_02682	SAOUHSC_02682	Uroporphyrin-III C-methyltransferase, putative	-3.39	2.45E-30
SAOUHSC_02683	SAOUHSC_02683	Assimilatory nitrite reductase [NAD(P)H], small subunit, putativ	-2.51	2.86E-19
SAOUHSC_02684	SAOUHSC_02684	Assimilatory nitrite reductase [NAD(P)H], large subunit, putativ	-2.17	5.90E-15
SAOUHSC_02686	SAOUHSC_02686	Putative uncharacterized protein	2.11	5.13E-03
SAOUHSC_02688	SAOUHSC_02688	Putative uncharacterized protein	4.09	7.25E-06
SAOUHSC_02689	SAOUHSC_02689	Putative uncharacterized protein	5.20	2.00E-11
SAOUHSC_02691	SAOUHSC_02691	Putative uncharacterized protein	-5.69	6.69E-18
SAOUHSC_02692	SAOUHSC_02692	Putative uncharacterized protein	-5.23	1.36E-15
SAOUHSC_02696	SAOUHSC_02696	FmhA protein, putative	2.68	2.18E-12
SAOUHSC_02700	SAOUHSC_02700	Putative uncharacterized protein	-2.00	2.34E-05
SAOUHSC_02702	SAOUHSC_02702	Putative uncharacterized protein	-2.13	7.11E-04
SAOUHSC_02709	hlgC	Gamma-hemolysin component C	-3.42	1.13E-08
SAOUHSC_02710	hlgB	Gamma-hemolysin component B	-3.31	7.74E-12
SAOUHSC_02721	SAOUHSC_02721	Putative uncharacterized protein	-8.30	8.83E-28
SAOUHSC_02723	SAOUHSC_02723	Glycerate kinase, putative	-3.06	4.38E-17
SAOUHSC_02724	SAOUHSC_02724	Putative uncharacterized protein	-3.90	4.24E-30
SAOUHSC_02725	SAOUHSC_02725	Putative uncharacterized protein	2.02	1.42E-03
SAOUHSC_02731	SAOUHSC_02731	Putative uncharacterized protein	2.73	7.23E-09
SAOUHSC_02736	flp	Protein flp	2.52	1.39E-09
SAOUHSC_02741	SAOUHSC_02741	Amino acid ABC transporter, permease protein, putative	-2.77	8.61E-28
SAOUHSC_02742	SAOUHSC_02742	Amino acid transporter, putative	-2.44	9.58E-20
SAOUHSC_02750	SAOUHSC_02750	Putative uncharacterized protein	3.26	3.54E-24
SAOUHSC_02753	SAOUHSC_02753	Membrane protein, putative	-2.02	1.40E-03
SAOUHSC_02759	SAOUHSC_02759	Putative uncharacterized protein	-7.30	2.67E-19
SAOUHSC_02761	SAOUHSC_02761	Putative uncharacterized protein	2.72	1.86E-07
SAOUHSC_02771	SAOUHSC_02771	Putative uncharacterized protein	-2.50	2.96E-03
SAOUHSC_02780	SAOUHSC_02780	Putative uncharacterized protein	-2.14	3.72E-07
SAOUHSC_02781	SAOUHSC_02781	Putative uncharacterized protein	-3.36	5.11E-17
SAOUHSC_02782	SAOUHSC_02782	Putative uncharacterized protein	-2.39	5.62E-07
SAOUHSC_02790	SAOUHSC_02790	Putative uncharacterized protein	-2.87	1.25E-11
SAOUHSC_02791	SAOUHSC_02791	Pyrophosphohydrolase, putative	-3.35	6.35E-13
SAOUHSC_02793	pgcA	Phosphoglucomutase	2.27	2.80E-07
SAOUHSC_02794	SAOUHSC_02794	Putative uncharacterized protein	3.45	7.98E-13

SAOUHSC_02795	SAOUHSC_02795	Putative uncharacterized protein	3.35	5.70E-06
SAOUHSC_02802	SAOUHSC_02802	Fibronectin binding protein B, putative	-2.78	5.98E-07
SAOUHSC_02803	fnbA	Fibronectin-binding protein A	-4.28	1.04E-18
SAOUHSC_02806	SAOUHSC_02806	Gluconate permease, putative	-2.69	1.69E-10
SAOUHSC_02809	SAOUHSC_02809	Gluconate operon transcriptional repressor, putative	2.26	9.54E-04
SAOUHSC_02811	SAOUHSC_02811	Putative uncharacterized protein	2.21	8.67E-16
SAOUHSC_02813	SAOUHSC_02813	Putative uncharacterized protein	2.65	6.93E-08
SAOUHSC_02814	SAOUHSC_02814	Putative uncharacterized protein	3.65	6.90E-11
SAOUHSC_02815	SAOUHSC_02815	Putative uncharacterized protein	-2.20	1.70E-05
SAOUHSC_02816	SAOUHSC_02816	Putative uncharacterized protein	4.35	5.37E-17
SAOUHSC_02821	SAOUHSC_02821	Membrane spanning protein, putative	3.44	4.07E-03
SAOUHSC_02823	SAOUHSC_02823	Putative uncharacterized protein	2.20	6.48E-07
SAOUHSC_02824	SAOUHSC_02824	Putative uncharacterized protein	-5.01	4.22E-33
SAOUHSC_02825	SAOUHSC_02825	Putative uncharacterized protein	-3.52	2.24E-20
SAOUHSC_02828	SAOUHSC_02828	Putative uncharacterized protein	2.09	2.29E-09
SAOUHSC_02829	SAOUHSC_02829	Putative NAD(P)H nitroreductase SAOUHSC_02829	-2.30	9.78E-06
SAOUHSC_02830	SAOUHSC_02830	D-lactate dehydrogenase, putative	-3.56	3.34E-27
SAOUHSC_02831	SAOUHSC_02831	Putative uncharacterized protein	-2.24	7.87E-09
SAOUHSC_02832	SAOUHSC_02832	Putative uncharacterized protein	3.81	4.98E-10
SAOUHSC_02833	SAOUHSC_02833	Putative uncharacterized protein	2.13	1.32E-05
SAOUHSC_02834	SAOUHSC_02834	Sortase, putative	2.95	3.33E-15
SAOUHSC_02839	SAOUHSC_02839	L-serine dehydratase, iron-sulfur-dependent, alpha subunit	-4.71	9.38E-22
SAOUHSC_02840	SAOUHSC_02840	L-serine dehydratase, iron-sulfur-dependent, beta subunit	-2.56	1.13E-13
SAOUHSC_02841	SAOUHSC_02841	Putative uncharacterized protein	-2.48	3.38E-15
SAOUHSC_02843	SAOUHSC_02843	Putative uncharacterized protein	3.26	7.98E-11
SAOUHSC_02844	SAOUHSC_02844	Putative uncharacterized protein	-3.59	5.09E-15
SAOUHSC_02845	SAOUHSC_02845	Putative uncharacterized protein	-2.18	2.80E-07
SAOUHSC_02849	SAOUHSC_02849	Pyruvate oxidase, putative	-3.77	1.88E-11
SAOUHSC_02850	cidB	Holin-like protein CidB	-2.28	1.11E-04
SAOUHSC_02851	cidA	Holin-like protein CidA	-3.32	2.22E-11
SAOUHSC_02852	SAOUHSC_02852	Putative uncharacterized protein	3.40	1.40E-12
SAOUHSC_02853	SAOUHSC_02853	Putative uncharacterized protein	-6.89	4.70E-53
SAOUHSC_02855	SAOUHSC_02855	LysM domain protein	7.20	3.14E-15
SAOUHSC_02862	clpL	ATP-dependent Clp protease ATP-binding subunit ClpL	-3.30	1.64E-22
SAOUHSC_02863	SAOUHSC_02863	Putative uncharacterized protein	-2.98	2.72E-13
SAOUHSC_02865	SAOUHSC_02865	Putative uncharacterized protein	3.43	2.06E-07
SAOUHSC_02872	SAOUHSC_02872	Putative uncharacterized protein	-22.01	4.63E-46
SAOUHSC_02873	copA	Copper-exporting P-type ATPase A	-3.03	1.24E-16
SAOUHSC_02874	copZ	Copper chaperone CopZ	-3.00	5.10E-19
SAOUHSC_02877	crtN	Dehydrosqualene desaturase	2.15	3.44E-04
SAOUHSC_02879	crtM	Dehydrosqualene synthase	3.04	1.06E-04
SAOUHSC_02880	crtQ	4,4'-diaponeurosporenoate glycosyltransferase	2.32	3.09E-04
SAOUHSC_02881	crtP	Diapolycopene oxygenase	3.34	6.81E-06
SAOUHSC_02882	crtO	Glycosyl-4,4'-diaponeurosporenoate acyltransferase	5.29	1.00E-03
SAOUHSC_02883	ssaA	Staphylococcal secretory antigen SsaA	14.02	1.22E-07
SAOUHSC_02886	SAOUHSC_02886	Putative uncharacterized protein	11.13	1.18E-40
SAOUHSC_02887	isaA	Probable transglycosylase IsaA	15.80	1.67E-49
SAOUHSC_02888	SAOUHSC_02888	Putative uncharacterized protein	3.79	5.42E-06
SAOUHSC_02889	SAOUHSC_02889	Putative uncharacterized protein	12.74	9.38E-05
SAOUHSC_02891	SAOUHSC_02891	Putative uncharacterized protein	2.49	2.29E-06
SAOUHSC_02892	SAOUHSC_02892	Putative uncharacterized protein	-3.92	2.20E-14
SAOUHSC_02900	SAOUHSC_02900	Uncharacterized hydrolase SAOUHSC_02900	-2.28	2.88E-18
SAOUHSC_02905	SAOUHSC_02905	Putative uncharacterized protein	-3.34	3.99E-23
SAOUHSC_02906	SAOUHSC_02906	Putative uncharacterized protein	-4.33	1.28E-28
SAOUHSC_02907	SAOUHSC_02907	Putative uncharacterized protein	-5.40	2.53E-37
SAOUHSC_02909	SAOUHSC_02909	Dihydroorotate dehydrogenase	5.41	3.65E-17
SAOUHSC_02910	SAOUHSC_02910	Putative uncharacterized protein	2.30	1.42E-06
SAOUHSC_02912	SAOUHSC_02912	Putative uncharacterized protein	-2.08	1.00E-08
SAOUHSC_02921	SAOUHSC_02921	Alpha-acetolactate decarboxylase	-4.76	2.15E-22
SAOUHSC_02922	ldh2	L-lactate dehydrogenase 2	2.08	4.79E-06
SAOUHSC_02923	SAOUHSC_02923	Putative uncharacterized protein	3.03	4.56E-06
SAOUHSC_02925	SAOUHSC_02925	Putative uncharacterized protein	3.79	6.80E-04
SAOUHSC_02926	fda	Fructose-bisphosphate aldolase class 1	3.60	8.17E-37
SAOUHSC_02930	SAOUHSC_02930	Putative uncharacterized protein	-2.61	4.64E-09
SAOUHSC_02932	betA	Oxygen-dependent choline dehydrogenase	5.43	2.97E-21
SAOUHSC_02933	SAOUHSC_02933	Betaine aldehyde dehydrogenase	3.60	2.91E-23
SAOUHSC_02934	SAOUHSC_02934	Putative uncharacterized protein	33.15	6.53E-15
SAOUHSC_02935	SAOUHSC_02935	Putative uncharacterized protein	3.04	3.53E-16
SAOUHSC_02941	SAOUHSC_02941	Anaerobic ribonucleoside-triphosphate reductase-activating pr	2.20	2.25E-10
SAOUHSC_02942	SAOUHSC_02942	Anaerobic ribonucleoside-triphosphate reductase, putative	2.73	2.22E-14
SAOUHSC_02943	SAOUHSC_02943	Citrate transporter, putative	3.18	3.66E-06
SAOUHSC_02944	SAOUHSC_02944	Putative uncharacterized protein	-2.04	8.77E-05
SAOUHSC_02945	SAOUHSC_02945	Siroheme synthase, putative	-2.23	9.60E-07
SAOUHSC_02950	SAOUHSC_02950	Putative uncharacterized protein	3.72	4.34E-03
SAOUHSC_02955	SAOUHSC_02955	Sensor histidine kinase, putative	-2.13	1.87E-08
SAOUHSC_02956	SAOUHSC_02956	DNA-binding response regulator, putative	-2.07	1.32E-08
SAOUHSC_02964	arcR	HTH-type transcriptional regulator ArcR	-6.65	4.60E-55
SAOUHSC_02965	arcC2	Carbamate kinase 2	-6.75	1.68E-85
SAOUHSC_02967	SAOUHSC_02967	Arginine/ornithine antiporter, putative	-7.48	2.91E-81
SAOUHSC_02968	argF	Ornithine carbamoyltransferase	-6.45	1.01E-76
SAOUHSC_02969	arcA	Arginine deiminase	-5.89	2.26E-58
SAOUHSC_02970			-2.83	6.52E-07
SAOUHSC_02971	SAOUHSC_02971	Aureolysin, putative	-2.07	9.32E-10
SAOUHSC_02972	isaB	Immunodominant staphylococcal antigen B	-2.91	1.59E-10
SAOUHSC_02991	SAOUHSC_02991	Putative uncharacterized protein	-2.02	1.21E-04
SAOUHSC_02994	SAOUHSC_02994	Putative uncharacterized protein	-2.58	4.18E-11

SAOUHSC_03002	icaA	Poly-beta-1,6-N-acetyl-D-glucosamine synthase	3.16	1.29E-03
SAOUHSC_03006	lipA	Lipase 1	-4.27	5.00E-15
SAOUHSC_03008	SAOUHSC_03008	Imidazole glycerol phosphate synthase subunit hisF	-4.17	7.18E-41
SAOUHSC_03009	hisA	1-(5-phosphoribosyl)-5-[(5-phosphoribosylamino)methylidenea	-2.63	6.67E-12
SAOUHSC_03015	hisZ	ATP phosphoribosyltransferase regulatory subunit	3.74	9.93E-05
SAOUHSC_03016	SAOUHSC_03016	Putative uncharacterized protein	2.20	1.67E-12
SAOUHSC_03017	SAOUHSC_03017	Putative uncharacterized protein	3.66	1.32E-04
SAOUHSC_03018	SAOUHSC_03018	Putative uncharacterized protein	2.12	3.80E-03
SAOUHSC_03023	drp35	Lactonase drp35	-2.33	3.13E-12
SAOUHSC_03024	SAOUHSC_03024	UPF0176 protein SAOUHSC_03024	-2.78	2.42E-20
SAOUHSC_03031	SAOUHSC_03031	Putative uncharacterized protein	3.79	8.52E-14
SAOUHSC_03032	SAOUHSC_03032	Putative uncharacterized protein	2.86	8.96E-04
SAOUHSC_03033	SAOUHSC_03033	High affinity nickel transporter, putative	2.44	3.88E-10
SAOUHSC_03035	SAOUHSC_03035	Putative uncharacterized protein	-4.64	5.26E-14
SAOUHSC_03049	SAOUHSC_03049	Putative uncharacterized protein	-2.12	1.19E-05
SAOUHSC_03051	rsmG	Ribosomal RNA small subunit methyltransferase G	-2.86	3.21E-09
SAOUHSC_03052	mnmG	tRNA uridine 5-carboxymethylaminomethyl modification enzym	-2.66	1.28E-09
SAOUHSC_03053	mnmE	tRNA modification GTPase MnmE	-2.01	1.54E-07
SAOUHSC_03055	rpmH	50S ribosomal protein L34	-3.07	5.21E-19
SAOUHSC_A00084	SAOUHSC_00083.1	Putative uncharacterized protein	-2.08	2.13E-04
SAOUHSC_A00219	SAOUHSC_00221.1	Putative uncharacterized protein	4.06	1.39E-07
SAOUHSC_A00283	SAOUHSC_00284.1	Putative uncharacterized protein	2.95	3.51E-04
SAOUHSC_A00635	SAOUHSC_00661.1	Putative uncharacterized protein	2.69	6.57E-04
SAOUHSC_A01436	SAOUHSC_01493.1	Putative uncharacterized protein	2.64	8.08E-03
SAOUHSC_A02013	SAOUHSC_02102.1	Putative uncharacterized protein	2.04	1.04E-03
SAOUHSC_A02169	SAOUHSC_02280.1	Putative uncharacterized protein	3.41	1.90E-06
SAOUHSC_A02189	SAOUHSC_02302.1	Putative uncharacterized protein	2.51	3.92E-07
SAOUHSC_A02445	SAOUHSC_02576.1	Putative uncharacterized protein	16.35	1.39E-15
SAOUHSC_A02771	SAOUHSC_02922.1	Putative uncharacterized protein	8.72	5.12E-04
SAOUHSC_A02856	SAOUHSC_03006.1	Putative uncharacterized protein	-3.31	3.76E-06
SAOUHSC_R00011			-4.13	8.44E-04
SAOUHSC_R00012			-4.40	5.06E-04
SAOUHSC_T00012			2.40	4.90E-08
SAOUHSC_T00026			2.20	1.03E-06
SAOUHSC_T00038			2.69	1.18E-07
SAOUHSC_T00042			2.25	4.88E-07
SAOUHSC_T00055			-2.06	7.49E-04

Video S1. Biofilm formation of strain *S. aureus* IPLA 1 in the presence of phage phiIPLA-RODI at an MOI of 10^{-3} (10^{3} <pFU/well) monitored in real time for 7 hours by confocal laser scanning microscopy.
